# Antiviral cellular therapy for enhancing T-cell reconstitution before or after hematopoietic stem cell transplantation (ACES): a two-arm, open label phase II interventional trial of pediatric patients with risk factor assessment

**DOI:** 10.1038/s41467-024-47057-2

**Published:** 2024-04-18

**Authors:** Michael D. Keller, Patrick J. Hanley, Yueh-Yun Chi, Paibel Aguayo-Hiraldo, Christopher C. Dvorak, Michael R. Verneris, Donald B. Kohn, Sung-Yun Pai, Blachy J. Dávila Saldaña, Benjamin Hanisch, Troy C. Quigg, Roberta H. Adams, Ann Dahlberg, Shanmuganathan Chandrakasan, Hasibul Hasan, Jemily Malvar, Mariah A. Jensen-Wachspress, Christopher A. Lazarski, Gelina Sani, John M. Idso, Haili Lang, Pamela Chansky, Chase D. McCann, Jay Tanna, Allistair A. Abraham, Jennifer L. Webb, Abeer Shibli, Amy K. Keating, Prakash Satwani, Pawel Muranski, Erin Hall, Michael J. Eckrich, Evan Shereck, Holly Miller, Ewelina Mamcarz, Rajni Agarwal, Satiro N. De Oliveira, Mark T. Vander Lugt, Christen L. Ebens, Victor M. Aquino, Jeffrey J. Bednarski, Julia Chu, Suhag Parikh, Jennifer Whangbo, Michail Lionakis, Elias T. Zambidis, Elizabeth Gourdine, Catherine M. Bollard, Michael A. Pulsipher

**Affiliations:** 1 Center for Cancer & Immunology Research, Children’s National Hospital, Washington, DC USA; 2 Division of Allergy and Immunology, Children’s National Hospital, Washington, DC USA; 3 GW Cancer Center, George Washington University School of Medicine, Washington, DC USA; 4 Division of Blood and Marrow Transplantation, Children’s National Hospital, Washington, DC USA; 5 Department of Pediatrics and Preventative Medicine, University of Southern California, Los Angeles, CA USA; 6 Cancer and blood disease institute, Children’s Hospital of Los Angeles, Los Angeles, CA USA; 7 Division of Pediatric Allergy, Immunology, and BMT, University of California San Francisco, San Francisco, CA USA; 8 Department of Pediatrics and Division of Child’s Cancer and Blood Disorders, Children’s Hospital Colorado and University of Colorado, Denver, CO USA; 9 Department of Microbiology, Immunology & Molecular Genetics and Department of Pediatrics David Geffen School of Medicine, University of California, Los Angeles, Los Angeles, CA USA; 10 Division of Hematology/Oncology, University of California, Los Angeles, Los Angeles, CA USA; 11 Immune Deficiency Cellular Therapy Program, Center for Cancer Research, National Cancer Institute, Bethesda, MD USA; 12 Division of Pediatric Infectious Diseases, Children’s National Hospital, Washington, DC USA; 13 Pediatric Blood & Bone Marrow Transplant and Cellular Therapy, Helen DeVos Children’s Hospital, Grand Rapids, MI USA; 14 Center for Cancer and Blood Disorders, Phoenix Children’s/Mayo Clinic Arizona, Phoenix, AZ USA; 15 Clinical Research Division, Fred Hutch Cancer Center/Seattle Children’s Hospital/University of Washington, Seattle, WA USA; 16 Aflac Cancer and Blood Disorders Center, Children’s Healthcare of Atlanta, Atlanta, GA USA; 17 Division of Hematology, Children’s National Hospital, Washington, DC USA; 18 Pediatric Stem Cell Transplant, Dana-Farber Cancer Institute and Boston Children’s Hospital, Boston, MA USA; 19 Division of Pediatric Hematology/Oncology and Stem Cell Transplantation, Columbia University Medical Center, New York, NY USA; 20 Columbia Center for Translational Immunology, Columbia University Medical Center, New York, NY USA; 21 Division of Pediatric Hematology/Oncology/Bone Marrow Transplant, Children’s Mercy Kansas City, Kansas City, MO USA; 22 Pediatric Transplant and Cellular Therapy, Levine Children’s Hospital, Wake Forest School of Medicine, Charlotte, NC USA; 23 Division of Hematology and Oncology, Oregon Health & Science Univ, Portland, OR USA; 24 Department of Bone Marrow Transplantation and Cellular Therapy, St. Jude Children’s Research Hospital, Memphis, TN USA; 25 Division of Pediatric Hematology/Oncology, Stem Cell Transplantation and Regenerative Medicine, Stanford University, Palo Alto, CA USA; 26 Division of Pediatric Hematology/Oncology/BMT, C.S. Mott Children’s Hospital, University of Michigan, Ann Arbor, MI USA; 27 Division of Pediatric Blood and Marrow Transplant & Cellular Therapy, University of Minnesota MHealth Fairview Masonic Children’s Hospital, Minneapolis, MI USA; 28 Division of Pediatric Hematology/Oncology, University of Texas, Southwestern Medical Center Dallas, Dallas, TX USA; 29 Department of Pediatrics, Division of Pediatric Hematology and Oncology, Washington University School of Medicine, St Louis, MO USA; 30 Cancer and Blood Disorders Center, Dana Farber Institute and Boston Children’s Hospital, Boston, MA USA; 31 Laboratory of Clinical Immunology & Microbiology, National Institute of Allergy and Infectious Diseases, Bethesda, MD USA; 32 Pediatric Blood and Marrow Transplantation Program, Sidney Kimmel Comprehensive Cancer Center, Johns Hopkins University School of Medicine, Baltimore, MD USA; 33 Division of Pediatric Hematology/Oncology, Intermountain Primary Children’s Hospital, Huntsman Cancer Institute, Spencer Fox Eccles School of Medicine at the University of Utah, Salt Lake City, UT USA

**Keywords:** Translational research, Paediatric research, Allotransplantation, T cells

## Abstract

Viral infections remain a major risk in immunocompromised pediatric patients, and virus-specific T cell (VST) therapy has been successful for treatment of refractory viral infections in prior studies. We performed a phase II multicenter study (NCT03475212) for the treatment of pediatric patients with inborn errors of immunity and/or post allogeneic hematopoietic stem cell transplant with refractory viral infections using partially-HLA matched VSTs targeting cytomegalovirus, Epstein-Barr virus, or adenovirus. Primary endpoints were feasibility, safety, and clinical responses (>1 log reduction in viremia at 28 days). Secondary endpoints were reconstitution of antiviral immunity and persistence of the infused VSTs. Suitable VST products were identified for 75 of 77 clinical queries. Clinical responses were achieved in 29 of 47 (62%) of patients post-HSCT including 73% of patients evaluable at 1-month post-infusion, meeting the primary efficacy endpoint (>52%). Secondary graft rejection occurred in one child following VST infusion as described in a companion article. Corticosteroids, graft-versus-host disease, transplant-associated thrombotic microangiopathy, and eculizumab treatment correlated with poor response, while uptrending absolute lymphocyte and CD8 T cell counts correlated with good response. This study highlights key clinical factors that impact response to VSTs and demonstrates the feasibility and efficacy of this therapy in pediatric HSCT.

## Abstract (web summary)

Viral infection is a common risk for immune-compromised individuals, particularly pediatric patients receiving hematopoietic stem cell transplants. Here the authors report a phase II trial testing adoptive transfer of third party, virus-specific T cells on the feasibility, safety, clinical responses, as well as homeostasis of antiviral immunity in the recipients.

## Introduction

Despite advances in antiviral pharmacotherapy, viral infections are a serious threat to patients with T cell deficiency due to hematopoietic stem cell transplantation (HSCT). In pediatric patients, adenovirus and cytomegalovirus remain frequent causes of transplant-related mortality^1–8^. While new approaches of HSCT such as αβT cell receptor (TCR) depletion or the use of post-transplant cyclophosphamide have expanded potential donor pools^9–11^, these approaches cause delayed T cell reconstitution^12,13^, impairing critical antiviral defenses^14^. Similarly, patients with T cell deficiency due to inborn errors of immunity (IEI) such as severe combined immunodeficiency (SCID), have a high risk of chronic and potentially fatal viral infections prior to curative therapies^15–19^.

For over two decades, donor-derived virus-specific T cell (VST) therapy has been utilized to treat or prevent viral infections in patients following HSCT in many clinical trials worldwide^20–29^. Though VSTs derived from HSCT donors have the advantage of long-term persistence, they require an available and suitable donor for production as well as time and expense for customized VST production^24,30,31^. Use of banked, partially HLA (human leukocyte antigen)-matched VSTs produced from “third party” healthy donors circumvent the limitations of donor-derived products and allows for “off the shelf” therapy for patients with severe viral infections^32^. Prior single center or limited multicenter studies of third-party VST therapies have demonstrated efficacy against multiple viruses including cytomegalovirus, Epstein-Barr virus, adenovirus, and BK virus, but focused predominantly on adult patients^33–35^. Despite partial HLA matching, VSTs have been generally well tolerated in these studies, with low rates of de novo graft versus host disease (GVHD) and very low rates of infusion-related toxicities including cytokine release syndrome^24,33,36–40^.

Treatment of immunocompromised pediatric patients have been included in some previous studies^23,33,38,41–45^. However, no trial to date has centered primarily on treatment of immunocompromised pediatric patients with refractory viral infections. Accordingly, most prior studies have been skewed toward patients treated after HSCT for malignancies, with fewer patients treated for non-malignant diseases including IEI, which represents a larger proportion of pediatric HSCT and are associated with high risk of viral disease. Antiviral response rates in prior studies have been as high as 74–93%, but many of the highest acuity patients (ICU patients with organ failure) were not included in most prior phase I studies.

Here, we present the first pediatric-focused, multicenter, consortium-led phase II study of third-party VST therapy for patients with T cell deficiency due to HSCT or IEI, which aims to evaluate the safety and efficacy of VSTs in this setting. We show that VST therapy is feasible in a multicenter setting, as products are identified for 97% of referred patients and infusions given at a median of 1 week after bank query. In addition, antiviral responses strongly correlate with overall survival at 1 year. Finally, we show that T cell immune reconstitution improved in most responders, primarily arising from the HSCT graft, suggesting that VSTs largely play a supportive role in facilitating immune reconstitution.

## Results

### Patient characteristics and treatment

Sixty patients with refractory CMV, EBV, and/or adenovirus were enrolled and followed at 22 centers across the US between June 2018 and December 2021. Two patients had no suitable VST products identified, and 7 were not treated due to ineligibility following initial product query (3 due to improvement in viral load, 2 due to death, 1 due to high grade GVHD). Of 77 patient queries for infusion, suitable products were identified in 75 cases (97%). Fifty-one patients received 1-3 VST infusions for CMV (*n* = 17), EBV (*n* = 4), adenovirus (*n* = 24), or concurrent CMV and adenovirus (*n* = 6), which met goal accrual for the two primary strata. Twenty-eight patients had confirmed tissue disease due to viral infection, including pneumonitis, hepatitis, enteritis, and retinitis. Median age at the time of treatment was 8 years (Table 1). Forty-seven patients were treated following HSCT (Arm A), and 4 patients with IEI were treated prior to HSCT (Arm B). Indications for HSCT included relapsed malignancy (*n* = 22), IEI (*n* = 20), and other non-malignant diseases including sickle cell anemia (*n* = 9), beta thalassemia (*n* = 2), Fanconi anemia (*n* = 1), and severe aplastic anemia (*n* = 2). Underlying forms of IEI in the patients treated pre-HSCT were SCID (*n* = 2), complete Digeorge syndrome (*n* = 1), and TTC7A deficiency (*n* = 1). Most patients had received T cell depletion as part of their pre-conditioning, including αβTCR/CD19 depletion + ATG (*n* = 10), ATG alone (*n* = 12), and alemtuzumab (*n* = 13).

**Table 1:** Patient demographics

Criteria	Value
Male gender	25 (49%)
Median age (range)	8 yrs (1 month–23 yrs)
Underlying diagnosis	
Malignancy	22 (43%)
Inborn Error of Immunity	20 (39%)
Other non-malignant condition^a^	9 (18%)
HSCT donor types	
MSD	7 (14%)
URD	16 (31%)
MMRD	18 (35%)
UCB	6 (12%)
Pre-HSCT	4 (8%)
T cell depletion	
αβTCR/CD19 depletion + ATG	10 (20%)
ATG alone	12 (24%)
Alemtuzumab	13 (25%)
Targeted virus	
CMV	17 (33%)
EBV	4 (8%)
Adenovirus	24 (47%)
CMV+Adenovirus	6 (12%)
Tissue disease at time of infusion	
Yes	28 (55%)
No	23 (45%)
Pre-infusion GVHD	
Yes	21 (41%)
No	30 (59%)
Acuity at first VST infusion	
ICU	11(22%)
Non-ICU	40(78%)

^a^sickle cell anemia (*n* = 3), beta thalassemia (*n* = 2), Fanconi anemia (*n* = 1), and severe aplastic anemia (*n* = 2).

Patients on Arm A were treated at a median of 84 days post-HSCT (range 16–476). Forty-seven patients had received 1–3 antiviral therapies, and four patients were treated prior to antiviral therapy due to poor renal function (Supplementary Table 1, Supplementary Figs. 1–5). Co-morbidities were common at the time of infusion, including respiratory failure (*n* = 8), renal failure (*n* = 12), transplant-associated thrombotic microangiopathy (TA-TMA, *n* = 10), hepatic veno-occlusive disease (VOD, *n* = 5), and history of GVHD (*n* = 21). Eleven patients required ICU care, and one patient was treated while receiving extracorporeal membrane oxygenation.

### Treatment, toxicities, and clinical responses

**Fig. 1:**
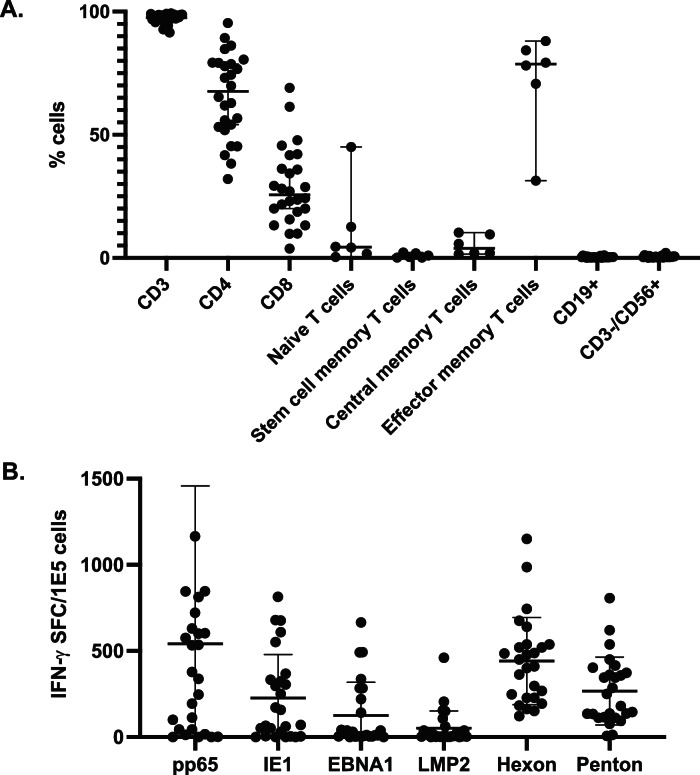
Phenotype and antiviral activity of Virus-specific T cell products (*n* = 26). **A** Cellular phenotype of VST products by flow cytometry. T cell memory sub-phenotyping (*n* = 6): Stem cell memory: CD45RO−/CCR7 + /CD95+; Central memory: CD45RO+/CCR7+; Effector memory: CD45RO+/CCR7−. **B** Antiviral responses of VSTs against CMV (pp65, IE1), EBV (EBNA1, LMP2), and adenovirus (Hexon, Penton) antigens by IFN-g ELISpot. SFC Spot forming colonies. Line: median values; whiskers: standard deviation.

Sixty-nine infusions were administered to 51 patients utilizing 26 partially HLA-matched VST products (Table 2). Thirty-six patients received a single infusion, 12 patients received two infusions, and 3 patients received 3 infusions. Infusions occurred at a median of 7 days following bank query (range 2–39). Infused VSTs were almost exclusively T cells (median 97.5%, range 91.5–99.3%) with mixed CD4 (median 70%, range 38–95.4%) and CD8 (median 26%, range 4–61.4%) populations, and predominant effector memory (median 78.7% CD45RO^+^/CCR7^−^/CD62L^−^, range 31–88%) with smaller central memory subsets (median 3.8% CD45RO^+^/CCR7^+^/CD62L^+^, range 1.5–10.3%, Fig. 1A). VSTs showed specificity against a median of 4 of 6 targeted viral antigens (range 2–6, Fig. 1B). VSTs were HLA matched with recipients at a median of 3 alleles (range 1–6). Fifty-one of 69 infusions (74%) had confirmation of viral specificity through shared HLA alleles between the VST donor and recipient, and 5 were guided by predicted epitopes, with the remainder guided by best HLA match.

**Table 2:** Antiviral responses for evaluable patients at 1 month post-VST infusion

Treatment Arm	Factors	Category	Total patients	# evaluable patients	# CR/PR patients (%)	Median (range) months to response (CR/PR only)
A	Primary Virus	Adv	29	23	17 (74%)	0.99 (0.07–6.83)
		CMV	20	13	10 (77%)	1.53 (0.20–5.35)
		EBV	4	4	2 (50%)	2.18 (0.23–4.14)
	Acuity	Normal	29	25	19 (76%)	0.99 (0.07–5.35)
		High	18	15	10 (67%)	1.89 (0.33–6.83)
	Total		47	40	29 (73%)	1.22 (0.07–6.83)
B	Primary Virus	Adv	1	0	0 (0%)	N/A
		CMV	3	2	0 (0%)	N/A
		EBV	0	0	0 (0%)	N/A
	Acuity	Normal	2	2	0 (0%)	N/A
		High	2	0	0 (0%)	N/A
	Total		4	2	0 (0%)	N/A

**Table 3:** Clinical response by HSCT timing

Variable		Response			*p*-value
		CR/PR	NR	Total	
Timing of treatment (1st infusion - HSCT)	Early (<84 days)	16 (84%)	3 (16%)	19 (100%)	0.037
	Late (≥84 days)	13 (54%)	11 (46%)	24 (100%)	
	Total	29 (67%)	14 (33%)	43 (100%)	

*p*-value from 1-sided Fisher’s exact test.

VST infusions were generally well tolerated, with few dose-limiting toxicities (Supplementary Table 2). Grade III cytokine release syndrome occurred in one patient (Patient 39) at day +10 with elevation in plasma IL-6 and IL-8 (Supplementary Fig. 6A), requiring treatment with tocilizumab and steroids. Patient 14 also received tocilizumab at day +6 due to worsening hypoxemia and hypotension, which was eventually attributed to worsening CMV pneumonitis. Four patients had flaring of pre-existing acute GVHD following VST infusion at a median time of 26 days (range 13–63), and one patient was diagnosed with chronic GVHD at 9 months post VST infusion, which improved with therapy. Secondary graft rejection occurred in three patients, one of which was caused by passenger lymphocytes following liver transplantation for refractory VOD (Patient 18), and another in the setting of autologous reconstitution (Patient 50). In one case rejection was associated with VST expansion and felt to be related to VST infusion (Patient 24, see the companion article (ref. ^46^)). Progressive neurologic disease, including seizures and diffuse axonal polyneuropathy, occurred in two patients following VST infusion; both patients had disseminated adenoviral infection including one patient who had documented adenovirus in the CSF (Patient 43). The other patient had a history of TA-TMA (Patient 46). Both patients had elevations of sIL2R, and patient 43 had elevation of IL-6 (Supplementary Fig. 6B, C). Treatment with steroids and siltuximab failed to improve their clinical status (Supplementary Fig. 5).

**Fig. 2:**
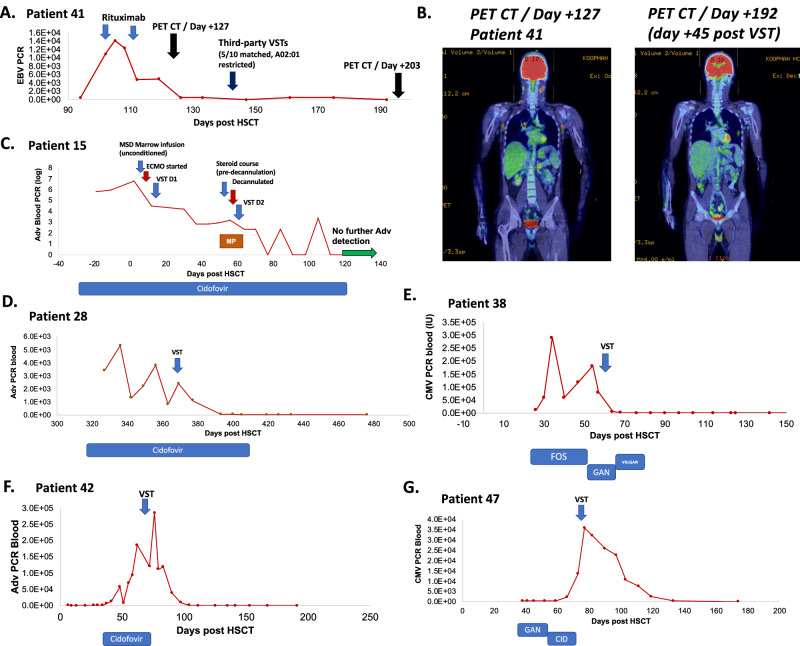
Longitudinal viral loads and antiviral medications post VST therapy. **A** EBV viral loads (red) and Rituxan use in patient 41, who received VST therapy on day +147. **B** PET CT (positron emission tomography/computed tomography) imaging before and after VST therapy in Patient 41. **C** Adenoviral load (red) in Patient 15, who required extracorporeal membrane oxygenation (ECMO) for severe adenoviral disease after unconditioned matched sibling marrow infusion, and subsequent methylprednisolone (MP) course prior to de-cannulation. **D**–**G** Viral loads pre and post- VST therapy and antiviral medications over time in study patients 28, 38, 42, and 47 (FOS foscarnet, GAN ganciclovir, VALGAN valganciclovir, CID cidofovir).

Of the 47 patients treated after HSCT (Arm A), 40 had evaluable data at 1 month following VST infusion. Twenty-nine (73%) patients demonstrated an antiviral response based on viral PCR, at a median time of 37 days post-infusion (Table 2, Fig. 2A–G). For patients treated for CMV, 10 of 13 responded (7 CR, 3 PR). In patients treated for adenovirus, 17 of 23 had antiviral responses (15 CR, 2 PR). In 6 patients with both CMV and adenovirus at the time of VST infusion, 3 cleared both viruses, and another cleared adenovirus but not CMV. Two of four patients with EBV responded to VST therapy (1 CR, 1 PR). Of the 4 patients on Arm B who were treated prior to HSCT, none achieved an antiviral response, and three died of progressive viral disease. Given the wide range of treatment timing post-HSCT in Arm B, we performed sub analysis of clinical response rates in patients treated before or after the median day post-HSCT (84 days) shows a higher rate of responses among patients treated earlier (84% *n* = 16/19) versus later (54%, *n* = 13/24, Table 3, *p* = 0.037).

**Fig. 3:**
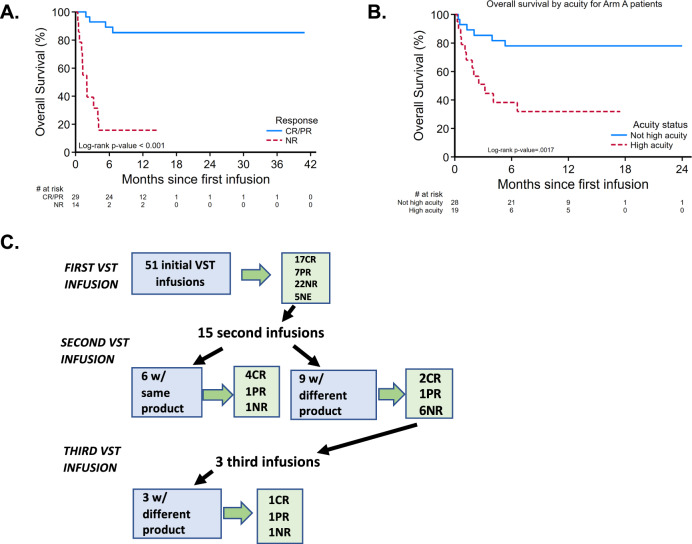
Overall survival and treatment schema in study patients. **A** Overall survival in responders (CR complete responders, PR partial responders) versus non-responders (NR) following VST therapy (*n* = 51, survival curves were compared by log-rank test, *p* = 1.06 × 10^−7^). **B** Overall survival by patient acuity following VST infusion for Arm A (*n* = 47, *p* = 0.0017). High acuity was defined as infusion in the intensive care unit and/or respiratory failure, renal failure, veno-occlusive disease, or transplant-associated microangiopathy. Survival curves were compared by log-rank test. **C** Infusion schema and responses by number of VST doses and product details. Patients without data at day +28 post-infusion are listed as not evaluable (NE).

Overall survival was 57.1% (95% CI: 42.00–70.00%) at 1 year, with 1-year survival of 63.2% (95% CI: 46.40–76.00%) in Arm A. Overall survival was strongly linked to antiviral responses, with 85.3% (95% CI: 65.30–94.20%) of those achieving a CR or PR alive at 1 year (Fig. 3A). Thirteen patients died from progressive viral disease, and 4 patients died from unrelated infections. Patients in Arm A who were considered high acuity (based on a composite variable including ICU admission, respiratory or renal failure, VOD or TA-TMA) had worse survival than normal acuity patients, with 7 of 19 alive at 12 months post-infusion, compared to 22 of 28 (Fig. 3B, *p* = 0.0017). Fifteen patients received more than one infusion due to incomplete antiviral response (Fig. 3C). In those receiving a second infusion, 6 were given the same VST product, with 5 achieving clinical responses (4 CR, 1 PR). Nine patients received second infusions from a different VST donor, with 3/9 achieving subsequent clinical responses (2 CR, 1 PR). Three patients received a third infusion, all of which were from a different VST donor than the previous infusion, resulting in 1 complete and 1 partial response. Of the 9 patients who were screened but not treated, 3 died of progressive viral disease, and 4 remained alive at 12 months following screening.

### Immunosuppression and transplantation-associated toxicities impact chances of antiviral response after VST infusion

**Fig. 4:**
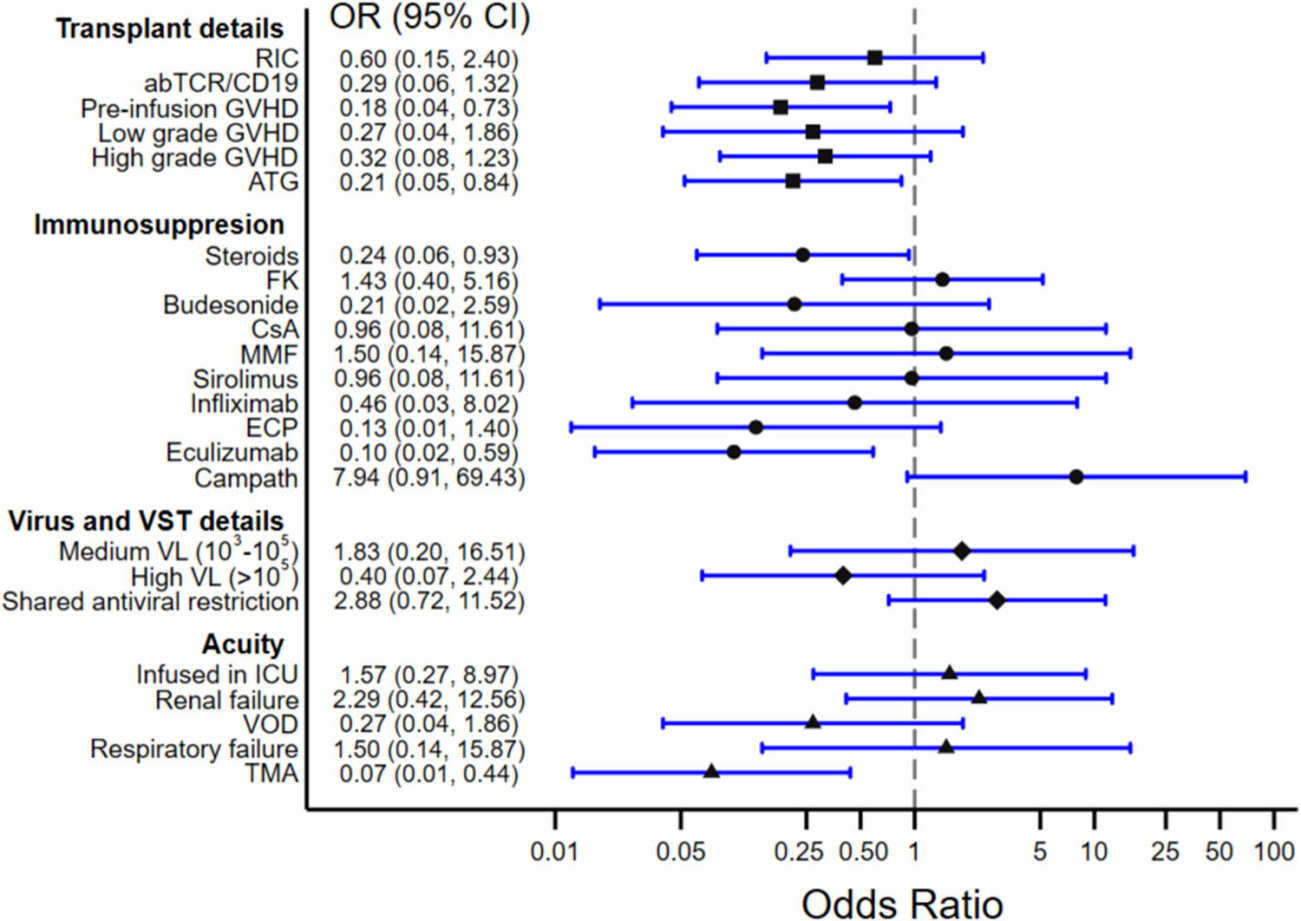
Impact of clinical factors on antiviral responses post VST therapy. Odds ratios (OR) in favor of antiviral responses (complete or partial response at day+28) based on a univariate logistic regression model are shown (*n* = 43). CI confidence interval, RIC reduced intensity conditioning, FK tacrolimus, CsA cyclosporin A, MMF mycophenolate mofetil, ECP extracorporeal photopheresis, VL viral load, VOD veno-occlusive disease, TMA transplant-associated thrombotic microangiopathy. Low grade GVHD: grades I-II; high grade GVHD: grade III-IV.

Logistic regression analysis of patient, transplant, and VST factors and their influence on the chances of antiviral responses showed several immunosuppressive agents as well as transplantation complications which worsened chances of antiviral responses to VST therapy (Fig. 4). Though high dose corticosteroids (≥0.5 mg/kg of prednisone equivalents at time of infusion) or anti-thymocyte globulin (ATG) treatment in the prior 4 weeks were exclusions, concurrent treatment with systemic corticosteroids at lower doses impacts chances of antiviral responses (odds ratio (OR) 0.24, 95% CI 0.06–0.93), as did prior use of ATG during conditioning (OR 0.21, 95% CI 0.05–0.84). Treatment with eculizumab either concurrently (*n* = 4) or in the prior 2 months (*n* = 5) also was significantly associated with a lower chance of antiviral response (OR 0.10, 95% CI 0.02–0.59). Previous history of GVHD also worsened chances of antiviral response (OR 0.18, 95% CI 0.04–0.73), as did history of transplant-associated thrombotic microangiopathy (OR 0.07, 95% CI 0.01–0.44). Notably, ongoing treatment with calcineurin inhibitors (*n* = 28), sirolimus (*n* = 4), mycophenolate (*n* = 4), and infliximab (*n* = 2) had no notable impact on responses. Initial viral load at time of infusion as well as several individual markers of patient acuity did not significantly impact response rates.

### Antiviral immune reconstitution data

**Fig. 5:**
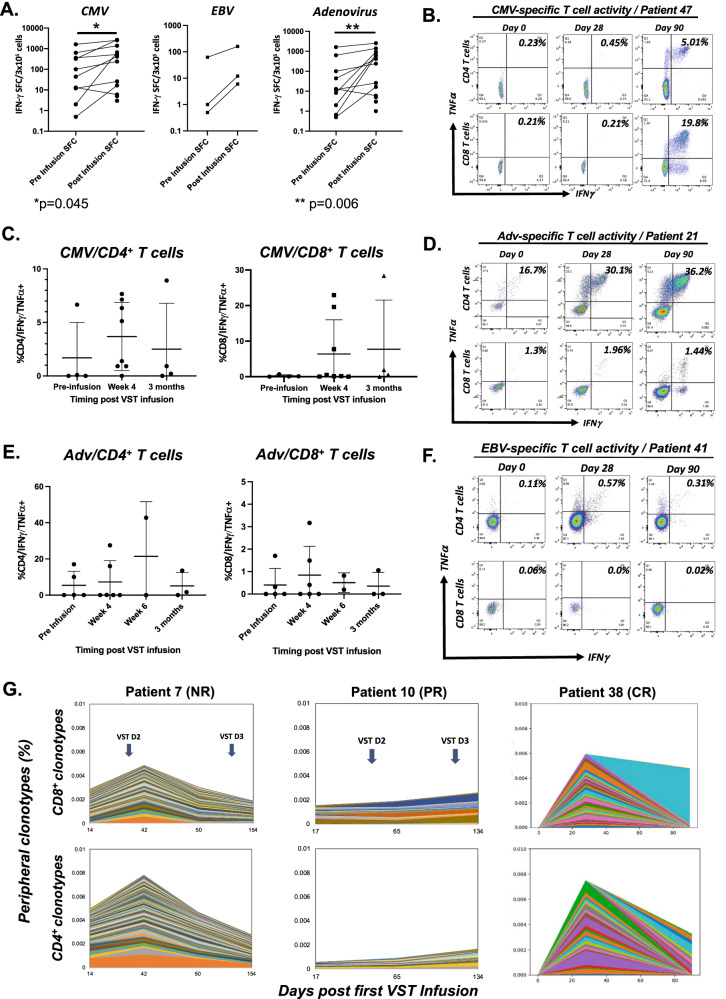
Immune reconstitution post VST therapy. **A** Trends in best response pre/post VST therapy against targeted viral antigens by IFN-γ Elispot were compared by 2-way ANOVA (CMV: *n* = 11; adenovirus: *n* = 15). SFC Spot forming colonies; *p = 0.045; **p = 0.006. **B**–**F** Virus-specific T cell reconstitution in recipients was evaluated by intracellular cytokine staining following a 7-day ex vivo expansion against targeted viral antigens. Longitudinal CD4^+^ and CD8+ responses against CMV (**B**, **C**, *n* = 10), adenovirus (**D**, **E**, *n* = 6), and EBV (**F**) were evaluated. Bar: mean; whiskers: standard deviation. **G** Peripheral frequencies of CMV-specific T cell receptor beta (TCR) clonotypes associated with the infused VST products were tracked relative to the first infusion in three recipients.

Prior to VST infusion, endogenous patient-derived T cell responses against targeted viruses were mixed, with 12 of 31 evaluated patients showing detectable antiviral responses by IFN-γ ELISpot and/or flow cytometry (Fig. 5). As expected, none of the patients on Arm B had detectable antiviral T cell activity at baseline. Following VST infusion, 20 (65%) patients showed an increase in the magnitude of antiviral T cell responses at a median of 7 days post VSTs, which was significant for CMV (*p* = 0.045) and adenovirus (*p* = 0.006) antigens by IFN-γ ELISpot (Fig. 5A, Supplementary Fig. 7A–C). Antiviral T cell responses showed polyfunctionality in vivo based on intracellular cytokine staining of post-infusion samples, with both CD4- and CD8-restricted responses (Fig. 5B–F), and were predominantly effector memory T cells (median 93.4%, range 8.4–99.4) at 1–3 months post-infusion, with smaller central memory (median 4.7%, range 0.6–81.7%) and TEMRA populations (median 0.1%, range 0–14.8%, Supplementary Fig. 8A, B). In patients treated for CMV, CD8 + T cell responses predominate in 4 of 8 patients evaluated by ICS at 1–3 months post infusion. Comparatively, CD4 responses are dominant for patients treated for adenovirus, with peaks at 1–3 months after VST infusion. Of the three evaluable patients who were treated pre-HSCT(Arm B), two had no noted antiviral activity post-infusion, whereas one (Patient 36) had transiently detectable anti-CMV activity at week 6 post-infusion #1, but no change in CMV viral load (Supplementary Fig. 5). Longitudinal trends in absolute lymphocyte counts (ALC) and CD8 + T cells differs between responders and non-responders, with a higher rate of increase in ALC over time in responding patients versus non-responding patients (positive interaction between being responder and time with *p* < 0.001 for both ALC and CD8 count), Table 4 and Supplementary Fig. 9A–C). In Patients 43 and 46 who developed neurologic disease, we could find no evidence of antiviral T cell expansion by ELISpot and/or TCR sequencing. Patient 46 had adenovirus-specific TCR clonotypes detectable in the CSF on day +45, but with no improvement in adenoviral load post-infusion. Comparably, patient 43 showed reduction in adenoviral viral load, but without detection of VST engraftment by ELISpot or TCR sequencing.

**Table 4:** Mixed model regression analysis of lymphocyte trends over time

Outcome	Predictor	Mean change in log(outcome)		
		Estimate	95% CI	*p*-value
ALC	CR/PR vs.NR at time of infusion	−0.287	−0.864, 0.289	0.3
	Weeks after infusion for NR	−0.042	−0.081, −0.003	**0.034**
	Weeks after infusion for CR/PR	0.042	0.019, 0.064	**<0.001**
CD4	CR/PR vs.NR at time of infusion	0.465	−0.678, 1.607	0.4
CD8	CR/PR vs.NR at time of infusion	0.315	−0.806, 1.436	0.6
	Weeks after infusion for NR	−0.01	−0.083, 0.062	0.8
	Weeks after infusion for CR/PR	0.07	0.032, 0.107	**<0.001**

### VSTs persistence is transient in peripheral blood

Following treatment, persistence of infused VSTs was evaluated by both flow cytometry and TCR deep sequencing. TCR sequencing was performed on the virus-specific components of VST products following sorting by IFN-γ capture assay. as well as sorted CD4^+^ and CD8^+^ T cells from longitudinal patient blood samples. T cell clonotypes corresponding to the infused VST products are present in 6 of 11 patients at 2–4 weeks post infusion (Fig. 5G), and remain detectable for up to 90 days. Four of 8 patients who had antiviral responses had antiviral clonotypes detectable in both the CD4+ and CD8 + T cell fractions after infusion. In order to track CMV-specific clonotypes from either the VST product or graft more broadly, we also evaluated public CMC-specific clonotypes in the recipients. Four of 6 responding patients treated for CMV had >20 public CMV-specific clonotypes detectable following infusion (Supplementary Table 3). Public clonotypes previously associated with immunodominant CMV epitopes are detected in six patients following VST infusion (Supplementary Data 1). Most of these clonotypes were low frequency, with exception of a clonotype targeting B07:02-restricted epitope TPRVTGGGAM, which was detectable at 3.34% at day 15 post infusion in Patient 25. Patient 7 had detectable CMV-specific clonotypes from the product as well as public CMV-specific clonotypes in peripheral blood, but failed to achieve resolution of CMV retinitis during the study period. Flow cytometry evaluation for infused VST products by staining of disparate HLA antigens in expanded T cells from 15 patients showed that the vast majority of antigen specific T cells detected in peripheral blood between months 1–3 were derived from the recipient or BMT donor (median 99.8%, range 84–100%), with minimal appreciable T cells from the VST donor observed in peripheral blood at subsequent times after infusion (Supplementary Fig. 10). No correlation was observed between viral loads and frequencies of CD4 or CD8-associated virus-specific clonotypes (Supplementary Fig. 11A–D), nor were there detectable differences in clonotype diversity between responders and non-responders (Supplementary Fig. 11E).

## Discussion

Despite advances in antiviral therapies, viral infections remain an appreciable risk for immunocompromised pediatric patients, including HSCT recipients and patients with IEI^5,18,47–51^. Single center studies have described transplant-related mortality rates as high as 50% in pediatric patients with CMV reactivation post HSCT^52^. Viral infections are especially dangerous and common in patients with SCID and related forms of IEI, in whom DNA viruses and respiratory viruses are among the most common infections identified^53^. In the IEI population, viral infections pre-HSCT are rarely cleared and correlate with poorer transplantation survival rates^54,55^. Adoptive T cell therapy has been utilized in multiple, predominantly single center, studies over the past two decades with good tolerance and anti-viral activity^23,56–62^. However, use in pediatrics has been scattered, and many studies limited inclusion of critically ill patients^25,44,58,63^. Here, we demonstrated the feasibility of third party VST therapy in immunocompromised pediatric patients, including critically ill patients, in a multicenter consortium setting. Prior reports on similar patient cohorts with drug-refractory viral infections have shown poor survival rates, particularly in the setting of GVHD and other co-morbidities^4,7,8,48^. Suitable products were identified for 97% of patients, and 51 patients were treated using 18 VST products, demonstrating that a relatively small VST bank (40 products in this case) can facilitate treatment of a larger number of patients. Though high acuity patients had poorer overall survival, 6 of 20 high acuity patients had antiviral responses and survived, which is higher than would be expected in this population^64,65^. In logistic regression analysis, several clinical factors were noted to be associated with responses, including systemic corticosteroids, prior use of ATG, and history of GVHD. As VSTs are susceptible to immunosuppressive agents, these risk factors are not surprising. In comparison, prior alemtuzumab pre-HSCT did not impact antiviral responses, in spite of similar timing of VST infusions post-HSCT (mean day +120 for ATG versus +108 for alemtuzumab). This may be reflective of the differing half-lives and biological effects of ATG and alemtuzumab^66,67^. History of TA-TMA as well as use of eculizumab treatment significantly worsened chances of antiviral responses. As nearly all patients with TMA were receiving eculizumab (10/11), it is unclear whether this effect stems from the underlying process or C5a blockade. Crosstalk between complement pathways and adaptive immunity have been described, and C5a has been shown to impact activity of antigen presenting cells as well as survival, differentiation, and activity of CD4 + T cells^68–72^. Of note, Rubinstein et al. did not note an adverse impact of TMA or eculizumab treatment on clinical response to VST therapy in 13 patients, and demonstrated persistent interferon-γ release during complement inhibition^73^. Viral load, patient acuity, and degree of HLA match did not appear to impact chances of responses, though confirmation of an HLA restriction shared between the VST donor and recipient trended toward improved responses.

Third party VSTs were generally well tolerated, though rare but significant toxicities were observed in 4 patients. Cytokine release syndrome (CRS) was diagnosed in one patient with disseminated adenovirus. Though rare, CRS has been described previously after VST therapy^74^, and treatment with tocilizumab and corticosteroids is generally effective, though it may also suppress VST antiviral activity. Secondary graft rejection was observed in one child with SCID in association with VST expansion (detailed in NCOMMS-22-38184A), which has never been described previously after VST therapy, and resembled transfusion-associated GVHD. Extensive investigation of this event showed a highly narrow repertoire of T cells in the recipient, none of which appeared to be CMV-specific. The patient in this case was unique (RAG1 SCID post αβTCR/CD19 depleted MMRD HSCT), with no similar patients reported to have received VST therapy. The extreme lymphopenia of the patient, as well as donor factors including age and parity may have contributed to this event, and speaks to the need for further studies to evaluate both recipient and donor factors that may influence VST safety. Finally, two patients developed neurologic injury after VST infusion for disseminated adenoviral infection. Immune effector associated neurologic syndrome (ICANS) is well-described after CD19 chimeric antigen receptor (CAR) T cell therapy^75^, but never described previously after VST therapy. Both cases had elevation of sILR2, but no evidence of antiviral T cell expansion. In both cases, immunosuppression with tocilizumab and steroids failed to result in clinical improvement, suggesting the cause of the neurological dysfunction may have been due to progressive viral disease. Another possible contributing factor was TA-TMA, a known possible cause of neurological dysfunction^76–78^, which had been previously treated in Patient 46, and could not be disproven in Patient 43.

Antiviral T cell activity was detected in the majority of responding patients, with polyfunctional T cells targeting viral antigens observed at a median of 7 days post infusion. Notably, 12 of 31 patients had detectable T cell activity at baseline despite persistent viral infection, though most had improvement in the magnitude of T cell activity at a median of 7 days post infusion. This suggests that suboptimal T cell function, rather than simply absence, may occur in patients with refractory viral infections post-HSCT, as has been described previously^79^. TCR sequencing showed persistence of infused cells in a subset of patients at day 28 post-infusion, but responses occurred despite lack of detectable clonotypes in 4 of 8 evaluated patients. Additionally, virus-specific T cells in peripheral blood were predominantly derived from HSCT donor grafts at months 1–3 based on staining of discordant HLA antigens. This suggests that engraftment of third party VST is likely transient, or could be primarily focused to sites of infection. The noted improvement in antiviral T cell function in vivo suggests that VSTs may support immune reconstitution from the HSCT graft. Responders were noted to have significant uptrending in ALC over time in comparison with non-responders in spite of comparable baseline ALC (median 0.44 x 10^3^/mcl in both populations), which supports the premise that success of third-party VST therapy is dependent on overall immune reconstitution. This is in keeping with prior studies that noted higher baseline CD4 counts in patients who responded to CMV-specific VST therapy^80^. This may also explain the distal impact of ATG on antiviral responses, given the impact of serotherapy on T cell reconstitution kinetics.

Four patients with IEI received VST therapy prior to HSCT without improvement. In all cases, no engraftment nor antiviral activity was noted after infusion. A small number of case reports have described use of VSTs in IEI patients prior to transplant^81,82^. Antiviral efficacy has only been seen when VSTs were closely followed by HSCT. Based on the transient nature of third-party VSTs in vivo, it is probable that VST infusion alone inadequate to restore antiviral immunity without a stem cell allograft to facilitate complete immune reconstitution. Further study is necessary to improve treatment outcomes in IEI patients with refractory viral infections prior to HSCT.

Limitations of this study included the sample size, heterogeneity of diagnoses and viral infections, and variation in therapeutic management across institutions. Restraints in number and volume of blood samples based on patient ages also limited the breadth of correlative studies that could be performed. Given the limited data on recipient and donor serologies, we also were unable to distinguish primary CMV/EBV infections versus secondary reactivations in the post-HSCT setting, which could have important implications for best methods of HLA matching between the recipient, HSCT donor, and VST donor and warrants further study.

In summary, we showed in a consortium led pediatric phase II study that partially HLA matched VST therapy is feasible and efficacious in the pediatric HSCT setting and surpassed the primary clinical efficacy endpoint of 52%, with 85% overall survival in responders. Though generally well tolerated, it is essential to monitor for rare adverse effects including CRS after third party VST therapy, particularly in patients with high viral burden. Secondary graft rejection is exceedingly rare but also could be a potential risk in the setting of severe lymphocytopenia, such as SCID and similar IEI patients undergoing T cell-depleted transplantation. GVHD and need for immunosuppressive therapies remain major hurdles for successful VST therapy for viral infections. Our study suggests that a yet to be fully defined minimal level of immune reconstitution and the potential for stimulation of recovering marrow may be important to the success of VST therapy. Future studies may elucidate additional risk factors for adverse events, and genetically modified VSTs may enable treatment in the presence of immunosuppressive therapies, thus enabling earlier and more efficacious antiviral therapy.

## Methods

### Study design and patients

The study was a multicenter, prospective, two-arm phase II trial. Recipients of allogeneic hematopoietic stem cell transplantation (Arm A), or patients with inborn errors of immunity (IEI) who had not undergone HSCT (Arm B) with viral infections with CMV, EBV, and/or adenovirus that were refractory to treatment were eligible for enrollment. Refractory infections were defined as less than 1-log-fold reduction in viral load after at least 2 weeks on standard antiviral therapy, persistence of visceral symptoms attributed to viral infection despite antiviral therapy. Patients with EBV-lymphoproliferative disease with <50% decrease in disease burden by imaging studies (based on RECIST criteria) following rituximab therapy were also eligible for enrollment. Patients who were unable to tolerate antiviral therapies due to toxicities or organ dysfunction were also eligible for enrollment. Exclusion criteria included active graft versus host disease (grade 3 or higher), concurrent systemic corticosteroid therapy (>0.5 mg/kg/day prednisone equivalents), recent receipt of biologic therapies targeting T cells, current immune checkpoint inhibitor therapy, uncontrolled infections aside from the targeted viruses, or donor lymphocyte infusion or other experimental cellular therapies in the previous 28 days (full protocol provided in the supplementary notes).

Patient enrollment was stratified by primary targeted virus, with CMV and adenovirus being the primary stratum. Goal enrollment on each primary stratum was at least 20 patients. Based on historical data, we assumed a clinical response rate of <20% without VST treatment. In each of the two primary strata, with Type I error no greater than 5% and with a sample size of 20, there was at least 90% power to detect an improvement in antiviral response rate to 52%. Stopping guidelines included occurrence of dose limiting toxicity in ≥25% of patients, and was defined as occurrence of high grade graft-versus-host disease (>grade 2), or grades 3–5 infusion-related adverse events or grades 4–5 non-hematological adverse events based on the NCI Common Terminology Criteria for Adverse Events (CTCAE), Version 4.03.

This study was centrally approved at the Institutional Review Board of the Children’s Hospital of Los Angeles (Los Angeles CA, USA) as well as the institutional review boards of each participating sites prior to patient recruitment (full institution list in Supplementary Table 4). Written consent and assent (as applicable) were obtained from all participants and legal guardians for participants under 18 years of age, in accordance with the Declaration of Helsinki. This study was registered on Clinicaltrials.gov as NCT03475212. Full list of inclusion/exclusion criteria can be found in the study protocol (including in Supplementary Notes).

### Third-party Donors and Manufacturing of virus-specific T cell products

Thirty-four healthy donors were enrolled for generation of VST products for this study. Donors underwent standard risk screening and infectious disease testing as well as supplemental suitability screening for third-party donors for immune effector cell therapy (see study protocol in supplementary notes). Generation of virus-specific T cells from PBMCs was performed in the Children’s National Hospital Good Manufacturing Practices facility using a rapid expansion protocol as previously described^24^. Briefly, PBMCs were pulsed with a mix of overlapping pools of 15-mer peptides encompassing six viral antigens (CMV pp65/IE1, EBV LMP2/EBNA1, adenovirus Hexon/Penton) at 100 ng/peptide/ul for 30–60 min at 37 °C. Peptide libraries of 15-mers at GXP grade were utilized (JPT, Berlin, DE). After incubation, cells were resuspended with IL-4 (400 U/ml; R&D Systems, Cat#204-GMP-050, Minneapolis, MN) and IL-7 (10 ng/ml; R&D Systems, Cat#207-GMP-025) in CTL media consisting of 45% Advanced RPMI (GE Healthcare, Logan, UT), 45% Click’s medium (Irvine Scientific, Santa Ana, CA), 10% fetal bovine serum, and supplemented with 2 mM GlutaMax (Gibco, Grand Island, NY), and expanded in G-Rex-10 bioreactors (Wilson Wolf, Cat#80040 S, New Brighton, MN). Cytokines were replenished on day 7. On day 10, cells were harvested and underwent clinical release testing for identify, sterility, phenotype, and function. VSTs were evaluated for antiviral activity via intracellular cytokine staining, IFN-γ ELISpot, and MHC-pentamer staining (where applicable). Mapping of viral epitope was performed using matrices of 15-mer peptides in IFN-γ ELISpot assays, and antiviral MHC restrictions were determined based on in silico analysis (IEDB MHC I/II binding) and use of mixed IFN-γ ELISpot (Enzyme-linked immunospot) assays with the product and partially HLA matched, peptide-pulsed PHA blasts.

### VST matching criteria

Enrolled patients were screened for suitable banked VST products based on their HLA as well as the HLA of their HSCT donor (if mismatched). Criteria for VST matching was based primarily on identification of products with one of more shared antiviral HLA restrictions with the recipient and HSCT donor, overall antiviral specificity of the VST product, and finally based on overall HLA match. High-resolution HLA matches were required for class I alleles, whereas low resolution matches were permitted for class II alleles. For patients with known anti-HLA antibodies, avoidance of sensitized HLA alleles was attempted.

### Treatment, monitoring, and follow-up

Patients received a fixed dose of 2 × 10^7^ VSTs/m^2^ body surface area at their local treating institution. The primary study outcome was antiviral efficacy based on viral load. Viral PCRs were performed in blood and other infected body fluid at local centers at defined intervals (see study protocol in supplementary notes). Antiviral responses were defined at 30 days after each VST infusion as follows: complete responses were defined as resolution of viremia and any symptoms/visceral disease attributable to viral infection; partial responses were defined as a sustained ≥1-log reduction in viral load or >50% decrease in radiographic disease for EBV-PTLD; non-responders were those with viral load changes insufficient to qualify as partial response, or those with progression in viral disease. Patients who had a partial response or no response and no treatment-related toxicities were eligible to receive up to 3 additional doses from day 30 after the initial infusion and at 2 weekly intervals thereafter. Patients were monitored for 1 year following their final VST infusion. Dose-limiting toxicities were defined as high-grade acute GvHD (grades III-IV), grades 3–5 infusion-related adverse events or grades 4–5 non-hematological adverse events possibly related to the T cell product within 30 days of each VST dose and that are not due to pre-existing infection, malignancy or co-morbidities, as defined by the NCI Common Terminology Criteria for Adverse Events (CTCAE, Version 4.03). Antiviral immunologic monitoring was performed from peripheral blood weekly through day 28 and at days 45 and 90.

### IFN-γ enzyme-linked immunospot (ELISpot) assay

Antigen specificity of T-cells was measured by IFN-γ ELISpot (Millipore, Cat#MSHAS4510, Burlington, MA). T-cells were plated at 1 × 10^5^/well with no peptide, actin (JPT, Cat#PM-ACTS), or each of the individual CMV pp65/IE1, EBV LMP2/EBNA1, adenovirus Hexon/Penton peptide libraries(200 ng/peptide/well). Sample were tested in triplicate whenever possible based on cell availability. Plates were sent for IFN-γ spots forming cells (SFC) counting (Zellnet Consulting, Fort Lee, NJ).

### Flow cytometry

VSTs were stained with fluorophore-conjugated antibodies against CD4, CD8, TCRαβ, TCRγδ, CD16, CD19, and CD56 (Miltenyi Biotec, Bergisch Gladbach, Germany; BioLegend). All samples were acquired on a CytoFLEX cytometer (Beckman Coulter, Brea, CA). Intracellular cytokine staining was performed as follows: 1 × 10^6^ VSTs were plated in a 96-well plate and stimulated with pooled pepmixes or individual peptides (200 ng/peptide/well) or actin (control) in the presence of brefeldin A (Golgiplug; BD Biosciences, Cat#BD555029, San Jose, CA) and CD28/CD49d (BD Biosciences, Cat#347690) for 6 h. T-cells were fixed, permeabilized with Cytofix/Cytoperm solution (BD Biosciences, Cat#554714) and stained with IFN-γ and TNF-α antibodies (Miltenyi Biotec). Concurrent sample replicates were performed when possible, based on availability of cells. Data was analyzed with FlowJo X (FlowJo LLC, Ashland, OR). Antibody panels and dilution details are listed in Supplementary Tables 5–9. Gating strategy is included in Supplementary Fig. 12A, B.

### T cell receptor sequencing

T cell receptor sequencing was performed on sorted T cells or PBMCs utilizing either an RNA-based amplification protocol as previously described^83^, or using the Immunoseq platform (Adaptive Biotech, Seattle WA), depending sample availability. Samples for each individual patient was performed on consistent platforms. Briefly, for RNA-based sequencing, RNA was extracted using RNAZol, and cDNA produced using Oligo dT primers and Superscript II RT kit (Invitrogen). cDNA was cleaned using the Agencourt AMPure XP kit (Beckman Coulter, Cat#NC9933872, Brea, Calif). The CDR3 region of TRB was dual amplified by using customized primers (Supplementary Table 10) with the KAPA Real Time Library Amplification kit (Kapa Biosystems, Cat#50-196-5271, Woburn, Mass). The PCR product was cleaned by using the Agencourt AMPure XP kit, according to the protocol. The libraries were quantified with the Kapa Library quantification kit (Roche, Cat# 07960140001, Basel SW). Libraries were pooled and sequenced on an Illumina MiSeq with a minimum coverage of 10 reads per cell. TCR clonotype calling and analysis was performed using MiXCR version 4.1.0. Public clonotypes were identified using VDJMatch version 1.3.1.

### Multiplex cytokine assay

Plasma samples were evaluated using the Bio-plex Pro Human 17-plex Cytokine Assay kit (Bio-Rad, Cat#M5000031YV, Hercules, CA, USA), and read on a MAGPIX system (Luminex, Austin, TX, USA).

### Statistical analysis

Clinical responses were categorized based on viral load trends and viral symptoms as complete (CR), partial (PR), or non-responders (NR) per the study design Patients lacking data for clinical response classification were considered not evaluable (NE), and were not considered in the analysis. Clinical response classifications and timing were judged by a panel of blinded investigators, with at least 3 investigators evaluating each patient’s data to ensure uniformity of classifications. For analyzing association between the dichotomous response variable (CR/PR & NR) with categorical clinical factors, a univariate logistic regression model was fit to each predictor. The odds ratio in favor of a response and its 95% confidence interval were reported in a forest plot (Fig. 4).

Overall survival was defined by the time of the first infusion to time of death (of any cause) or last follow-up (as a censoring time). Kaplan–Meier estimates of the survival curves were calculated with Greenwood’s methods for the 95% confidence intervals. The survival curves were compared with the log-rank test.

Patients had ALC, CD4 & CD8 from baseline to 12 weeks with measurements being 2 to 4 weeks apart. The logarithm of these values was fit to a mixed effects model with time point (in weeks), response (NR being the reference group) and their interaction as fixed-effect predictors and intercept and time as random-effect predictors to account for correlations between measurements of the same patient. The best model was selected as having the smallest AIC (Akaike Information Criterion), an estimator of prediction error (Table 4). The forest plot as well as other graphical presentations were constructed with Stata version 17, while statistical analyses including the mixed models were performed using R versions 4.2.1. The statistical analysis plan for this trial is included in Supplementary Notes. CONSORT checklists are included as Supplementary Tables 12–13.

Shannon diversity calculations were performed in Python using the following calculation (Shannon diversity = *−Σp*_*i*_ *ln p*_*i*_*)*. Analysis of immune reconstitution data, including 2-way ANOVA for flow cytometry and ELISpot results, and logarithmic correlation between CD4 and CD8 clonotype frequencies and viral loads was performed in Graphpad Prism, ver. 9.3.1.

### Reporting summary

Further information on research design is available in the Nature Portfolio Reporting Summary linked to this article.

### Supplementary information

Supplementary Information

Peer Review File

Description of Additional Supplementary Files

Supplementary Data 1

Reporting Summary

### Source data

Source Data

## Data Availability

All data are included in the Supplementary Information files. The raw numbers for charts and graphs, including de-identified patient data, are available in the Source Data file. The flow cytometry and TCR sequencing datasets are available on Zenodo under the following link [https://zenodo.org/records/10562383]. The study protocol and statistical analysis plan are included in the Supplementary information. Source data are provided with this paper.
