# Supplementary material for: Antiviral cellular therapy for enhancing T-cell reconstitution before or after hematopoietic stem cell transplantation (ACES): a two-arm, open label phase II interventional trial of pediatric patients with risk factor assessment
Source: Nat Commun. 2024 Apr 18;15:3258. doi: 10.1038/s41467-024-47057-2 (PMC11026387; doi:10.1038/s41467-024-47057-2)
Supplement: Supplementary file 1 — Supplementary Information [file 41467_2024_47057_MOESM1_ESM.pdf]

Supplementary Material

Antiviral Cellular Therapy for Enhancing T-cell Reconstitution Before or After Hematopoietic Stem Cell Transplantation (ACES): a two-arm, open label phase II interventional trial of pediatric patients with risk factor assessment

Michael D. Keller, *et al.*

Corresponding author:

Michael A. Pulsipher, MD

2000 Circle of Hope Drive Rm 3515

Huntsman Cancer Institute, Spencer Fox Eccles School of Medicine at the University of Utah, Salt Lake City, UT 84112

Email michael.pulsipher@hci.utah.edu

Supplementary Figures 1-12
Supplementary Tables 1-12
Supplementary Notes
    Note 1: Study Protocol
    Note 2: Statistical Analysis Plan

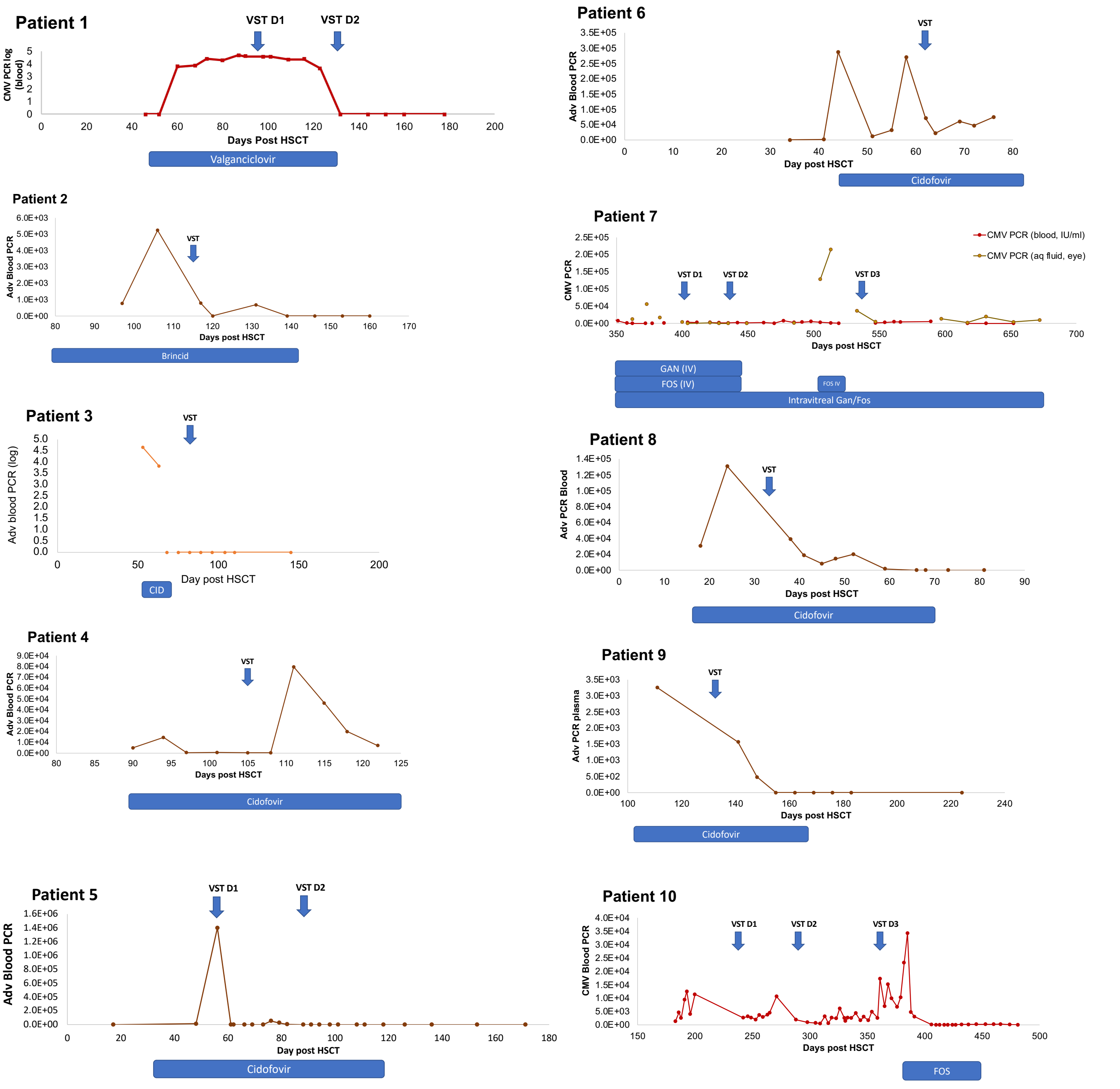

Supplementary Figure 1: Longitudinal viral trends in Patient 1-10. Arrows: virus-specific T cell (VST) infusions. FOS: foscarnet. CID: cidofovir. BRINCID: brincidofovir. GAN: ganciclovir, VALGAN: valganciclovir. MP: methylprednisolone. AMS: altered mental status. Siltux: siltuximab.

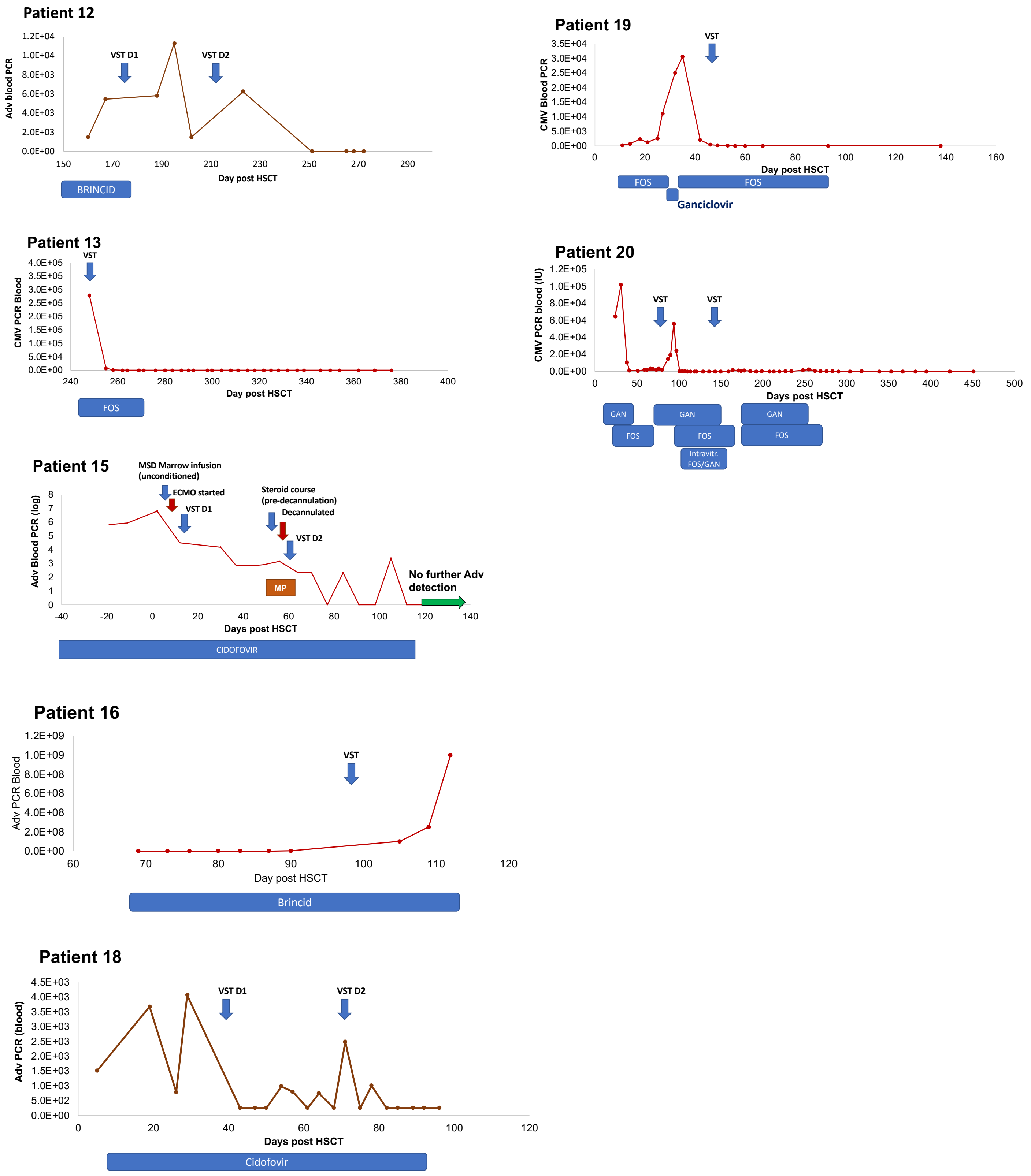

Supplementary Figure 2: Longitudinal viral trends in Patient 12-20. Arrows: virus-specific T cell (VST) infusions. FOS: foscarnet. CID: cidofovir. BRINCID: brincidofovir. GAN: ganciclovir, VALGAN: valganciclovir. MP: methylprednisolone. AMS: altered mental status. Siltux: siltuximab.

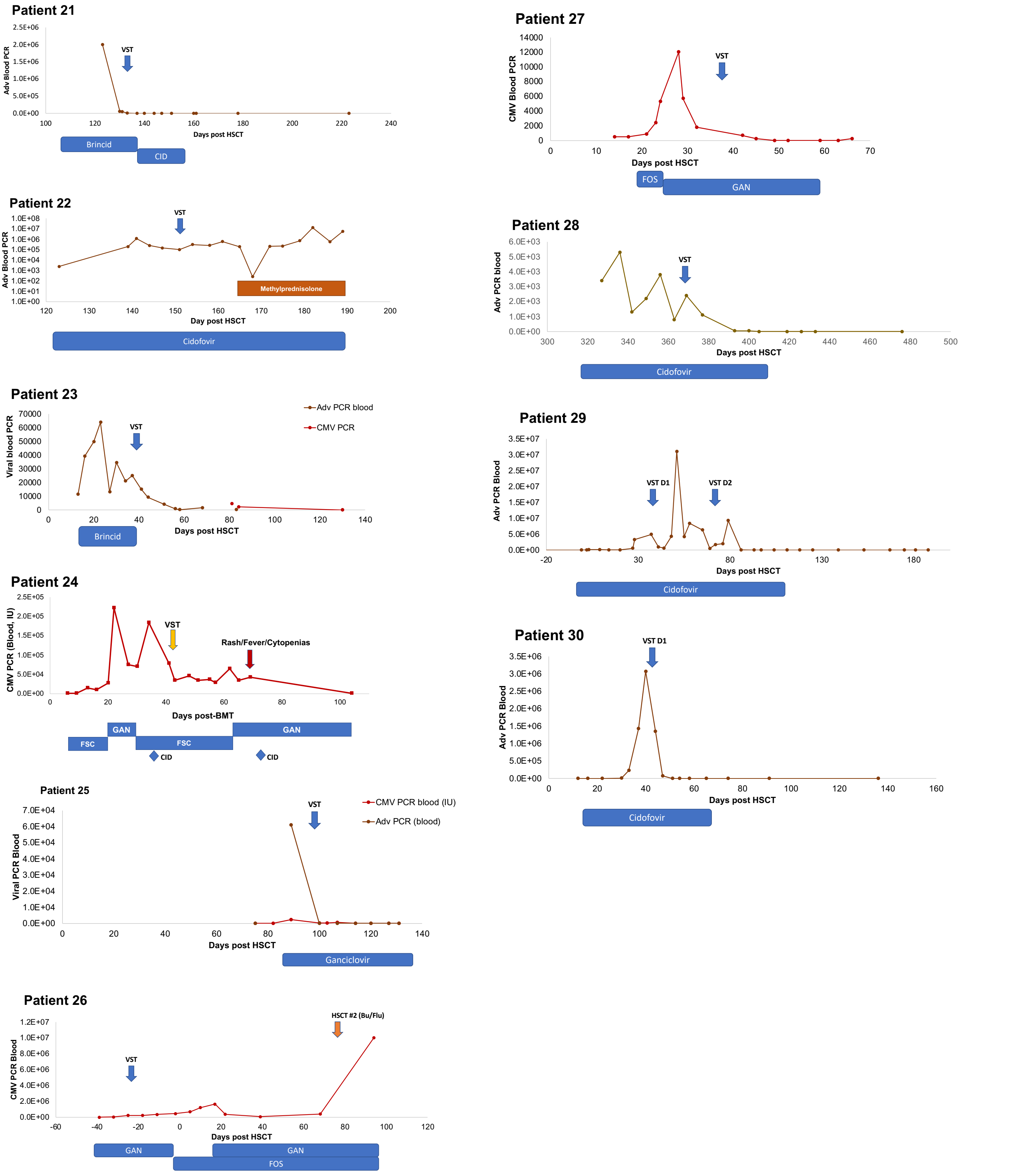

Supplementary Figure 3: Longitudinal viral trends in Patient 21-30. Arrows: virus-specific T cell (VST) infusions. FOS: foscarnet. CID: cidofovir. BRINCID: brincidofovir. GAN: ganciclovir, VALGAN: valganciclovir. MP: methylprednisolone. AMS: altered mental status. Siltux: siltuximab.

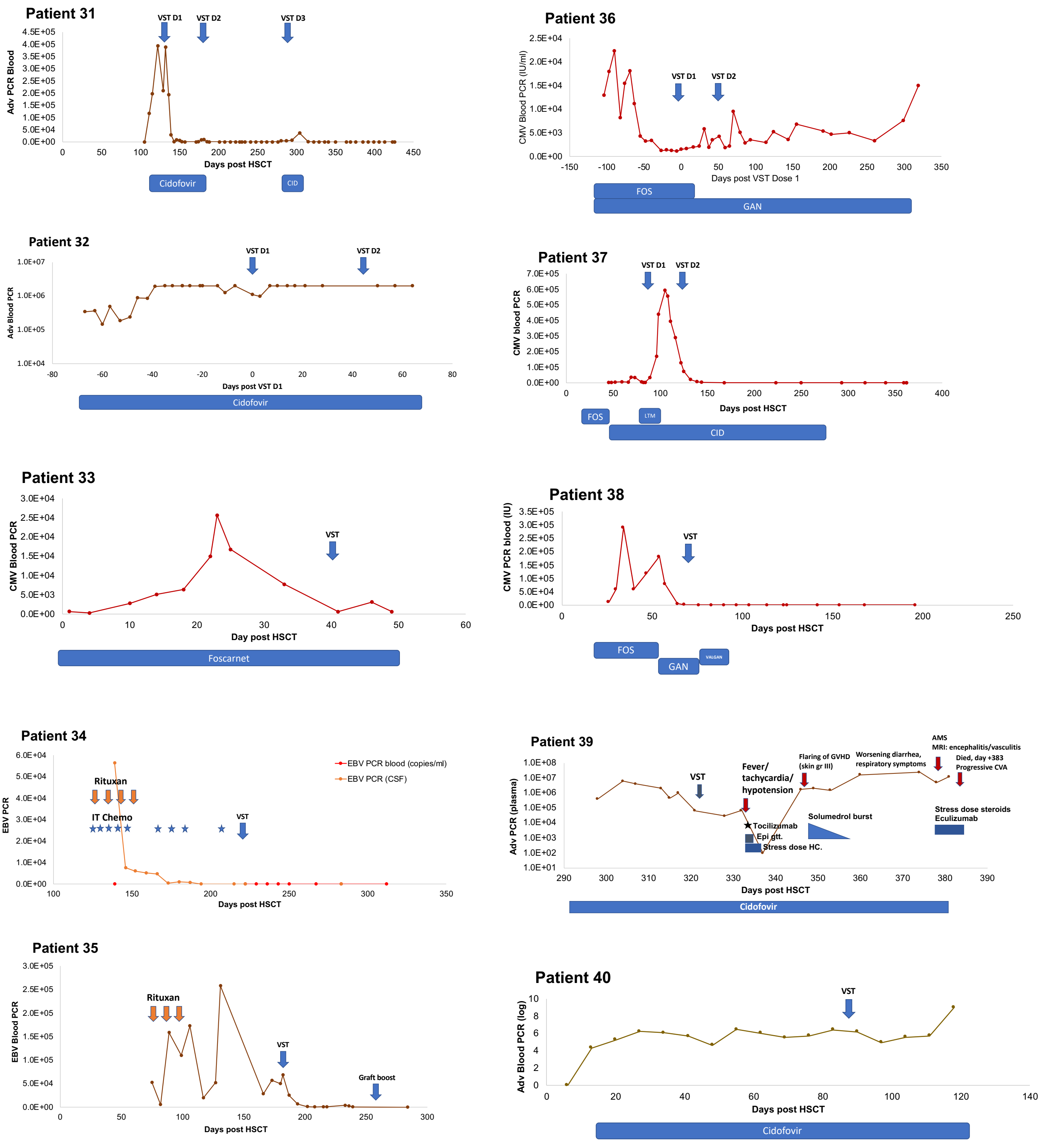

Supplementary Figure 4: Longitudinal viral trends in Patient 31-40. Arrows: virus-specific T cell (VST) infusions. FOS: foscarnet. CID: cidofovir. BRINCID: brincidofovir. GAN: ganciclovir, VALGAN: valganciclovir. MP: methylprednisolone. AMS: altered mental status. Siltux: siltuximab.

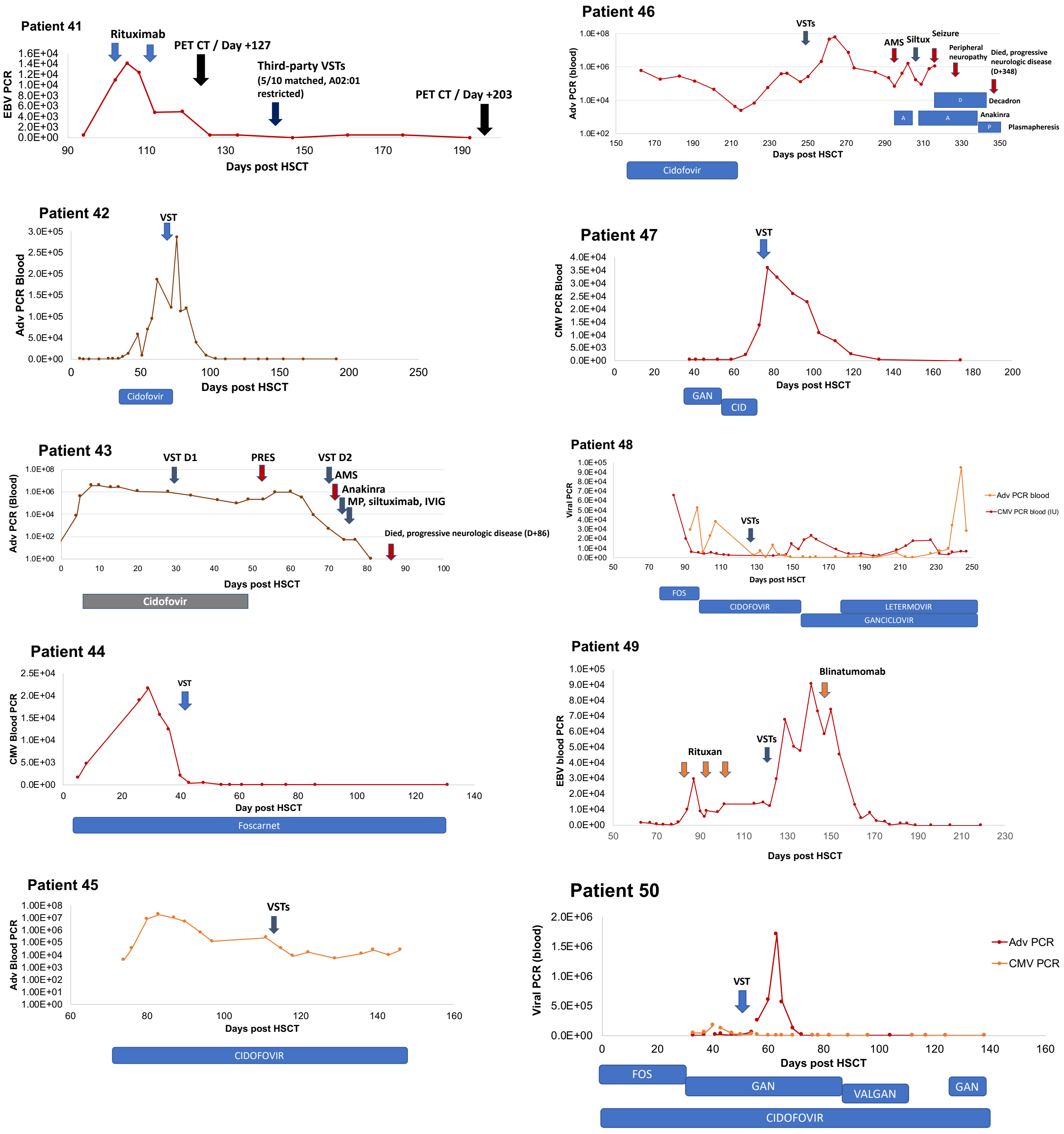

Supplementary Figure 5: Longitudinal viral trends in Patients 41-50. Arrows: virus-specific T cell (VST) infusions. FOS: foscarnet. CID: cidofovir. BRINCID: brincidofovir. GAN: ganciclovir, VALGAN: valganciclovir. MP: methylprednisolone. AMS: altered mental status. Siltux: siltuximab.

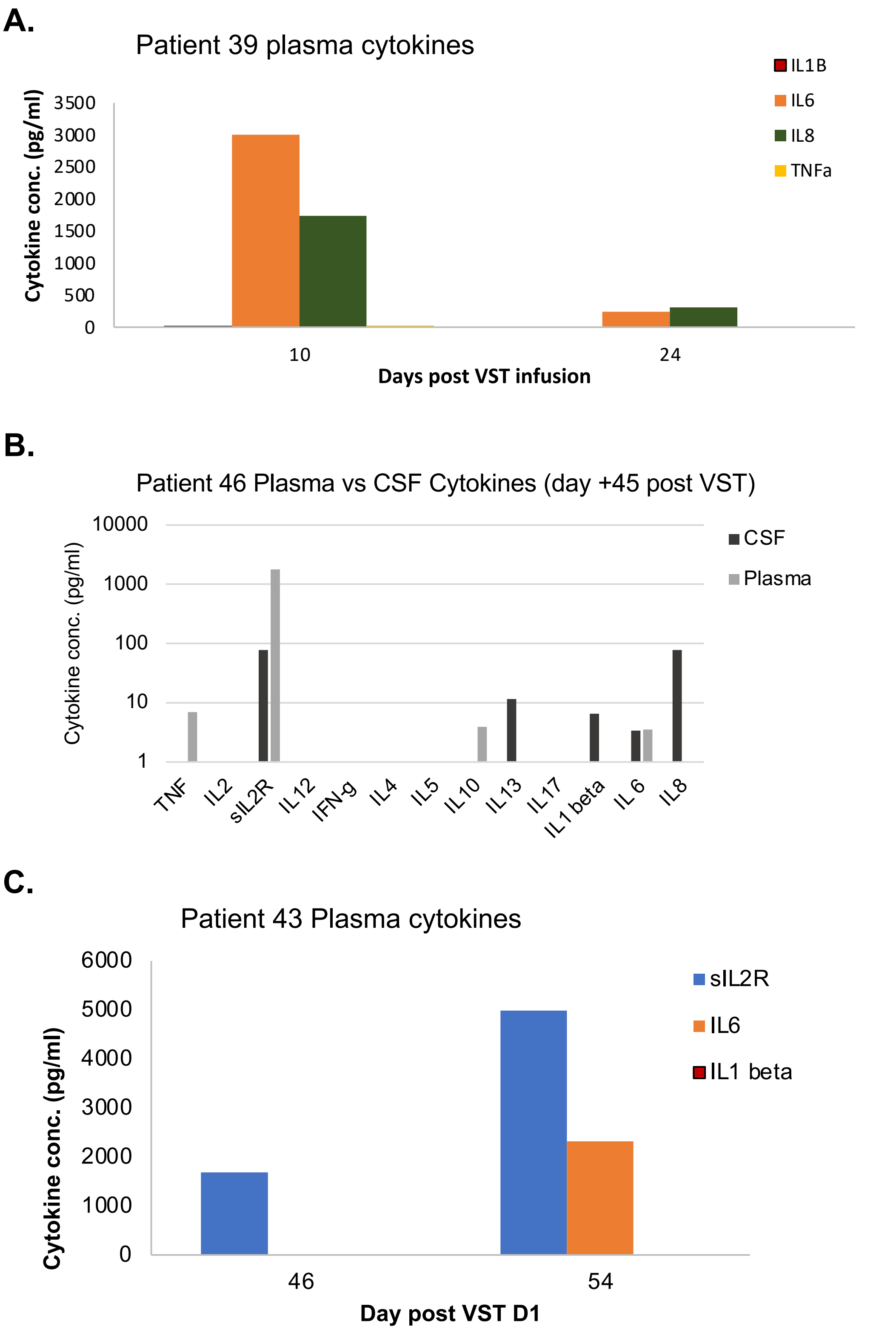

Supplementary Figure 6: Cytokine studies in patients with adverse events. A. Plasma cytokines in Patient 39 post VST infusion. B. Plasma vs cerebrospinal fluid (CSF) cytokines in Patient 46 at day +45 post VST infusion. C. Plasma cytokines over time in Patient 43.

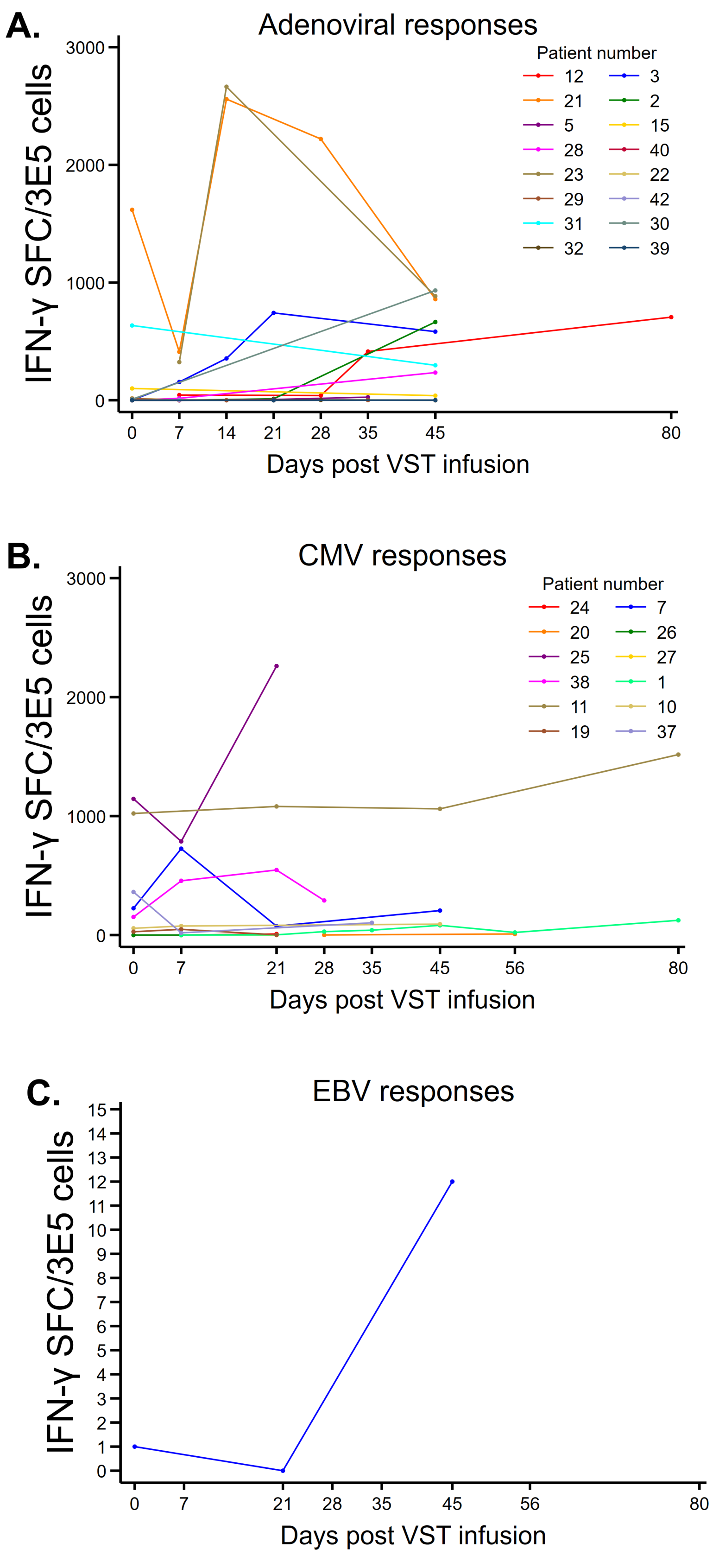

Supplementary Figure 7: Longitudinal Antiviral responses by IFN-γ ELISpot by primary targeted virus. A. Adenoviral responses (Hexon + Penton, n=16). B. CMV responses (pp65+IE1, n=12). C. EBV responses (LMP2 + EBNA1, n=1). SFC: Spot forming colonies.

A.

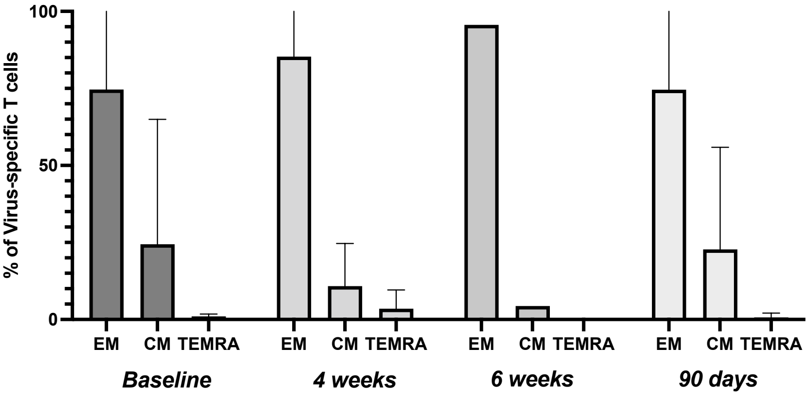

B. *Patient 21, Week 6*

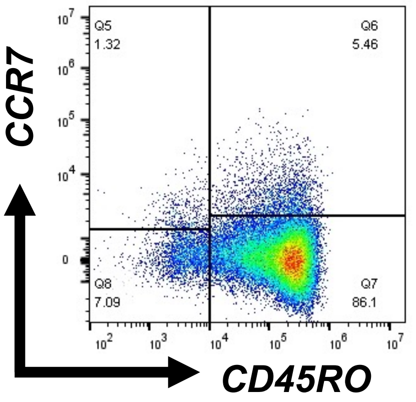

Supplementary Figure 8: Differentiation status of dominant antiviral T cell populations in vivo. A. T cell memory populations over time (n=9). EM: effector memory, CM: central memory, TEMRA: T effector RA. Box: mean; whiskers: standard deviation. B. Example plot of virus-specific T cell populations (based on IFNγ+/TNFα+ gating after viral peptide restimulation) at week 6.

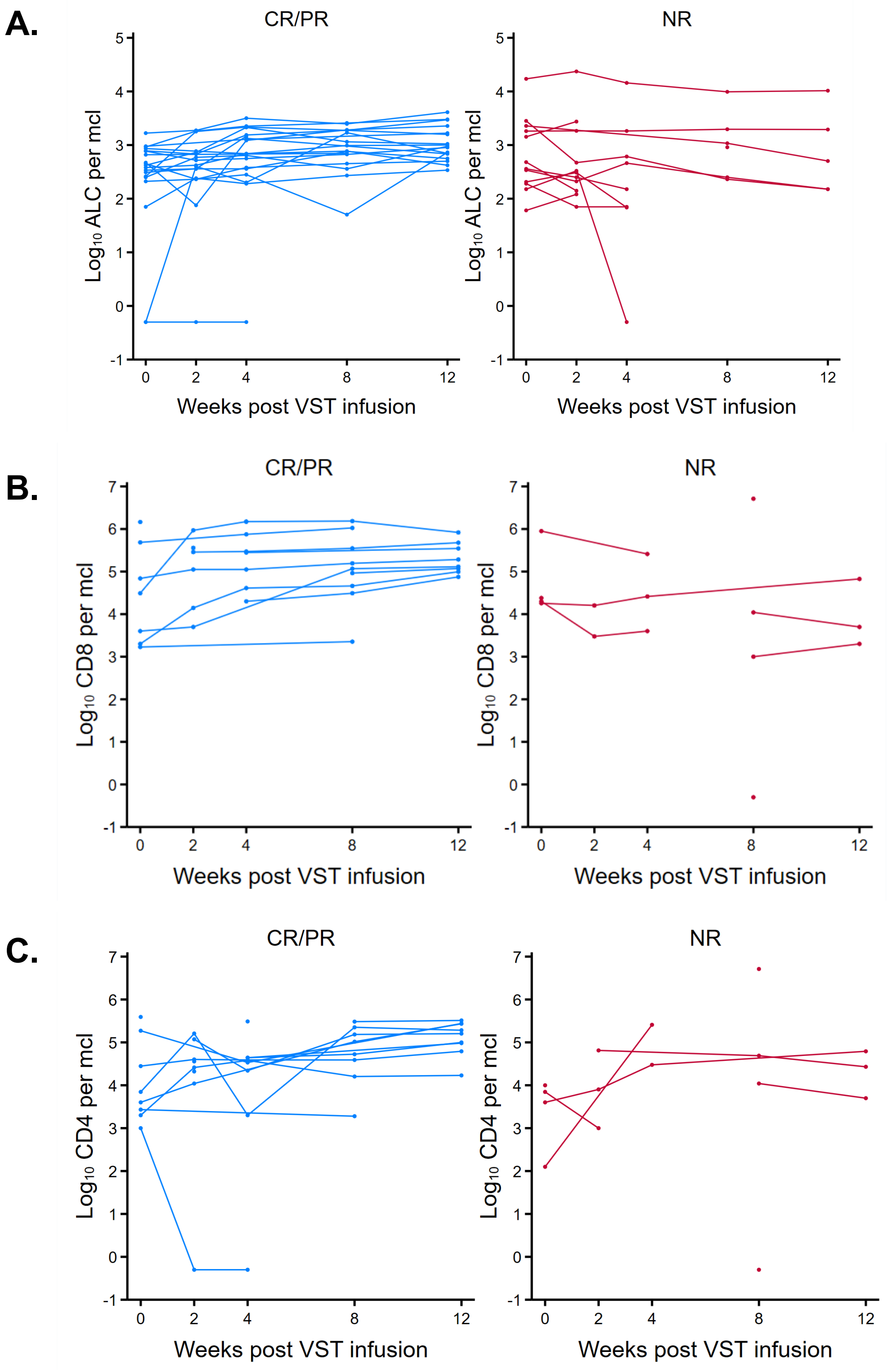

Supplementary Figure 9: Longitudinal lymphocyte counts by antiviral response by mixed model analysis. A. Absolute lymphocyte counts in complete/partial responders vs non-responders (n=33). B. Absolute CD8 counts in complete/partial responders vs non-responders (n=21). C. Absolute CD4 counts in complete/partial responders vs non-responders (n=22).

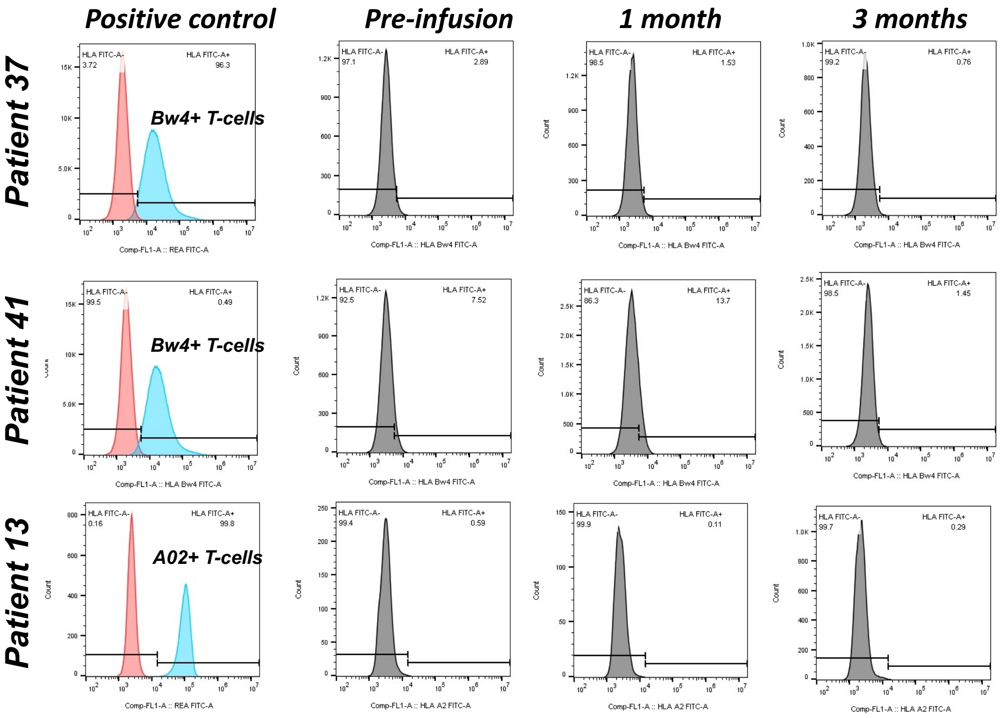

Supplementary Figure 10: Identities of dominant antiviral T cells by HLA staining. Virus-specific T cells from recipients based on IFNγ+/TNFα+ expression following viral peptide library restimulation were gated and evaluated for expression of discordant HLA receptors that differ between the VST donor and recipient/BMT donor. Example plots from patient 37 (BW4 on the X axis), patient 41 (BW4), and patient 13 (A02) are displayed.

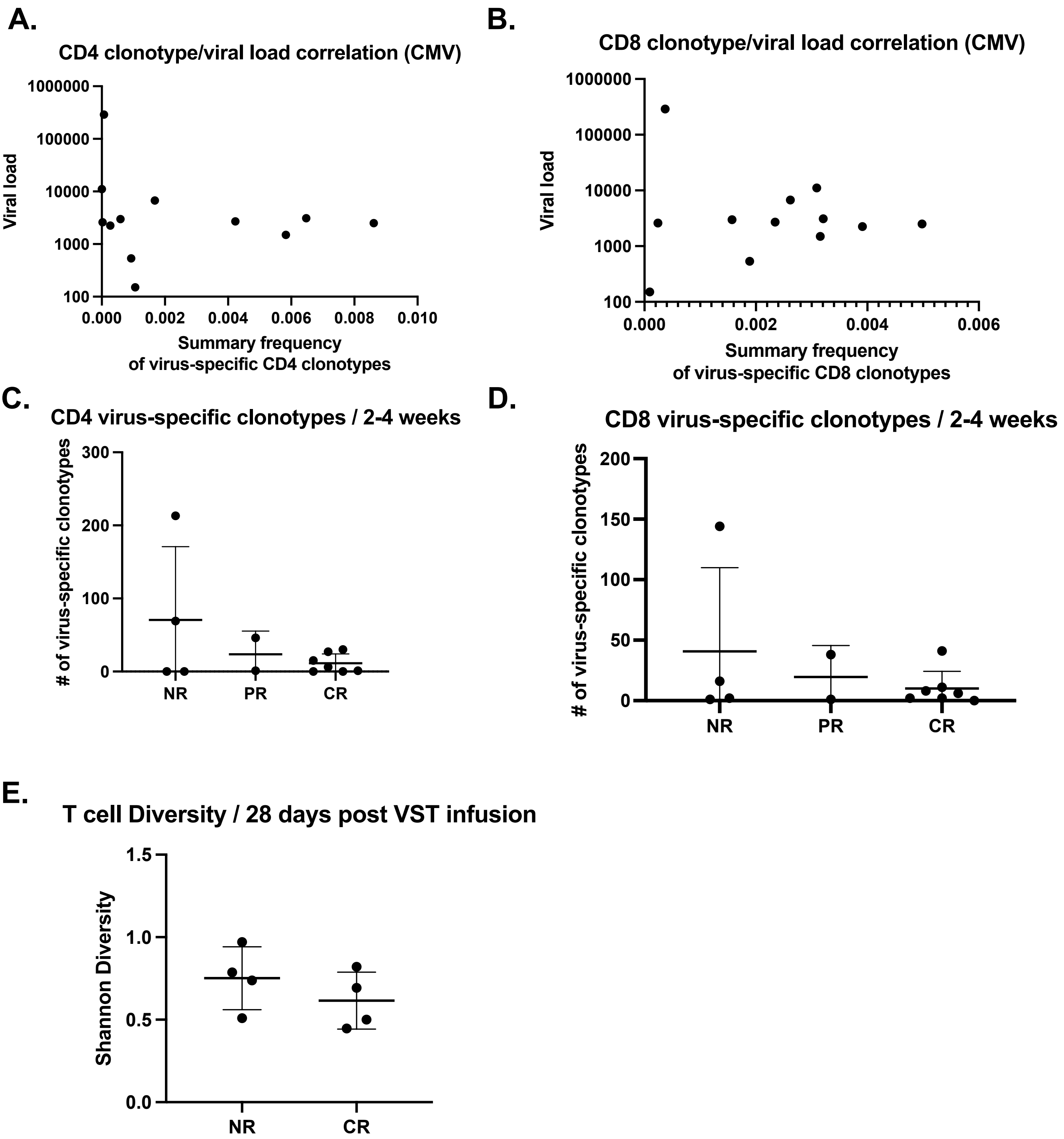

Supplementary Figure 11: Analysis of TCR results. A. CD4-associated clonotype frequencies versus CMV viral loads (IU, n=13). B. CD8-assoiated clonotype frequencies vs CMV viral loads (IU, n=13). C. Total CD4-associated clonotypes vs antiviral response at 2-4 weeks post infusion(n=9, bar: mean; whiskers: standard deviation). D. Total CD8-associated clonotypes vs antiviral responses at 2-4 weeks post infusion(n=9, bar: mean; whiskers: standard deviation). E. Shannon entropy index in complete responders (CR, n=4) versus non-responders (NR, n=4) at day 38. Bar: mean; whiskers: standard deviation

A.

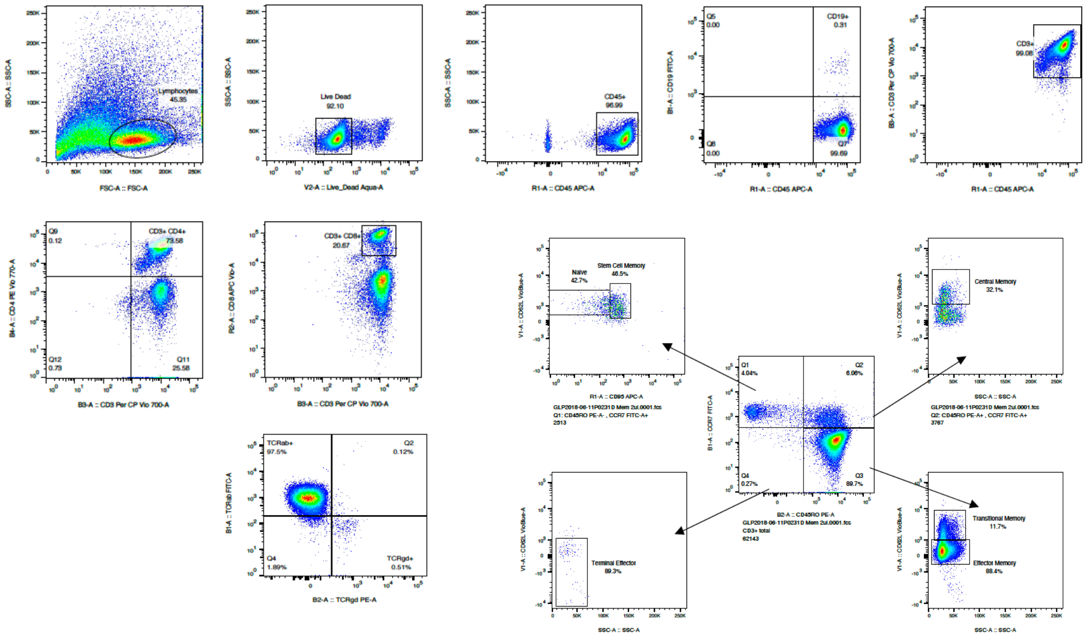

B.

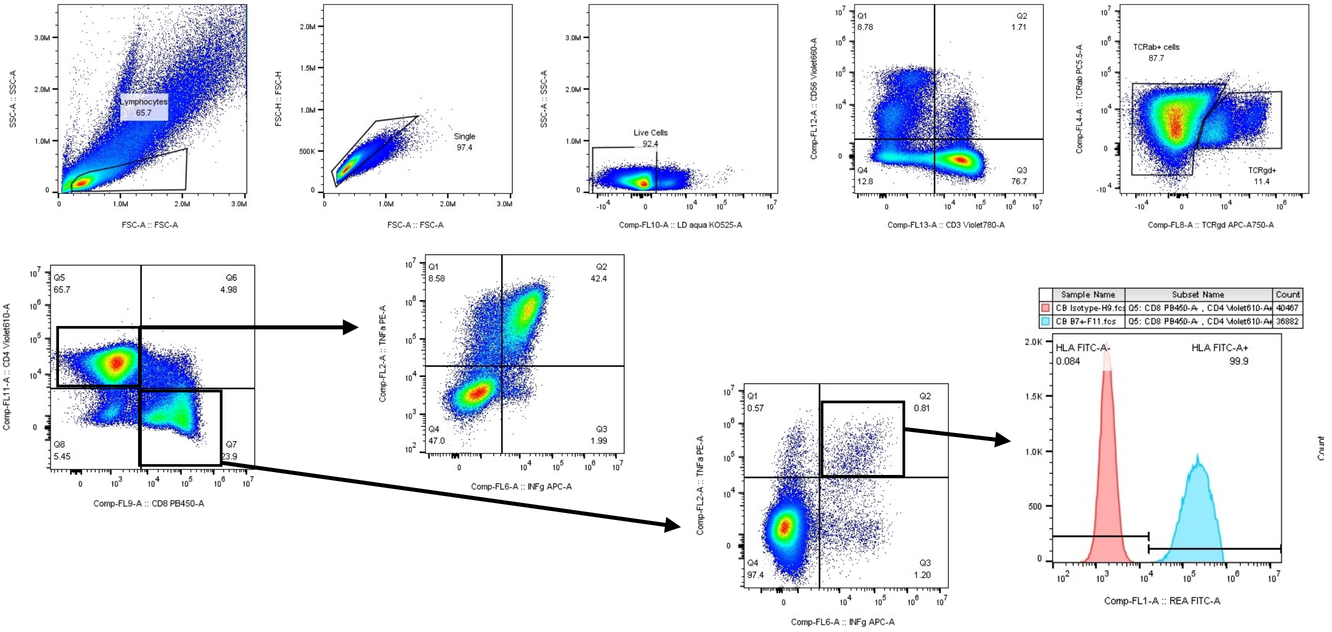

Supplementary Figure 12: Flow cytometry gating strategies. A. Gating strategy for Virus-specific T cell phenotyping (utilized for Figure 1, Supplemental Figure 4). B. Gating strategy for intracellular cytokine staining (utilized for Figure 5, Supplemental Figure 6).

Supplementary Table 1: Clinical Characteristics and Outcomes of Study Patients

Patient #	Age (yr)	Dx	BMT donor	Conditioning type	T cell depletion	Pre-infusion GVHD	Immuno-suppression	Target viral infection (primary: bold)	Duration of Viral infection	Tissue disease (y/n)	Recipient/ Donor serostatus	Previous antiviral agents	Peak viral load at first infusion*	Co-morbidities	Day post HSCT at first VST Infusion	# of VST infusions	HLA match (low res) with recipient	Shared HLA restriction(s)	Best response	Post-VST course	Status / Cause of death (days)
1	14	ALL	MMRD	MAC	ATG+abTCR depletion	No	None	CMV	85	No	CMV: Pos/Pos	Valganciclovir	37927	TMA	95	2	1/10, 1/10	A01 (D1, D2)	CR	Clearance of viremia, no adverse events.	Alive and well
2	7	HLH	MMRD	RIC	Anti-CD52	No	FK	Adv	80	Yes	NA	Brincidofovir	5237	None	113	1	4/10	Unknown	CR	Clearance of viremia, no adverse events.	Alive and well
3	10	T-ALL	UCBT	MAC	ATG	Yes	CsA, MMF, Methylpred.	Adv	15	No	NA	Cidofovir	6700	None	63	1	3/10	DRB1*04:01	CR	Clearance of adenoviremia. Fever, pulmonary and pericardial effusions (+20 post VST) in setting of BKV-associated hemorrhagic cystitis, treated with lasix.	Alive and well
4	2	XCGD	MUD	MAC	None	No	FK, MMF, eculizumab	Adv	15	No	NA	Cidofovir	14351	TMA, VOD, respiratory failure, renal failure	105	1	3/10	Unknown	NE	No improvement in adenoviremia.	Died, VOD (+27 post VST)
5	4	AML	UCBT	MAC	ATG	No	None	Adv	39	No	NA	Cidofovir	1400000	None	56	2	5/10, 5/10	Unknown	CR	Clearance of viremia, no adverse events.	Alive and well
6	6	ALL	MMRD	MAC	ATG+abTCR depletion	Yes	Methylpred.	Adv	30	Yes	NA	Cidofovir	22259	VOD, TMA	64	1	2/10	DRB1*15	NR	Progressive respiratory disease on day +2.	Died, progressive viral disease (+12 post VST)
7	15	HbSS	UCBT	MAC	None	Yes	FK, Infliximab	CMV	254	Yes		Ganciclovir	3500	None	401	3	4/10, 3/10, 2/10	A68 (D1, D2), Unknown (D3)	NR	Ongoing retinitis requiring intrathecal ganciclovir and foscarnet, no adverse events.	Alive and well
8	9	STAT3 GOF	MSD	MAC	Anti-CD52	No	Prednisolone, FK	Adv	18	No	NA	Cidofovir	131000	None	36	1	5/10	DRB1*07:01	CR	Clearance of viremia, no adverse events.	Died, disease recurrence (+162 post VST)
9	7	HLH/CAEBV	MUD	RIC	Anti-CD52	No	None	Adv	28	No	NA	Cidofovir	3253	None	134	1	3/10	DRB1*04	CR	Clearance of viremia, no adverse events.	Alive and well
10	6	Beta-thalassemia	MMUD	MAC	None	Yes	Hydrocortisone, FK	CMV	60	Yes	CMV: Pos/Neg	None	2650	None	175	3	5/10, 3/10, 5/10	A02:01 (D1, D2, D3)	PR	Reduction in viral load after third infusion, no adverse events. Received additional VST therapy off-study.	Alive and well
11	23	ALL	MMRD	MAC	None	No	None	CMV	19	Yes	CMV: Pos/Pos	Ganciclovir, Foscarnet	<500 / +ocular PCR	None	476	2	4/10, 4/10	Unknown	PR	Improvement in CMV retinitis on exam. No adverse events.	Alive and well
12	13	CAEBV	MMRD	RIC	Anti-CD52	No	Methylpred.	Adv	35	No	NA	Brincidofovir	5454	Respiratory failure, Renal failure	177	2	4/10, 4/10	DRB1*04 (D1, D2)	CR	Received stress dose hydrocortisone (+60 post VST); transient positive/NQ adenovirus thereafter, which resolved.	Alive and well
13	18	GATA2	MMUD	MAC	ATG	No	FK	CMV	82	No	CMV: Pos/Pos	None	278000	None	249	1	3/10	B08	PR	Clearance of viremia, no adverse events.	Alive and well
14	13	CARD9	MMRD	RIC	None	No	Sirolimus, Eculizumab	CMV	29	Yes	CMV: Pos/Pos	Ganciclovir	6025	Respiratory failure, Renal failure	51	1	3/10	A03:01	NE	Hypotension and hypoxemia(+6 post VST). Received toculizumab.	Died, CMV pneumonitis (+8 post VST)
15	1	XSCID	MSD	None	None	No	FK	Adv	35	Yes	NA	Cidofovir, Brincidofovir	32000	Respiratory failure, Renal failure	16	2	2/10, 2/10	DRB1*01 (D1, D2)	CR	Gradual decrease in adenoviremia. Underwent second transplant due to lack of primary engraftment (+111 post VST), no further adenoviral disease post-HSCT.	Alive and well
16	13	T cell lymphoma	MMUD	MAC	None	No	None	Adv	71	Yes	NA	Brincidofovir	2000000	None	98	1	3/10	A02:01	NR	No improvement in adenoviremia.	Died, progressive viral disease (+16 post VST)

17	7 mo.	SCID	Pre-HSCT	None	None	No	None	CMV	172	Yes		Foscarnet	199727	Respiratory failure	N/A	1	2/10	A03:01	NE	Worsening of pre-existing respiratory failure/hypoxemia (+4 post VST).	Died, CMV pneumonitis (+8 post VST)
18	19	B-ALL	MMRD	MAC	ATG+abTCR depletion	No	None	**Adv** & CMV	36	Yes		Ganciclovir, Cidofovir	4066(Adv)	Renal failure, VOD	41	2	4/10, 2/10	DRB1*07:01	PR	Reduction of adenoviremia to <500 copies/ml. Worsening of pre-existing VOD. Underwent liver transplant (day +31 post VST), complicated by pancytopenia secondary to engraftment of lymphocytes from solid organ.	Died, fungal pneumonia (+77 post VST)
19	7	HbSS	MSD	MAC	Anti-CD52	No	FK	CMV	37	No	CMV: Pos/Pos	Foscarnet	30600	None	48	1	3/10	A02:01	CR	Clearance of viremia, no adverse events.	Alive and well
20	22	MDS	MMRD	MAC	ATG+abTCR depletion	Yes	Budesonide	CMV	63	No	CMV: Pos/Pos	Ganciclovir, Foscarnet	1764	None	75	2	2/10, 3/10	A01 (D1), A01 & A02 (D2)	NR	Clearance of viremia, recurrence of CMV retinitis (+96 post VST)	Alive
21	14	ALL	MSD	MAC	None	Yes	MMF, FK	Adv	NA	No	NA	Brincidofovir	10235	Respiratory failure, Renal failure	133	1	2/10	DRB1*04(predicted)	CR	Worsening hypoxia in setting of pre-existing respiratory failure (day +4 post VST), recovered .	Alive and well
22	17	AML	MMRD	MAC	ATG+abTCR depletion	Yes	Prednisone, FK, ECP	Adv	29	No	NA	Cidofovir	99924	None	152	1	3/10	Unknown	NR	No improvement in adenoviremia. Steroids given in setting of worsening GI-GVHD (+27 post VST)	Died, progressive adenoviremia and fungal PNA (+39 post VST)
23	20	AML	MSD	MAC	None	No	MMF, FK	Adv	30	Yes	NA	Cidofovir, Brincidofovir	15222	Renal failure	40	1	5/10	DRB1*04	CR	Renal hemorrhage in setting of adenoviral hemorrhagic cystitis (day +6 post VST).	Died, bacterial sepsis (+201 post VST)
24	4 mo.	SCID	MMRD	RIC	ATG+abTCR depletion	No	None	CMV	36	Yes	CMV: Pos/Pos	Foscarnet, Cidofovir	78459	None	42	1	1/10	A68	NR	Secondary graft rejection (+20 post VST). Received ATG and steroids. 2nd transplant (MMRD) after ATG/thio/flu/Cy conditioning (day +45 post VST)	Died, bacterial sepsis (+62 post VST)
25	12	ALL	MUD	RIC	None	Yes	Prednisone, Sirolimus	**Adv** & CMV	17	No		Ganciclovir	61000 (Adv), 2300 (CMV)	Renal failure, TMA	99	1	5/10	DRB1*15	CR	Clearance of viremia, no adverse events.	Alive and well
26	1 mo.	SCID	N/A	None	None	No	None	CMV	16	Yes	CMV: Pos (pre-HSCT)	Ganciclovir, Foscarnet	138953	None	N/A	1	4/10	A02:01	NR	No improvement in CMV post VST infusion. Underwent unconditioned CD34-selected MMRD HSCT (+23 post VST), and subsequent 2nd MMRD HSCT following bu/flu conditioning (+98 post VST).	Died, progressive CMV disease (+124 post VST)
27	1	AML	MUD	MAC		No	FK, MTX	CMV	18	No	CMV: Pos/Pos	Ganciclovir	1800	Renal failure, VOD	39	1	5/10	A23, B44	CR	Clearance of viremia, no adverse events.	Alive and well
28	2	NEMO	UCBT	MAC	Anti-CD52	Yes	Vedoluzimab, FK, prednisone	Adv	50	No	NA	Cidofovir	1100	None	386	1	4/10	Unknown	CR	Clearance of viremia, no adverse events.	Alive and well
29	1	CHH	MUD	MAC	Anti-CD52	No	FK, CsA	Adv	38	Yes	NA	Cidofovir	4900000	None	37	2	6/10, 3/10	A01:01 (D1), DRB1*01:02 (D2)	CR	Clearance of viremia, no adverse events.	Alive and well
30	1	CGD	MUD	MAC	Anti-CD52	No	Prednisone, Vedolizumab, Tocilizumab, Infliximab	Adv	34	Yes	NA	Cidofovir	1700000	None	46	1	2/10	DRB1*01:02	CR	Clearance of viremia, no adverse events.	Alive and well
31	21	NK/T lymphoma	MMRD	RIC	ATG+abTCR depletion	Yes	Hydrocortisone, Sirolimus, leflunomide	Adv	22	Yes	NA	Cidofovir	1350000	Renal failure	133	3	4/10, 4/10, 1/10	Unknown	CR	Clearance of viremia, no adverse events.	Alive and well
32	7 mo.	DGS	N/A	None	None	Yes	FK	Adv	63	Yes	NA	Cidofovir	388000	Respiratory failure	N/A	2	3/10, 4/10	Unknown	NR	No improvement in adenoviremia, no adverse events	Died, progressive viral disease (+71 post VST)
33	4	LCH	MUD	RIC	ATG	No	FK	CMV	39	No	CMV: Pos/Pos	Foscarnet	16723	None	40	1	2/10	A24, B07	NE	No change in viral lloads.	Died, VOD (+10 post VST)

34	4	ALL	MUD	MAC	ATG	Yes	None	EBV	102	Yes	EBV: Pos/ ND	Rituximab	Pos/CSF(NQ)	None	222	1	4/10	A02:01	PR	Improvement in lymphatic lesions on PET (+7 post VST), no further viremia. Grade III cGVHD(+282 post VST)	Alive
35	6	LIG4	MMUD	RIC	ATG	Yes	Prednisone, CsA, ECP, eculizumab	EBV	124	Yes	EBV: NA/ positive	Rituximab	6736	TMA	194	1	2/10	Unknown	NR	Respiratory failure, ascites (day +29) due to GVHD and PTLD.	Died, bacterial infection (124)
36	1	TTC7A	N/A	None	None	No	none	CMV	306	No	ND	Ganciclovir, Foscarnet	886	None	N/A	2	3/10, 2/10	A02:01 (D1, D2)	NR	No adverse events, no improvement in viral load	Alive
37	23	AML	MMRD	MAC	None	Yes	Prednisone, FK, ECP, Eculizumab, ruxolitinib	CMV	50	No	CMV: Neg/Pos	Ganciclovir, Cidofovir	2240	TMA	84	2	1/10, 1/10	A02:01(D1, D2)	CR	Clearance of viremia, no adverse events.	Alive and well
38	8	SAA	MUD	RIC	Anti-CD52	No	FK	Adv & **CMV**	56	No	CMV: Neg/ Pos	Ganciclovir	1167 (CMV), +/<190 (Adv)	None	68	1	2/10	A02:01	CR	Clearance of viremia, no adverse events.	Alive and well
39	16	AML	UCBT	MAC	ATG	Yes	Prednisone, Sirolimus, ECP, eculizumab	Adv	130	Yes	NA	Cidofovir	67267	TMA	322	1	2/10	A68(pred)	NR	Grade III CRS (day +10 post VST), given tociluzimab and hydrocortisone. Flaring of skin GVHD(grade III, day +24 post VST)	Died, progressive viral disease (+61 post VST)
40	10	ALL	MMRD	MAC	ATG+abTCR depletion	Yes	Methylpred., hydrocortisone, FK, eculizumab	Adv	74	Yes	NA	Cidofovir	2500000	TMA	87	1	3/10	Unknown	NR	No improvement in adenoviremia.	Died, progressive viral disease (+31 post VST)
41	16	XLP1	MUD	MAC	ATG	Yes	CsA	EBV	45	Yes	EBV: NA/ positive	Rituximab	Pos/NQ	None	147	1	5/10	A02:01	CR	Clearance of PTLD lesions on PET(+45 post VST). Low grade skin GVHD (+63 post VST).	Alive and well
42	2	AML	MMRD	RIC	Anti-CD52	No	FK	Adv	35	No	NA	Cidofovir	120000	None	72	1	3/10	DRB1*04	CR	Clearance of viremia, no adverse events.	Alive and well
43	15	ALL	MMRD	MAC	ATG+abTCR depletion	No	None	Adv	26	Yes	NA	Cidofovir	978938	Renal failure	30	2	3/10, 4/10	Unknown (D1), DRB1*04 (D2)	PR	No improvement in adenoviremia. Neurologic disease (Day +11 post VST); MRI consistent with PRES. CSF adenovirus PCR positive (Day +28 post VST). Worsening delirium (day +40), given anakinra, solumedrol, siltuximab, IVIG (day +44).	Died, progressive neurologic disesae (+56 post VST).
44	3	HbSS	MRD	MAC	Anti-CD52	No	FK	CMV	36	No	CMV: Pos/Pos	Foscarnet	2048	None	41	1	4/10	A02:01	CR	Clearance of viremia, no adverse events.	Alive and well
45	12	GATA2	MUD	MAC	ATG	Yes	Beclomethasone, solumedrol, eculizumab	**Adv** & CMV	42	Yes	NA	Cidofovir	31623(Adv)	Respiratory failure, renal failure, VOD, TMA	116	1	3/10	DRB1*07:01	NR	No improvement in adenoviremia.	Died, progressive viral disease (+33 post VST)
46	20	ALL	MMRD	RIC	ATG+abTCR depletion	Yes	Beclomethasone, eculizumab	Adv	190	No	NA	None	261000	TMA	251	1	3/10	DRB1*15	NR	Altered mental status, aphasia: given anakinra(day +44), siltuximab (day+58). Seizure (day +63), treated with decadron and keppra. EEG with diffuse axonal polyneuropathy (day +83), given plasmapheresis (day +86)	Died, progressive neurology disease (+97 post VST)
47	8	Beta-thal	MSD	MAC	ATG	No	FK,	Adv & **CMV**	53	No	CMV: Pos/Pos	Ganciclovir, Cidofovir	35799(CMV), Pos/NQ (Adv)	None	77	1	4/10	Unknown	CR	Clearance of viremia, no adverse events.	Alive and well
48	19	Lymphoma, SAA, SOT	UCBT	RIC	Anti-CD52	Yes	Prednisone, FK	**Adv** & CMV	35	Yes	CMV: Pos/Neg	Foscarnet, Cidofovir	2380(Adv), 1181 (CMV)	None	128	1	1/10	DRB1*15	NR	Intermittent viremia post VST infusion. Flaring of GI-GVHD (day +25 post VST), treated with beclomethasone	Died, progressive viral disease (+120 post VST)
49	5	Fanconi Anemia	MMUD	RIC	ATG	Yes	FK, Abatacept	EBV	32	Yes	NA	Rituximab	13820	None	116	1	4/10	A24, A02	NR	No impact on EBV viral load; mixed response on PET CT (+24 post VST). Received Blinotumumab (+34 post VST)	Alive and well

50	8	LRBA	MMRD	RIC	Anti-CD52	No	FK	Adv & **CMV**	32	Yes	CMV: Pos/Neg	Ganciclovir, Foscarnet, Cidofovir	8390(Adv), 31400(CMV)	None	48	1	3/10	DRB1*07	NE	Secondary graft rejection with 0% donor CD3 chimerism and evidence of autologous recipient reconstitution (day+6). Treated with ganciclovir, cidofovir with gradual stabilization.	Alive
51	9	SAA	MMRD	RIC	ATG+abTCR depletion	No	Methylpred, Eculizumab	Adv	17	Yes	NA	None	8365324	Respiratory failure, Renal failure, TMA	34	1	4/10	A02:01	NR	No improvement in adenoviremia.	Died, progressive viral disease (+19 post VST)

Abbreviations: ALL: acute lymphocytic leukemia; MMRD: Mismatched related donor, MAC: myeloablative conditioning, RIC: reduced intensity conditioning, XCGD: X-linked chronic granulomatous disease, AML: acute myelogenous leukemia, HbSS: Sickle cell anemia, STAT3 GOF: STAT3 gain of function, HLH: hemophagocytic lymphohistiocytosis, CAEBV: Chronic active Epstein-Barr virus, GATA2: GATA2 haploinsufficiency, CARD9: CARD9 deficiency, SCID: severe combined immunodeficiency (XSCID: X-linked SCID), MDS: myelodysplastic disorder, DGS: Complete Digeorge syndrome, NEMO: NEMO deficiency (ectodermal dysplasia with immunodeficiency), CHH: Cartilage-hair-hypoplasia, LCH: Langerhans cell histiocytosis, LIG4: Ligase 4 deficiency, TTC7A: TTC7A deficiency, XLP1: X-linked lymphoproliferative disease type 1, Beta-thal: Beta thalassemia, SOT: Solid organ transplantation, LRBA: LRBA deficiency, FK: Tacrolimus, ECP: Extracorporeal photopheresis, MTX: Methotrexate, SAA: severe aplastic anemia; MMF: mycophenolate mofetil; CsA: cyclosporin A; TMA: transplantation-associated thrombotic microangiopathy, NA: not available; CR: complete response; PR: partial response: NR: non-response; NE: not evaluable, D1: dose 1; D2: dose 2; D3: dose 3.

Supplementary Table 2: Severe Adverse Events

Patient #	SAE	CTCAE Grade	Serious Criteria	SAE day post-VST	SAE end day post-VST	Relationship to VST
14	Worsening renal failure	4	Hospitalization, life threatening, death	7		Unlikely related
	Pulmonary hypertension	4		6		Unlikely related
	Worsening pneumonitis	5		6		Unlikely related
3	Pleural effusion	3	Prolonged hospitalization	20	36	Unlikely related
	Pericardial effusion	3		20	30	Unlikely related
	Fever	2		17	19	Unlikely related
	BK Viremia	3		-52	30	Unrelated
	Pulmonary edema	1		20	36	Unlikely related
12	Renal and Urinary Disorders – Hemorrhagic Cystitis	4	Life threatening	20		Unlikely related
	Sepsis	4	Life threatening	23		Unlikely related
10	Fever	1	Hospitalization	22	29	Unrelated
7	Fever	1	Hospitalization	32		Unlikely related
	Cough	1		26		Unlikely related
	Fever	4	Hospitalization, medically important	121		Unlikely related
6	Hypoxia	5	Life threatening, death	2	12	Unrelated
18	Respiratory distress	3	Prolonged hospitalization, life threatening	20		Unrelated
	Acidosis	3		20		Unrelated
24	Graft rejection leading to secondary graft failure and pancytopenia	4	Prolonged hospitalization, life threatening, medically important	29		Definitely related
	Multi-organ system failure	5	Prolonged hospitalization, life threatening, death	60	62	Unrelated
	Sepsis due to pseudomonas aeruginosa	3		60	62	Unrelated
	Hepatic VOD	4		60	62	Unrelated
	Persistent CMV viremia	3		60	62	Unrelated

	Pancytopenia	4		29	62	Definitely related
39	Fever	2	Prolonged hospitalization, life threatening	9		Possibly Related
	Hypotension	4		9		Possibly Related
	Respiratory Distress	4		9		Possibly Related
	Diarrhea	1		9		Possibly Related
43	Neutrophil count decreased	4	Prolonged hospitalization, life threatening, death	5		Possibly Related
	Depressed level of consciousness	4		2		Possibly Related
	Depressed level of consciousness	5		0	16	Possibly related
	Hypoxia	5		29	56	Possibly related
35	Hypoxia	4	Life threatening, death	29	124	Possibly related
	Respiratory Failure	4		29	124	Possibly related
	Mulitsystem Organ Failure	5		124	124	Unrelated
	Septic Shock	5		123	124	Unrelated
46	Tremor	2	Prolonged hospitalization, Medically important, death	44	97	Unlikely related
	Dysphasia	3		44	97	Unlikely related
	Encephalopathy	3		44	97	Unlikely related
	Seizure	2		63	97	Unlikely related
	Peripheral neuropathy	3		44	97	Unlikely related
	Sensory and motor	3		76	97	Unlikely related
34	Fever	1	Hospitalization, medically important	192	192	Unrelated
	Neutropenia	2		168		Unrelated
	Blood & Lymphatic System Disorders - Other<br><br>DIC	3		191	202	Unrelated
	Thrombocytopenia	3		160		Unrelated
	Epistaxis	3		248		Unrelated
48	Respiratory Failure	5	Death	120	121	Unrelated
	Septic Shock	5		121	121	Unrelated
	EBV+PTLD	5		80	121	Unrelated

Table 3: Virus-specific clonotype overlap in patient samples with the infused VST product and with public TCR databases

**Patient#**	**Time point**	**Overlapping virus-specific clonotypes**			**Public virus-specific clonotypes (CMV)**			**Response**
		**CD3+**	**CD3/CD4+**	**CD3/CD8+**	**CD3/CD4+**	**CD3/CD8+**	**CD3+**	
5	Day 30/Dose 1		0	1	1	1		NR
	Day 30/Dose 2		0	0	8	0		CR
	Day 90/Dose 2		0	2	3	0		CR
7	Day 14/ Dose 1		213	144			794	NR
	Day 14/ Dose 2		150	96			592	NR
	Day 14/ Dose 3		175	126			749	NR
10	Day 17 /Dose 1		12	16			135	NR
	Day 15 /Dose 2		69	51			112	NR
	Day 16 /Dose 3		46	38			74	PR
15	Day 30/Dose 1		1	1	0	2		PR
	Day 30/Dose 2		0	2	7	3		CR
	Day 90/Dose 2		1	2	0	8		
19	Day 27		0	2			11	CR
25	Day 15		30	11			228	CR
27	Day 30		15	6			69	CR
37	Day 34 / Dose 1		0	2			43	NR
	Day 26 / Dose 2		6	8			41	CR
38	Day 30		27	41	32	81		CR
	Day 90		14	16	23	51		CR
43	Day 47 (CSF)	0					0	NE
46	Day 14	1					6	NR
	Day 30	5					14	
	Day 45	5					21	
	Day 44 (CSF)	48					4	

Supplementary Table 4: Approved study sites and institutional review board status

Approval	Institution	IRB Approval Date	SIV Date	Activated
Local IRB	Children's Hospital of Los Angeles*	Yes	4/16/18	5/2/18
	Children's Healthcare of Atlanta	6/27/19	9/6/18	7/23/19
	Children's Hospital Colorado	6/8/18	6/29/18	10/29/18
	City of Hope	8/21/18	9/6/18	10/9/18
	Columbia-Presbyterian/Columbia University Medical Center	12/11/18	12/26/18	2/1/19
	Fred Hutchinson / Seattle Children's Hospital	1/9/19	9/6/18	1/10/19
	Helen DeVos Children's Hospital	10/2/18	1/22/19	5/31/19
	Levine - Atrium Health	8/6/20	8/27/20	9/30/20
	Lurie Children's Hospital	8/8/19	9/3/19	9/4/19
	Medical University of S. Carolina	5/7/19	8/19/19	10/1/19
	Methodist Hospital	5/23/18	5/23/18	6/5/18
	Riley Children's - IU	9/11/18	9/6/18	1/28/19
	Roswell Park	6/12/19	9/3/19	10/14/19
	St Jude's Research Children's Hospital	2/13/19	6/17/19	8/15/19
	Stanford / Lucile Packard Children's Hospital	11/13/18	11/30/18	12/21/18
	Tufts Children's Hospital	9/11/18	10/15/18	10/16/18
	University of California Los Angeles	7/8/19	6/17/19	8/22/19
	University of Minnesota MHealth Fairview Masonic Children's Hospital	8/29/19	9/3/19	9/6/19
	Virginia Commonwealth University	1/17/19	3/22/19	9/6/19
	Washington University	3/21/19	5/20/19	5/28/19
	Yale University	1/30/19	8/23/19	9/3/19
Reliance	Dana Farber Institute and Boston Children's Hospital	5/6/19	6/17/19	6/20/19
	Children's Mercy Hospital	7/10/19	8/23/19	11/4/19
	Children's National Hospital	8/7/18	8/22/18	9/26/18
	Children's Hospital of Philadelphia	2/27/19	3/13/19	8/6/19
	Cleveland Clinic Foundation	5/28/19	11/1/19	4/13/20
	Duke University Health System	8/7/18	11/20/18	11/20/18
	Oregon Health and Science University	11/9/18	7/22/19	8/19/19
	Phoenix Children's Hospital	7/10/19	8/23/19	9/3/19

	University of California San Francisco	8/7/18	8/22/18	9/4/18
	C.S. Mott Children's Hospital, University of Michigan	9/9/19	4/29/19	10/7/19
	University of Texas, Southwestern Medical Center Dallas	5/28/19	8/23/19	8/28/19

*Central Institutional Review Board of Record

Supplementary Table 5: Intracellular staining antibody panel

**Tube**	**Antibodies**									
Channel	**PB450**	**KO525**	**BV610**	**BV660**	**BV780**	**FITC**	**PE**	**ECD**	**APC**	**APC-A700**
Color	BV421	Ghost 510	BV605	BV750	BV785			PE Dazzle	APC	Alexa 700
Ab	CD8	LD	CD4	CD56	CD3	HLA	TNFa	CD45RO	IFNg	CCR7

Supplementary Table 6: Product release surface antibody panel

PanLeuko	TCR	DC
CD14 VioBlue		CD14 VioBlue
CD19 FITC	TCRab FITC	HLA DR FITC
CD16/CD56 PE	TCRgd PE	CD83 PE
CD3 Per CP Vio 700	CD3 Per CP Vio 700	CD3 Per CP Vio 700
CD4 PE Vio 770	CD4 PE Vio 770	
CD45 APC	CD45 APC	CD45 APC
CD8 APV Vio770	CD8 APV Vio770	

Supplementary Table 7: Antibodies Details: Intracellular flow panel

Antibody	Manufacturer	Catalog #	Clone	Format	Vol/ test (1E6 cells)	Mfg references
Brilliant Violet 421™ anti-human CD8a Antibody	BioLegend	301036	RPA-T8	BV421	5ul	https://www.biolegend.com/en-us/products/brilliant-violet-421-anti-human-cd8a-antibody-7152
LIVE/DEAD™ Fixable Aqua Dead Cell Stain Kit, for 405 nm excitation	Invitrogen	L34966	N/A	Aqua	1ul/ml	https://www.thermofisher.com/document-connect/document-connect.html?url=https://assets.thermofisher.com/TFS-Assets%2FLSG%2Fmanuals%2Flive_dead_fixable_dead_cell_stains_man.pdf
Brilliant Violet 605™ anti-human CD4 Antibody	BioLegend	317438	OKT4	BV605	5ul	https://www.biolegend.com/en-us/products/brilliant-violet-605-anti-human-cd4-antibody-7820
Brilliant Violet 650™ anti-human CD56 (NCAM) Antibody	BioLegend	318344	HCD56	BV650	5ul	https://www.biolegend.com/en-us/products/brilliant-violet-650-anti-human-cd56-ncam-antibody-8780
Brilliant Violet 785™ anti-human CD3 Antibody	BioLegend	317330	OKT3	BV785	5ul	https://www.biolegend.com/en-us/products/brilliant-violet-785-anti-human-cd3-antibody-7977
PE anti-human TNF-α Antibody	BioLegend	502909	MAb11	PE	5ul	https://www.biolegend.com/en-us/products/pe-anti-human-tnf-alpha-antibody-1346
PE/Dazzle™ 594 anti-human CD45RO Antibody	BioLegend	304248	UCHL1	PE/Dazzle™ 594	5ul	https://www.biolegend.com/en-us/products/pe-dazzle-594-anti-human-cd45ro-antibody-12489
PerCP/Cyanine5.5 anti-human TCR α/β Antibody	BioLegend	306724	IP26	PerCP/Cyanine5.5	5ul	https://www.biolegend.com/en-us/products/percp-cyanine5-5-anti-human-tcr-alpha-beta-antibody-9232
PE/Cyanine7 anti-human CD107a (LAMP-1) Antibody	BioLegend	328618	H4A3	PE/Cyanine7	5ul	https://www.biolegend.com/en-us/products/pe-cyanine7-anti-human-cd107a-lamp-1-antibody-7707
APC anti-human IFN-γ Antibody	BioLegend	502512	4S.B3	APC	5ul	https://www.biolegend.com/en-us/products/apc-anti-human-ifn-gamma-antibody-1012
Alexa Fluor® 700 anti-human CD197 (CCR7) Antibody	BioLegend	353244	G043H7	AF700	5ul	https://www.biolegend.com/en-us/products/alexa-fluor-700-anti-human-cd197-ccr7-antibody-13407
APC/Fire™ 750 anti-human TCR γ/δ Antibody	BioLegend	331228	B1	APC/Fire™ 750	5ul	https://www.biolegend.com/en-us/products/apc-fire-750-anti-human-tcr-gamma-delta-antibody-14101

Supplementary Table 8: HLA Antibody details

HLA Specificity	Manufacturer	Splits and Associated Ab	Catalog #	Clone	Channels	Volume used/cells	Mfg references
**A2**	Milteyni	A203#, A210#	130-118-969	REA517	FITC	2 ul / 200k cells	https://www.miltenyibiotec.com/US-en/products/hla-a2-antibody-anti-human-reafinity-rea517.html#conjugate=fitc:size=100-tests-in-200-ul
**A3**	Milteyni	N/A	130-115-739	REA950	FITC	2 ul / 200k cells	https://www.miltenyibiotec.com/US-en/products/hla-a3-antibody-anti-human-reafinity-rea950.html#conjugate=fitc:size=100-tests-in-200-ul
**A28:A2**	Milteyni	A68, A69	130-099-601	REA142	FITC	10 ul / 200k cells	https://www.miltenyibiotec.com/US-en/products/hla-a2-a28-antibody-anti-human-reafinity-rea142.html#conjugate=fitc:size=100-tests-in-200-ul
**A9**	Milteyni	A23, A24, A2403#	130-099-524	REA127	FITC	2 ul / 200k cells	https://www.miltenyibiotec.com/US-en/products/hla-a9-antibody-anti-human-reafinity-rea127.html#conjugate=fitc:size=100-tests-in-1-ml
**A30:A31**	One Lambda	N/A	Steptavidin FITC: 405201	Not appliable	Biotin (+ Streptavidin FITC and PE conjugates)	2ul/200k cells	N/A
**B7**	Thermo(FITC)	B703#	MA1-82180	BB7.1	FITC	10 ul / 200k cells	https://www.thermofisher.com/antibody/product/HLA-B7-Antibody-clone-BB7-1-Monoclonal/MA1-82180
**B12**	Milteyni	B44,B45	130-099-862	REA138	FITC	30 tests in 300ul	https://www.miltenyibiotec.com/US-en/products/hla-b12-antibody-anti-human-reafinity-rea138.html#conjugate=fitc:size=30-tests-in-300-ul
**Bw4**	Milteyni	B5, B5102, B5103, B13, B17, B27, B37, B38(16), B44(12), B47, B49(21), B51(5), B52(5), B53, B57(17), B58(17), B59, B63(15), B77(15) A9, A23(9), A24(9), A2403, A25(10), A32(19)	130-103-846	REA274	FITC	10ul/200k cells	https://www.miltenyibiotec.com/US-en/products/hla-class-i-bw4-antibody-anti-human-reafinity-rea274.html#conjugate=pe:size=30-tests-in-60-ul
**Bw6**	Milteyni	B7, B703, B8, B14, B18, B22, B2708, B35, B39(16), B3901, B3902, B40, B4005, B41, B42, B45(12), B46, B48, B50(21),	130-123-264	REA143	FITC	2 ul / 200k cells	https://www.miltenyibiotec.com/US-en/products/hla-class-i-bw6-antibody-anti-human-reafinity-rea143.html#conjugate=fitc:size=100-tests-in-200-ul

		B54(22), B55(22), B56(22), B60(40), B61(40), B62(15), B64(14), B65(14), B67, B70, B71(70), B72(70), B73, B75(15), B76(15), B78, B81, B82					
B7, B27	Miltenyi	B27 splits to B2708#	130-120-234	REA176	FITC	2 ul / 200k cells	https://www.miltenyibiotec.com/US-en/products/hla-b7-b27-antibody-anti-human-reafinity-rea176.html#conjugate=vio-bright-fitc:size=100-tests-in-200-ul
B7, B27	Miltenyi	B27 splits to B2708#	130-120-308	REA176	FITC	2 ul / 1E6 cells	https://www.miltenyibiotec.com/US-en/products/hla-b7-b27-antibody-anti-human-reafinity-rea176.html#conjugate=vio-bright-fitc:size=30-tests-in-60-ul
HLA_A33, B8	Life Span Biosci	N/A	LS-C24464-100	Not appliable	unconjugated	Use as manufacturer's guide	https://www.lsbio.com/antibodies/hla-a33b8-antibody-ls-c24464/25046
Anti-HLA-A24 (Human) mAb	MBL international corp.	A2403#	K0208-4 & K0208-5	17A10	FITC	Use as manufacturer's guide	https://www.mblbio.com/bio/g/dtl/A/?pcd=K0208-5

Supplementary Table 9: Product release surface staining panel

Antibody/fluorophore	Vendor	Catalog Number	Clone	Volume Used/2E5 cells	**Mfg references**
CD45 APC	Miltenyi	130-110-633	REA747	1ul	https://www.miltenyibiotec.com/US-en/products/cd45-antibody-anti-human-reafinity-rea747.html#conjugate=apc:size=100-tests-in-200-ul
CD3 PerCP Vio 700	Miltenyi	130-113-141	REA613	1ul	https://www.miltenyibiotec.com/US-en/products/cd3-antibody-anti-human-reafinity-rea613.html#conjugate=percp-vio-700:size=100-tests-in-200-ul
CD19 FITC	Miltenyi	130-113-645	REA675	1ul	https://www.miltenyibiotec.com/US-en/products/cd19-antibody-anti-human-reafinity-rea675.html#conjugate=fitc:size=100-tests-in-200-ul
CD14 VioBlue	Miltenyi	130-110-524	REA599	2ul	https://www.miltenyibiotec.com/US-en/products/cd14-antibody-anti-human-reafinity-rea599.html#conjugate=vioblue:size=100-tests-in-200-ul
CD4 PE Vio770	Miltenyi	130-113-227	REA623	1ul	https://www.miltenyibiotec.com/US-en/products/cd4-antibody-anti-human-reafinity-rea623.html#conjugate=pe-vio-770:size=100-tests-in-200-ul
CD8 APC Vio770	Miltenyi	130-110-681	REA734	1ul	https://www.miltenyibiotec.com/US-en/products/cd8-antibody-anti-human-reafinity-rea734.html#conjugate=apc-vio-770:size=100-tests-in-200-ul
CD16 PE	Miltenyi	130-113-393	REA423	1ul	https://www.miltenyibiotec.com/US-en/products/cd16-antibody-anti-human-reafinity-rea423.html#conjugate=pe:size=100-tests-in-200-ul
CD 56 PE	Miltenyi	130-113-312	REA196	1ul	https://www.miltenyibiotec.com/US-en/products/cd56-antibody-anti-human-reafinity-rea196.html#conjugate=pe:size=100-tests-in-200-ul
CD83 PE	Miltenyi	130-110-503	REA714	1ul	https://www.miltenyibiotec.com/US-en/products/cd83-antibody-anti-human-reafinity-rea714.html#conjugate=pe:size=100-tests-in-200-ul
TCRab FITC	Miltenyi	130-113-538	RES652	1ul	https://www.miltenyibiotec.com/US-en/products/tcra-b-antibody-anti-human-reafinity-rea652.html#conjugate=fitc:size=100-tests-in-200-ul
TCRgd PE	Miltenyi	130-113-512	REA591	1ul	https://www.miltenyibiotec.com/US-en/products/tcrg-d-antibody-anti-human-reafinity-rea591.html#conjugate=pe:size=100-tests-in-200-ul
CD45RO PE	Miltenyi	130-113-559	REA611	2ul	https://www.miltenyibiotec.com/US-en/products/tcrg-d-antibody-anti-human-reafinity-rea591.html#conjugate=pe:size=100-tests-in-200-ul
CCR7 FITC	Miltenyi	130-120-468	REA546	2ul	https://www.miltenyibiotec.com/US-en/products/cd197-ccr7-antibody-anti-human-reafinity-rea546.html#conjugate=fitc:size=100-tests-in-200-ul
CD95 APC	BD Bio	558814	DX2	2ul	https://www.bdbiosciences.com/en-us/products/reagents/flow-cytometry-reagents/research-reagents/single-color-antibodies-ruo/apc-mouse-anti-human-cd95.558814
CD62 VioBlue	Miltenyi	130-113-622	145/15	2ul	https://www.miltenyibiotec.com/US-en/products/cd62l-antibody-anti-human-145-15.html#conjugate=vioblue:size=100-tests-in-200-ul
HLA DR FITC	Miltenyi	30-111-788	REA805	1ul	https://www.miltenyibiotec.com/US-en/products/hla-dr-antibody-anti-human-reafinity-rea805.html#conjugate=vio-bright-r720:size=100-tests-in-200-ul
FcR Blocking Reagent	Miltenyi	130-059-901	N/A	10ul	https://www.miltenyibiotec.com/US-en/products/fcr-blocking-reagent-human.html#130-059-901

Supplementary Table 10: TCR Primers

Primer library	Sequence
TCRB constant primer	5’-TGCTTCTGATGGCTCAAACACAGCGACCT-3’
P5 Graft/ P5 Seq	5’-AATGATACGGCGACCACCGAGATCTACACTCTTTCCCTACACGACGCTCTTCCGATCT-3’
SMARTer II A	5’-AAGCAGTGGTATCAACGCAGAGTACATrGrGrG-3’

Supplementary Table 11: CONSORT Checklist

Section	Item No.	CONSORT 2010 Item	CONSORT-Outcomes 2022 item	Location Reported[b]
**Title and abstract**				
	1a	Identification as a randomized trial in the title	-	N/A
	1b	Structured summary of trial design, methods, results, and conclusions (for specific guidance see CONSORT for abstracts)	-	Confirmed/ abstract
**Introduction**				
Background and objectives	2a	Scientific background and explanation of rationale	-	Intro, p.6
	2b	Specific objectives or hypotheses	-	Intro, p.7
**Methods**				
Trial design	3a	Description of trial design (such as parallel, factorial) including allocation ratio	-	Methods, p.18
	3b	Important changes to methods after trial commencement (such as eligibility criteria), with reasons	-	N/A
Participants	4a	Eligibility criteria for participants	-	Methods p.18
	4b	Settings and locations where the data were collected	-	Methods p.19
Interventions	5	The interventions for each group with sufficient details to allow replication, including how and when they were actually administered (for specific guidance see TIDieR checklist and guide)	-	Methods p.20-21
Outcomes	6a	Completely defined prespecified primary and secondary outcome measures, including how and when they were assessed	-	Methods p. 20-21
	6a.1		Provide a rationale for the selection of the domain for the trial's primary outcome	Intro, p.7
	6a.2		Describe the specific measurement variable (eg, systolic blood pressure), analysis metric (eg, change from baseline, final value, time to event), method of aggregation (eg, mean,	Methods p. 20-22

© 2022 American Medical Association. All rights reserved.

Section	Item No.	CONSORT 2010 Item	CONSORT-Outcomes 2022 item	Location Reported[b]
			proportion), and the time point for each outcome	
	6a.3		If the analysis metric for the primary outcome represents within-participant change, define and justify the minimal important change in individuals	Methods, p. 20-21
	6a.4		If the outcome data were continuous, but were analyzed as categorical (method of aggregation), specify the cutoff values used	N/A
	6a.5		If outcome assessments were performed at several time points after randomization, state the time points used for the analysis	N/A
	6a.6		If a composite outcome was used, define all individual components of the composite outcome	N/A
	6a.7		Identify any outcomes that were not prespecified in a trial registry or trial protocol	N/A
	6a.8		Provide a description of the study instruments used to assess the outcome (eg, questionnaires, laboratory tests) along with reliability, validity, and responsiveness in a population similar to the study sample	Methods, p20-21
	6a.9		Describe who assessed the outcome (eg, nurse, parent) and any qualifications or trial-specific training necessary to administer the study instruments to assess the outcome	Methods p.22
	6a.10		Describe any processes used to promote outcome data quality during data collection (eg, duplicate measurements) and after data collection (eg, range checks of outcome data values), or state where these details can be found	Methods, p.21-22
	6b	Any changes to trial outcomes after the trial commenced, with reasons	-	N/A
Sample size	7a	How sample size was determined	-	Methods p.18

© 2022 American Medical Association. All rights reserved.

Section	Item No.	CONSORT 2010 Item	CONSORT-Outcomes 2022 item	Location Reported[b]
	7a.1		Define and justify the target difference between treatment groups (eg, the minimal important difference)	Methods p.18
	7b	When applicable, explanation of any interim analyses and stopping guidelines	-	Methods p.19
**Randomization**				
Sequence generation	8a	Method used to generate the random allocation sequence	-	N/A
	8b	Type of randomization; details of any restriction (such as blocking and block size)	-	N/A
Allocation concealment mechanism	9	Mechanism used to implement the random allocation sequence (such as sequentially numbered containers), describing any steps taken to conceal the sequence until interventions were assigned	-	N/A
Implementation	10	Who generated the random allocation sequence, who enrolled participants, and who assigned participants to interventions	-	N/A
Blinding	11a	If done, who was blinded after assignment to interventions (for example, participants, care providers, those assessing outcomes) and how	-	N/A
	11b	If relevant, description of the similarity of interventions	-	N/A
Statistical methods	12a	Statistical methods used to compare groups for primary and secondary outcomes	-	Methods, p.23
	12a.1		Describe any methods used to account for multiplicity in the analysis or interpretation of the primary and secondary outcomes (eg, coprimary outcomes, same outcome assessed at multiple time points, or subgroup analyses of an outcome)	Methods, p.23
	12a.2		State and justify any criteria for excluding any outcome data from the analysis and reporting, or report that no outcome data were excluded	Methods, p.23

© 2022 American Medical Association. All rights reserved.

Section	Item No.	CONSORT 2010 Item	CONSORT-Outcomes 2022 item	Location Reported[b]
	12a.3		Describe the methods used to assess patterns of missingness (eg, missing not at random), and describe the methods used to handle missing outcome items or entire assessments	N/A
	12a.4		Provide a definition of the outcome analysis population relating to nonadherence of the trial protocol (eg, as a randomized analysis)	N/A
	12b	Methods for additional analyses, such as subgroup analyses and adjusted analyses	-	Methods, p.23
**Results**				
Participant flow (a diagram is strongly recommended)	13a	For each group, the numbers of participants who were randomly assigned, received intended treatment, and were analyzed for the primary outcome	-	Results, p 7-8
	13b	For each group, losses and exclusions after randomization, together with reasons	-	N/A
Recruitment	14a	Dates defining the periods of recruitment and follow-up	-	Results. p. 7
	14b	Why the trial ended or was stopped	-	Results, p.8
Baseline data	15	A table showing baseline demographic and clinical characteristics for each group	-	Table 1, p.34
Numbers analyzed	16	For each group, number of participants (denominator) included in each analysis and whether the analysis was by original assigned groups	-	Results, p.10
Outcomes and estimation	17a	For each primary and secondary outcome, results for each group, and the estimated effect size and its precision (such as 95% CI)	-	Results, p.10-11
	17a.1		Include the results for all prespecified outcome analyses or state where the results can be found if not in this report	Results, p.10-13
	17b	For binary outcomes, presentation of both absolute and relative effect sizes is recommended	-	Results, p.10-13
Ancillary analyses	18	Results of any other analyses performed, including subgroup analyses and adjusted	-	Results, p10-13

Section	Item No.	CONSORT 2010 Item	CONSORT-Outcomes 2022 item	Location Reported[b]
		analyses, distinguishing prespecified from exploratory		
	18.1		If there were any analyses that were not prespecified, explain why they were performed	Results, p.9
Harms	19	All important harms or unintended effects in each group (for specific guidance see CONSORT for harms)	-	Results, p9
**Discussion**				
Limitations	20	Trial limitations, addressing sources of potential bias, imprecision, and, if relevant, multiplicity of analyses	-	Discussion, p.17
Generalizability	21	Generalizability (external validity, applicability) of the trial findings	-	Discussion p17-18
Interpretation	22	Interpretation consistent with results, balancing benefits and harms, and considering other relevant evidence	-	Discussion p.14-18
**Other Information**				
Registration	23	Registration number and name of trial registry	-	Methods p.19
Protocol	24	Where the full trial protocol can be accessed, if available	-	Methods p.19
Funding	25	Sources of funding and other support (such as supply of drugs), role of funders	-	Page 30

Supplementary Table 12: CONSORT-Outcomes 2022 Extension items

Section	Item No.	CONSORT-Outcomes item	Location Reported[b]
**Methods**			
Outcomes	6a.1	Provide a rationale for the selection of the domain for the trial's primary outcome	Introduction, p.5-6
	6a.2	Describe the specific measurement variable (eg, systolic blood pressure), analysis metric (eg, change from baseline, final value, time to event), method of aggregation (eg, mean, proportion), and the time point for each outcome	Methods p. 20-23
	6a.3	If the analysis metric for the primary outcome represents within-subject change, define and justify the minimal important change in individuals	Methods p.20-21
	6a.4	If the outcome data were continuous, but were analysed as categorical (method of aggregation), specify the cutoff values used	N/A
	6a.5	If outcome assessments were performed at several time points after randomization, state the time points used for the analysis	Methods, p.25
	6a.6	If a composite outcome was used, define all individual components of the composite outcome	N/A
	6a.7	Identify any outcomes that were not prespecified in a trial registry or protocol	N/A
	6a.8	Provide a description of the study instruments used to assess the outcome (eg, questionnaires, laboratory tests) along with reliability, validity, and responsiveness in a population similar to the study sample	Methods p 21-22
	6a.9	Describe who assessed the outcome (eg, nurse, parent), and any qualifications or trial-specific training necessary to administer the study instruments to assess the outcome	Methods p. 23
	6a.10	Describe any processes used to promote outcome data quality during data collection (eg, duplicate measurements) and after data collection (eg, range checks of outcome data values), or state where details can be found	Methods p.22-23
Sample size	7a.1	Define and justify the target difference between treatment groups (eg, the minimal important difference)	Methods p.18
Statistical methods	12a.1	Describe any methods used to account for multiplicity in the analysis or interpretation of the primary and secondary outcomes (eg, coprimary outcomes, same outcome assessed at multiple time points, or subgroup analyses of one outcome)	N/A
	12a.2	State and justify any criteria for excluding any outcome data from the analysis and reporting, or report that no outcome data were excluded	Methods, p.23

Section	Item No.	CONSORT-Outcomes item	Location Reported[b]
	12a.3	Describe methods to assess patterns of missingness (eg, missing not at random), and describe the methods to handle missing outcome items or entire assessments	N/A
	12a.4	Provide definition of outcome analysis population relating to protocol nonadherence (eg, as a randomized analysis)	N/A
**Results**			
Outcomes and estimation	17a.1	Include results for all prespecified outcome analyses or state where results can be found if not in this report	Results, p7-13
Ancillary analyses	18.1	If there were any analyses that were not prespecified, explain why they were performed	Results, p.9

Supplementary Note 1

Protocol: ***Antiviral Cellular Therapy for Enhancing T-cell Reconstitution Before or After Hematopoietic Stem Cell Transplantation (ACES)***
PBMTC SUP1701 / CLIN2-10392

Statistical Analysis plan

Treatment Efficacy Analysis (protocol section 5.3):
Efficacy, as measured by the proportion of patients achieving at least CR or PR (per section 3.1.3), will be assess independently in each of the three strata. There will be no interim monitoring for futility. As described above, the two primary strata will enroll a minimum of 20 and maximum of 30 evaluable patients. Under the null hypothesis that VST is not active, it is reasonable to assume that that the true spontaneous response rate will be no more than 20%. Thus in each of the two primary strata, based on an exact one-sided one-sample test of proportions of the null hypothesis that the response rate is ≤20%, with Type I error no greater than 5% and with a sample size of 20, there will be at least 90% power to detect an improvement in response rate to 52%. With a sample size of 30 the detectable improvement is to 47%. Hence this study has sufficient power to detect plausible and clinical important improvements in response rate due to VST in each of the primary strata. There will be no adjustment for multiple comparisons. Analysis of the exploratory stratum will be descriptive, consisting of a simple estimate of the proportion of patients who respond. In primary analysis patients with multiple infections will be included only in the stratum to which they are assigned, but may be included in other strata for secondary analysis.

Correlative Data Analysis (protocol section 5.5):
Safety and toxicity outcomes including adverse events, GvHD, clinical signs of viral infections, secondary graft failure and laboratory measurements will be summarized using descriptive statistics (frequency table, means, standard deviations, medians and ranges). Toxicity information including the type, severity, time of onset, time of resolution, and the probable association with the study regimen will be tabulated and summarized.

Response rate including complete response, partial response will be summarized as frequency table. The response rate with 95% CI will be reported. Reconstitution of antiviral immunity using IFN-g capture flow cytometry and ELIspot assays will also be summarized using descriptive statistics at each time point. Pairwise comparisons will be performed to compare changes of these immunological parameters from VST infusion to each time point of post-infusion measurements using paired t-tests or Wilcoxon signed-ranks tests. The normality assumption will be assessed and transformations to achieve approximate normality will be carried out if necessary. Overall survival post VST infusion will be analyzed by the Kaplan-Meier method.

Viral load levels will be correlated with immunological parameters using correlation coefficients. Longitudinal analysis is employed to model repeatedly-measured immunologic parameters. This will allow us to model patterns of immune response per patient while allowing for varying intercepts and slopes for a patient. Viral load levels will be correlated with immunological parameters using correlation coefficients. These modeling strategies will be considered exploratory in nature due to the limited patient numbers in this initial trial.

Extended statistical analysis plan:
Efficacy will be analyzed by response category as defined below, and time to partial and/or complete response (days post VST infusion at the time of response).
Response definitions (protocol section 3.1.3):

Complete response (CR): Return to normal range as defined by specific assay used for the targeted viral infection(s) in blood.
Partial response (PR): Decrease in viral load of at least 1-log from baseline. For EBV-PTLD >50% decrease in radiographic disease burden from baseline would also be considered a partial response.
Stable disease: Changes insufficient to qualify as partial response, but with <1 log increase in viral load with no evidence of dissemination to other sites of disease.
Progression: Increase in viral load of at least 1 log from baseline or dissemination to other sites of disease for at least one targeted infection.
Both stable disease and progressive disease are clustered into the category of **non-response (NR).**
Patients who do not have data at the time of follow-up evaluations are labeled as **non-evaluable (NE).**

Primary endpoints:

1. Rate of response is calculated as the proportion of responders (CR+PR) at day 30 post-infusion.
2. Feasibility is defined by two variables:
    a. Product availability: the number of referrals with one or more potential identified VST product(s) divided by the total number of referred patients.
    b. Days elapsed from time of study referral to the infusion of the VST product.
3. The safety endpoint is defined as the number of dose-limiting toxicities (DLT) in study subjects during the post-infusion monitoring period. DLT will be defined as acute GvHD grades III-IV or grades 3-5 infusion-related adverse events or grades 4-5 non-hematological adverse events related to the T cell product within 30 days of each VST dose and that are not due to the pre-existing infection or the original malignancy or pre-existing co-morbidities as defined by the NCI Common Terminology Criteria for Adverse Events (CTCAE), Version 4.03.

Specific statistical analyses / correlations:
-We will evaluate for correlations between:
    -Day of infusion post-BMT and chances(rate) of achieving a CR or PR?
    -Presence of pre-infusion GVHD (any grade) and chances of achieving a CR or PR?
    -Presence of low grade GVHD (grades I-II) and chances of achieving a CR or PR?
    -Presence of high grade GVHD (grades III-IV) and chances of achieving a CR or PR?
    -Use of any type of immunosuppression agents and chances of achieving a CR or PR?
        Specific immunosuppression agents evaluated:
            -corticosteroids (oral or parental)
            -Tacrolimus
            -Cyclosporin
            -mycophenolate mofetil
            -Sirolimus
            -Eculizumab
            -Other agents
    -Patient acuity and chances of achieving a CR or PR?
        -specific categories of patient acuity include ICU admission at infusion, respiratory failure, renal failures, transplant-associated microangiopathy, or veno-occlusive disease
        -A composite acuity variable was also analyzed where a "yes" to any of the above categories would be labeled as high acuity.

-Overall HLA match between VST donor and recipient (class I + class II) and chances of achieving a CR or PR?
-Confirmation of a shared antiviral HLA restriction and chances of achieving a CR or PR?
-Initial viral load and chances of achieving a CR or PR?
-Length of viral infection in days at the time of infusion versus chances of achieving a CR or PR?
-BMT conditioning type (myeloabelative versus reduced intensity conditioning) and chances of achieving a CR or PR?
-Use of specific BMT conditioning agents/regiments and chances of achieving a CR or PR?

    -Specific agents evaluated: a/bTCR/CD19 depletion, alemtuzumab, ATG.

-Are there any difference in trends in absolute lymphocyte counts, absolute CD4 counts, CD8 counts (AX-BB) over time (from baseline to 12 weeks post-infusion) in responders (CR+PR) vs non-responders?

Antiviral Cellular Therapy for Enhancing T-cell Reconstitution Before or After Hematopoietic Stem Cell Transplantation (ACES) PBMTC SUP1701

Pediatric Transplantation & Cellular Therapy Consortium

Primary Immune Deficiency Treatment Consortium

PRINCIPAL INVESTIGATORS
Michael A. Pulsipher, MD
Michael Keller, MD

STUDY COMMITTEE
Catherine Bollard, MD
Christopher Dvorak, MD
Patrick Hanley, PhD
C. Russell Cruz, PhD
Fahmida Hoq, MBBS, MS
Lauren McLaughlin, MD
Blachy Davila, MD
Donald Kohn, MD
Sung-Yun Pai, MD
Michael Verneris, MD
Roberta Adams, MD
Suhag Parikh, MD
Elizabeth Stenger, MD
Morris Kletzel, MD
Angela Smith, MD, MS
Ann Dahlberg, MD
Lauri Burroughs, MD
Sonata Jodele, MD

IND SPONSOR-INVESTIGATOR
Michael A. Pulsipher, MD

STATISTICIAN
Richard Sposto, Ph.D

Conducted under Investigator-held IND
17364

Protocol Version 5.0 11-May-2020(Amendment#4)

Coordinating Center
Pediatric Blood and Marrow Transplant Consortium (PBMTC) Operations Center
Children's Hospital Los Angeles
Performed in Collaboration with the Primary Immune Deficiency Treatment Consortium (PIDTC)

Cell Manufacturing Center
Program for Cell Enhancement and Technologies for Immunotherapy (CETI)
Children's National Hospital - Center for Cancer and Blood Disorders
111 Michigan Avenue, NW
Washington, DC 20010

Funding Sponsor
CIRM 2.0 California Institute for Regenerative Medicine

TABLE OF CONTENTS

PROTOCOL SYNOPSIS 4
STUDY SCHEMA 7
1 BACKGROUND AND RATIONALE 9
1.1 Viral Infections in Immunocompromised Patients 9
1.2 CMV 9
1.3 Epstein-Barr Virus 9
1.4 Adenovirus 10
1.5 Adoptive Immunotherapy with virus-specific T cells 10
1.6 Limitations of donor-derived virus specific VSTs 15
1.7 Most closely matched allogeneic virus-specific VSTs 15
1.8 Risks of Administering Virus specific VSTs 17
2 STUDY DESIGN 19
2.1 Primary Objective 19
2.2 Rationale for Study Design 19
2.3 Eligibility 20
2.4 Treatment Plan 25
2.5 Risks and Toxicities 29
3 STUDY ENDPOINTS 32
3.1 Primary Endpoints 32
3.2 Secondary Endpoints 34
4 PATIENT ENROLLMENT AND EVALUATION 36
4.1 Enrollment 36
4.2 Study Monitoring 37
4.3 Off Treatment & Off Study Criteria 41
5 STATISTICAL CONSIDERATIONS 41
5.1 Study Design Synopsis 41
5.2 Analysis of treatment feasibility 42
5.3 Analysis treatment efficacy 42
5.4 Monitoring of patients safety 42
5.5 Data Analysis 43
6 REPORTING REQUIREMENTS 44
6.1 Registration 44
6.2 Drug Toxicity and/or Adverse Reactions 44
6.3 Safety Analysis of Adverse Events Data 44
6.4 Adverse Event 44
6.5 Serious Adverse Event 45
6.6 Unexpected Adverse Event 45
6.7 Other Adverse Events 45
6.8 Relationship to Treatment 45
6.9 Severity Assessment 46
7 STUDY INTERPRETATION 46
7.1 Aim to collect information 46
7.2 Records to be Kept 47
7.3 Reporting Requirements 47
7.4 Adverse Event Reporting 48
7.5 Reporting to the IRB 48
7.6 Pregnancy Reporting 48
7.7 IND Annual Report to the FDA 48
7.8 Final Report 49

8 CLINICAL TRIAL OVERSIGHT AND MONITORING 49
8.1 Safety monitoring 49
8.2 Data Safety Monitoring Committee (DSMC) 49
8.3 Study Monitoring 50
Appendix I: CHRONIC GVHD DEFINITIONS & SCORING TABLE 51
Appendix II: ADVERSE EVENT Management FOLLOWING VST Infusions 52

PROTOCOL SYNOPSIS

Antiviral Cellular Therapy for Enhancing T-cell Reconstitution Following Hematopoietic Stem Cell Transplantation (ACES)

Study Design: The primary purpose of the study is to evaluate whether most closely HLA-matched multivirus-specific T cell lines obtained from a bank of allogeneic virus-specific T cell lines (VSTs) have antiviral activity against three viruses: EBV, CMV and adenovirus.

Reconstitution of anti-viral immunity by donor-derived VSTs has shown promise in preventing and treating infections associated with CMV, EBV and adenovirus post-transplant. However, the time required to prepare patient-specific products and lack of virus-specific memory T cells in cord blood and seronegative donors, limits their value. An alternative is to use banked partially HLA-matched allogeneic VSTs. A prior phase II study at Baylor College of Medicine using trivirus-specific VSTs generated using monocytes and EBV-transformed B cells gene-modified with a clinical grade adenoviral vector expressing CMV-pp65 to activate and expand specific T cells showed the feasibility, safety and activity of this approach for the treatment of refractory CMV, EBV and Adenovirus infections. More recent protocols utilizing synthetic viral peptide pools allow ex vivo expansion of T-cells targeting multiple viral antigens in 10-12 days without use of viral transduction.

In this trial, we will evaluate whether partially-HLA matched allogeneic multivirus-specific VSTs, activated using overlapping peptide libraries spanning immunogenic antigens from CMV, adenovirus and EBV, will be safe and produce anti-viral effects in immunodeficient recipients infected with one of more of the targeted viruses that are persistent despite conventional anti-viral therapy.

This study will evaluate safety and efficacy of partially-matched VST therapy in A) patients who have persistent viral infections in the post-HSCT period, and B) patients with primary immunodeficiency conditions who have persistent viral infections and have not undergone HSCT.

The study agent will be assessed for safety (stopping rules defined) and antiviral activity.

Primary Objective: The primary objective of this phase I/II study is to determine the feasibility, safety, and antiviral efficacy of administering partially HLA-matched VSTs to mediate antiviral activity in HSCT recipients with persistent viral reactivations or infections. Primary endpoints would include: treatment feasibility (based on the ability to identify partially HLA matched VST products for eligible

patients), incidence of toxicities including infusion-related toxicities, development of graft versus host disease, secondary graft failure, and primary graft failure for patients with primary immunodeficiency disorder (PID) who undergo HSCT within 12 months of VST infusion, and improvements in viral copy numbers in body fluids as well as clinical symptoms attributed to the targeted viral infection(s).

Secondary Objectives: Secondary objectives are to determine: effects of partially HLA-matched VST infusion on viral load and viral reactivations within 12 months, reconstitution of antiviral immunity, persistence of infused partially HLA-matched VSTs. Additional endpoints include correlation of HLA matching with clinical response and overall survival at 6 and 12 months.

Eligibility: Patients will be eligible as follows:

1) **Patients must meet one of the following criteria:**

A. Patients following any type of allogeneic hematopoietic stem cell transplant,

OR

B. Patients with a diagnosis of a form of primary immunodeficiency disorder (PID), based on clinical diagnosis with supporting laboratory and historical evidence, which can include family history, genetic sequencing results, and/or immunologic laboratory studies, without history of hematopoietic stem cell transplantation,

2) Patients must be ≤25 years old at the time of enrollment.

3) Patients must have CMV, adenovirus and/or EBV infection persistent despite standard therapy (as defined in section 2.3).

4) Patients who have undergone HSCT must have stable donor chimerism at the time of VST infusion. Stability would be defined as:

   a. >95% donor chimerism in CD33 or whole blood chimerism, OR
   b. >90% donor chimerism (CD33 or whole blood) with <5% change between subsequent tests separated by at least 1 week.

5) If patients are receiving steroids for treatment of Graft versus Host Disease (GVHD) or for other reasons, dosage must have been tapered to 0.5 mg/kg/day of Prednisone (or equivalent) prior to study enrollment.

6) For patients who received ATG, Campath, or other monoclonal antibodies targeting T-cells: a minimum of 5 half-lives or 28 days (whichever is shorter) must have elapsed.

7) Patients may not have received Janus kinase (JAK) inhibitors in the 3 days prior to VST infusion.

8) Patients must not have other uncontrolled infections (as defined in section 2.3.2), nor uncontrolled relapse of a malignancy (if applicable).

9) For patients who have received immune checkpoint inhibitors, 3 half-lives of the medication must have elapsed.

10) For patients with SCID who undergo α/β TCR depleted HSCT, at least 100 days must have elapsed after HSCT prior to VST infusion.

Treatment Description: The treatment schedule is as follows:

Patients will receive 2 x 10^7 partially HLA-matched VSTs/m^2 as a single infusion. In the rare case where insufficient banked cell product is available, a lower number of cells may be infused after discussion with the principal investigator, patient and/or guardian and the treatment team. Patients who have a partial response (>1 log decrease in viral load without clearance) or no response and do not have treatment-related dose-limiting toxicities are eligible to receive up to 3 additional doses from day 30 after the initial infusion and at 2 weekly intervals thereafter. The viral load of the virus (or viruses) that patients are initially treated for must be monitored by viral PCR. GVHD scores will be recorded at the intervals defined.

Accrual Objective: A maximum of 60 patients will receive at least one infusion of VSTs.

Accrual Period: The estimated accrual period is 36 months.

Study Duration: Patients will be followed for toxicity and for acute GVHD for 30 days; anti-viral responses for up to 3 months, and long term follow up including chronic GVHD for 12 months following the final VST infusion.

STUDY SCHEMA

Aim: To determine whether partially HLA-matched VSTs are safe and have antiviral activity against EBV, CMV and Adenovirus

Inclusion Criteria	Exclusion Criteria
1)<br>A. Received prior myeloablative, non-myeloablative, or unconditioned allogeneic hematopoietic stem cell transplant using either bone marrow, single/double cord blood and/or PBSC; with stable chimerism (defined as >95% donor chimerism [CD33 or whole blood chimerism], OR >90% donor chimerism [CD33 or whole blood] with <5% change between subsequent tests separated by at least 1 week).<br><br>OR<br><br>B. Diagnosis of a form of Primary Immunodeficiency Disorder (PID) based on prior clinical and laboratory evaluation, without history of hematopoietic stem cell transplantation.<br><br>2) Age ≤ 25 years<br><br>3) Cells administered as treatment of persistent infection(s) for EBV, CMV or Adenovirus despite standard therapy, or for patients who are unable to tolerate standard therapy due to toxicity. Patients with infections with multiple targeted viruses are also eligible to enroll.<br>4) Steroids less than or equal to 0.5 mg/kg/day prednisone<br>5) Negative pregnancy test (if female of childbearing potential after reduced intensity conditioning)<br>6) Patient or parent/guardian capable of providing informed consent.	1) Received Anti-thymocyte globulin (ATG), Campath, Tociluzimab, Basiliximab, Brentuximab, or other monoclonal antibodies impacting T-cell survival within previous 28 days (or <5 medication half-lives, if shorter).<br>2) Received donor lymphocyte infusion or other experimental cellular therapies in last 28 days.<br>3) Received ruxolitinib or other JAK inhibitors in the previous 3 days.<br>4) Patients with other uncontrolled infections.<br>5) Active and uncontrolled relapse of malignancy (if applicable).<br>6) Patients who have received immune checkpoint inhibitors with <3 medication half-lives elapsed.<br>7) Patients with severe combined immunodeficiency who are less than 100 days post α/β TCR depleted HSCT.

Up to 3 additional doses can be administered from day 30 after the initial infusion if there is a partial response or stable disease, or if the patient receives immunosuppressive therapy that would compromise VST activity after the first infusion (such as corticosteroids), and if the patient has not experienced toxicities related to the study product and meets eligibility criteria for subsequent infusions. Subsequent doses should be at least 14 days apart.

Primary endpoints:

-Feasibility of finding a suitable line for infusion
-Safety: including acute GVHD (grades III-IV), grades 3-5 infusion-related adverse events, hematologic toxicities including primary or secondary graft failure, or grades 4-5 non-hematological adverse events occurring within 30 days of the last VST dose that are not due to pre-existing infection(s), malignancy (if applicable), or other pre-existing co-morbidities, as defined by the NCI Common Terminology Criteria for Adverse Events (CTCAE), Version 4.03.
-Antiviral responses within 30 days following the first VST infusion based on viral copy numbers and clinical signs/symptoms.

Secondary Endpoints

-Reconstitution of antiviral immunity in the 3 months following VST infusion
-Persistence of partially HLA-matched VSTs at 1 and 3 months.
-Overall survival at 6 months and 12 months
-Viral reactivations within 3 months of VST infusion.
-Chronic GVHD at 6 months and 12 months
-Correlation of overall HLA matching with antiviral responses and VST persistence at 3 months.
-Correlation of choosing a VST product with confirmed antiviral HLA restrictions with antiviral responses at 30 days following VST infusion.

1 BACKGROUND AND RATIONALE

1.1 Viral Infections in Immunocompromised Patients

T-cell immunity is critical for control of viral infections.[1] Thus, in immunocompromised patients (e.g. those who have undergone hematopoietic stem cell transplantation (HSCT), or patients with moderate to severe primary immunodeficiency disorders) viral infections are a major cause of morbidity and mortality.[2-10] Risk for infection is dictated by the degree of tissue mismatch between donor and recipient, and the resultant degree of immunosuppression, and by the immune status of the donor. Reactivation of latent viruses, such as cytomegalovirus (CMV) and Epstein-Barr virus (EBV), are common and often cause symptomatic disease.[7,11,12] Respiratory / enteral viruses, such as adenovirus, also frequently cause infection.[13] Antiviral pharmacologic agents are only effective against some of these viruses; their use is costly, and associated with significant toxicities and the outgrowth of drug-resistant mutants.[14-16]

Chronic viral illnesses are a common presenting feature of many forms of primary immunodeficiency disorders (PID), and studies have shown that survival of HSCT is profoundly impacted by the patient's pre-transplant disease status.[17] Though some forms of PID are amenable to HSCT without requiring conditioning chemotherapy, many forms require a variable degree of pre-conditioning to ensure that stable engraftment of the donor cells is achieved.[18,19] The administration of cytotoxic chemotherapy used in the conditioning regimens can however increase the risk for regimen related toxicity and for some patients (especially those with active viral infections) this risk is particularly high, leading to elevated treatment related mortality rates. For these reasons, many such patients are not even considered candidates for HSCT or are delayed getting to HSCT and ultimately succumb to infection before they can receive the transplant.

As delay in recovery of virus-specific cellular immune response is clearly associated with viral reactivation and disease in immunocompromised patients, cellular immunotherapy to restore viral-specific immunity is an attractive option that has already been successfully used to target a number of viruses.[20-22]

1.2 CMV

Cytomegalovirus (CMV) is a latent beta-herpesvirus that usually causes an asymptomatic infection in immunocompetent individuals.[23] It persists in approximately 70% of healthy adults and replicates in epithelial cells, fibroblasts and monocytes. Reactivation of CMV in the stem cell recipient can result in significant morbidity and mortality, with clinical manifestations including interstitial pneumonitis, gastroenteritis, fevers, hepatitis, encephalitis and retinitis.[11,24] Cell-mediated immunity is considered the most important factor in controlling CMV infection and CMV-specific $CD4^+$ and $CD8^+$ lymphocytes play an important role in immune protection from both primary infection and subsequent reactivations.[25] The most frequently used drugs for prophylactic or preemptive therapy are ganciclovir and foscarnet. These drugs have been successful in reducing mortality associated with CMV disease and in preventing early CMV disease in combination with intravenous immune globulin, but both have major side effects, including neutropenia, thrombocytopenia, and nephrotoxicity.[26]

1.3 Epstein-Barr Virus

Epstein-Barr virus (EBV) is a gamma-herpesvirus that infects more than 95% of the world's population. Primary infection usually produces a mild self-limiting disease, which is followed by

latent infection in B cells and productive replication in B cells and mucosal epithelium. There are at least four types of viral latency, which differ in their viral antigen expression patterns.[27] In immunocompromised hosts, outgrowth of B cells expressing Type 3 latency (expression of all seven latency associated proteins)may lead to the development of post-transplant lymphoproliferative disease (PTLD).[7] The overall incidence of PTLD after HSCT is approximately 1%, but the incidence is increased in recipients with an underlying diagnosis of immunodeficiency and for recipients of stem cells from unrelated or human-leukocyte-antigen (HLA)-mismatched donors who receive grafts that are selectively depleted of T cells to prevent graft-versus-host disease (GVHD).[12,28] PTLD is highly susceptible to control by virus-specific T cells.[29]

Few small molecule drugs have any effect on B cells already transformed by EBV and while nucleoside analogs including ganciclovir inhibit the viral explicative cycle, their clinical benefit is questionable at best. Conventional chemotherapy, while effective in some cases is associated with significant toxicity with high treatment related mortality in the HSCT patient population. One option for prophylaxis and treatment of PTLD after HSCT is rituximab, a monoclonal antibody against the B cell phenotypic antigen, CD20.[30] However, not all patients respond and rituximab depletes normal B-cells for more than 6 months, which can be problematic in a patient that is already immunosuppressed.[31]

1.4 Adenovirus

Adenovirus is a non-enveloped lytic DNA virus. Humans are susceptible to infection with 51 serotypes forming six distinct adenovirus species (A to F), which differ in their tissue specificity and virulence.[9] Although causing acute infections, adenoviruses may persist for many months after resolution of disease and therefore are frequently carried undetected into the transplant by donor or recipient.[32] While acute infection is rarely fatal in healthy adults, it is a significant cause of morbidity and mortality in immunocompromised individuals, in whom it may produce pneumonia, hemorrhagic cystitis, nephritis, colitis, hepatitis, and encephalitis. Adenovirus has a particularly high incidence after pediatric HSCT. Several reports have shown that clearance of adenovirus infection is associated with detection of adenovirus-specific T cells and recovery is significantly delayed in recipients of matched unrelated donor and haploidentical transplant who receive intensive immunosuppression such as alemtuzumab.[8,33] The most frequently used drug for disease treatment is Cidofovir, but the associated nephrotoxicity is a major concern.[34,35] The prodrug brincidofovir has also been used with some evidence of efficacy.[36] However, resistance mutations to cidofovir and brincidofovir has been described.[37]

1.5 Adoptive Immunotherapy with virus-specific T cells

Since recovery of virus-specific T cells is clearly associated with protection from infection with each of these viruses, adoptive immunotherapy to decrease the time to immune reconstitution is an attractive approach.[38,39] Virus-specific T cells generated by repeated stimulation with antigen presenting cells (APCs) expressing viral antigens have been evaluated in clinical trials to prevent and treat viral infections in immunocompromised hosts.[22,40-46] This approach eliminates alloreactive T cells.

There are several considerations in developing protocols for generating VSTs ex vivo. Knowledge of the immunodominant antigens that induce protective T cells specific for the targeted virus is required and a delivery system to transfer the antigen to effective APCs must be identified. The APC must be autologous and express major histocompatibility complex (MHC) antigens presenting relevant virus-derived peptides as well as co-stimulatory molecules

sufficient to induce T cell activation and expansion. These reagents all need to be suitable for GMP manufacturing, which limits the use of some types and sources of antigen.

1.5.1 Therapy with VSTs specific for CMV

In the first study evaluating whether adoptively-transferred T cells could reconstitute anti-viral immunity, CMV-specific T-cell clones were derived from sibling donors after stimulation with autologous fibroblasts pulsed with CMV.[47] There were no adverse effects and CMV-specific immune responses were reconstituted, with none of the patients developing CMV disease or late recurrence. In another prophylaxis study, Peggs *et al*. generated CMV-specific CD4+ and CD8+ T cells by stimulation of peripheral blood mononuclear cells (PBMC) with dendritic cells pulsed with CMV antigens derived from a CMV-infected human lung fibroblast cell line.[48] Small doses of VSTs were able to reconstitute immunity with considerable *in vivo* expansion of CMV-specific VSTs. To avoid the use of live CMV during T cell manufacture, a more recent study stimulated VSTs with dendritic cells pulsed with the HLA-A2 restricted peptide NLV derived from the cytomegalovirus-pp65 protein.[49] While this approach also appeared effective, a concern is the restricted specificity of the infused VSTs, since targeting a single epitope may allow escape variants and limits the study to patients who are HLA A2-positive.

CMV-specific VSTs have also been used therapeutically in patients with CMV infection that has persisted or recurred despite prolonged antiviral medication.[50] The results were encouraging, with suppression of viral reactivation in 6 of 7 subjects. In this study, the source of antigen was a CMV lysate, which has the advantage of producing a broad immune response, but which is unsuitable for Phase III studies because of the risk of infection from live virus in the lysate.

The VST cell therapies described above all employed methods of T cell production that require prolonged periods of activation and expansion in specialized GMP facilities with significant regulatory support. These requirements reduce the practicality of adoptive immunotherapy since VST lines must be made long in advance of disease and few centers have the facilities or infrastructure required for this type of cell processing. To overcome these problems, two direct T cell selection approaches have been used to isolate CMV-specific T cells from peripheral blood without the requirement for ex vivo expansion. The first approach is the interferon (IFN)γ-capture assay, which isolates IFNγ-secreting T cells after antigen exposure.[51] Another approach is using a MHC-peptide-multimer-construct, e.g. streptamer selection.[52]

1.5.1.1 IFNγ-capture of CMV-specific T cells

Feuchtinger and colleagues used IFNγ-captured CMV-specific T cells to treat chemorefractory CMV disease or reactivation after allogeneic HSCT.[53] After short-term stimulation and selection a mean of 21.3×10^3 CD3+ cells per kg was infused to 18 patients, without acute side effects and only a single case of GVHD. In 15 of 18 infused patients clearance of CMV viremia or a significant reduction (>1 log) of viral load was associated with the adoptive transfer. Peggs *et al.* also reported the clinical results associated with the adoptive transfer of IFNγ-captured CMV-specific T cells.[54] In this prophylaxis/preemptive treatment study, pp65 protein or peptide pools were used to stimulate T cells. Post-selection a median of 2840 $CD4^+$ and 630 $CD8^+$ CMV-specific T cells/kg were infused early (median day 35) post-transplant and expansion of both populations was detected *in vivo*. However, unlike the Feuchtinger study, acute GVHD was observed in 8 of 18 patients, although this was mild in most cases with only 2 patients requiring systemic steroid therapy. More recently, Creidy *et al*. treated 15 patients with IFNγ selected T-cells for treatment of refractory infection with CMV or Adv following HSCT.[55] Complete responses were obtained in 3 patients with CMV, and 3 patients with AdV infection, in spite of

very low VST doses (1-20x10^3 $CD3^+$/$IFN\gamma^+$ cells/kg). Of the 9 patients who did not respond to VST therapy, 8 of 9 died, with most due to progressive viral disease. In contrast, 5 of 6 responders survived, with one late death due to adenoviral pneumonia in a patient who received CMV-specific VSTs. The authors reported a single case of acute GVHD (grade III) and one case of chronic GVHD in the subjects.

1.5.1.2 Multimer-based isolation of CMV-specific T cells

Multimer techniques such as tetramers have also been developed for the direct selection of antigen-specific T cells. For example Cobbold and colleagues performed a clinical trial using tetramer selection of CMV peptide-specific T cells directly from peripheral blood.[56] Cells were infused to patients within 4 hours of selection. Although exclusively $CD8^+$ T cells were infused, they expanded by several logs after infusion, clearing infection in 8/9 cases.[29] More recently, streptamers have been used to clinically select distinct T cell populations for adoptive transfer. Streptamers, because of the biotin-streptavidin interaction, detach from T cells over time and are thus not considered to be drugs, but rather adjuvant. This technical feature distinguishes streptamers from other multimers and facilitates their clinical use. Schmitt and colleagues recently reported the first clinical experience of streptamer-selected CMV-specific T cells.[57] After a single infusion of 2.2×10^5 HLA-B7+/CMV pp65-specific CD8+ T cells/kg (purity of 97%), the frequency of CMV-specific T cells increased dramatically in vivo from 0% prior to infusion to a maximum of 27.1% of all T cells. These T cells were confirmed to be donor in origin by analysis of donor chimerism through single-tandem repeats and Vβ-chain typing. Clinically, the T cell infusion resulted in CMV clearance without alloreactivity.

Though both approaches have been associated with clinical benefit, the multimer strategy is limited by the HLA restriction of antigen recognition, the availability of clinical grade multimers and lack of HLA class II multimers. Additionally, both the multimer and IFNγ approaches require large numbers of starting peripheral blood material, which may not always be readily available (e.g. from an unrelated donor).

1.5.2 Therapy with VSTs specific for EBV

EBV-transformed lymphoblastoid cell lines (LCLs) generated by infecting peripheral blood B cells with a laboratory strain of EBV can be readily prepared from any donor, they have been used as APCs in clinical studies evaluating EBV specific VSTs.[43,58-61] We have shown that adoptively transferred EBV-specific VSTs can survive for >10 years, expand up to 2-4 logs after infusion, and reduce the high virus load that is observed in about 20% of patients. In a recent review of three studies targeting high-risk patient populations, none of 101 patients who received EBV VSTs as prophylaxis developed PTLD.[40] Of 13 patients with active PTLD at the time of infusion, donor-derived EBV-specific VST lines induced remission in 11, while in one of the non-responders, tumor virus had deleted the immunodominant epitopes in one of the viral antigens that were the targets of the infused effector T cells. Numerous other studies have confirmed the activity of *ex vivo* expanded EBV-specific VSTs post transplant.[61,62]

Rapid selection techniques have also been used to directly isolate EBV-specific populations for infusion. Indeed, Moosmann and colleagues isolated IFNγ secreting cells following exposure to 23 class I and II peptides derived from 11 EBV antigens, which were infused into 6 patients with EBV-PTLD.[63] While three patients responded, three with more advanced disease progressed. Whether these patients would have responded had they received higher numbers of effector T-cells remains an important question for future development. Icheva and colleagues specifically

targeted EBNA1 and pulsed APCs with either whole EBNA1 protein or EBNA1 overlapping peptide pools and then selected responding T cells by IFNγ capture.[64] Ten patients with PTLD were treated and 7 had clinical responses. No significant toxicities were seen in these studies. Finally, Uhlin and colleagues isolated, using multimers, HLA A2-restricted T cells specific for epitopes in two EBV antigens from a haploidentical parent to treat EBV PTLD in a cord blood transplant recipient.[65] Post-infusion a small number of these directly selected cells expanded in vivo and produced a complete clinical response. At 12 months after transplant the EBV-PTLD recurred, but a second multimer selection and infusion induced a second response.

Of note, there has been a published case of a pediatric patient with PID who developed EBV-associated primary CNS lymphoma prior to HSCT, and was treated with third-party EBV-specific CTL with successful cure of the lymphoproliferative disorder, and went on to successful cord blood transplantation.[66] This patient received multiple infusions (2 to 7 total) of partially matched EBV-CTLs at a dose of 2x10E6/kg/dose (comparable to our standard third-party mCTL dose of 2x10E7/m^2).

1.5.3 Therapy with VSTs specific for Adenovirus.

Feuchtinger *et al*. treated patients with adenovirus infection using $CD4^+$ and $CD8^+$ adenovirus-specific T cells isolated from the donor after a short *in vitro* stimulation with adenovirus viral antigen followed by selection of IFNγ-secreting cells.[67] Small numbers of adenovirus-specific donor T cells were infused into nine children with systemic adenovirus infection after HSCT. Adenovirus specific immune responses were detected in five of six evaluable patients, associated with a sustained decrease in viral load and clearance of infection.

1.5.4 Trivirus-Specific Donor-Derived T Cells

Most studies with VSTs have only targeted single viruses. To broaden the specificity of single VST lines to include three of the most common viral pathogens of stem cell recipients (EBV, CMV, and adenovirus) we previously generated virus-specific VSTs using a recombinant adenoviral vector encoding the CMV antigen pp65.[42] This method reliably produced VSTs with cytotoxic function specific for all three viruses in a process requiring 8-12 weeks for manufacture. When we infused these cells to 14 stem cell recipients in a Phase I prophylaxis study we observed recovery of immunity to CMV and EBV in all patients, but an increase in adenovirus-specific T cells was only seen in patients who had evidence of adenovirus infection pre-infusion. A follow-up study in which the frequency of adenovirus-specific T cells was increased in the infused VSTs produced similar results, thus highlighting the importance of endogenous antigen to promote the expansion of infused T cells *in vivo*.[41] Nevertheless, all patients in both clinical trials with pre-infusion CMV, adenovirus, or EBV infection or reactivation were able to clear the infection, including one patient with severe adenoviral pneumonia requiring ventilatory support.

More recently, several groups have sought to simplify and accelerate T-cell expansion with minimal cell handling, while ensuring that T-cell specificity and function is maintained. The first step was to replace EBV-LCLs and adenovirus vectors as a source of antigen and APC. To eliminate the six weeks required for EBV-LCL manufacture, we investigated two alternate sources of biohazard-free antigen sources (plasmids and overlapping peptide libraries) as well as dendritic cells (DCs) or APCs present in peripheral blood to activate virus-specific T cells.[68,69] To first assess whether plasmids could substitute for EBV-LCL/adenovectors we generate clinical grade plasmids encoding immunogenic EBV (EBNA1, LMP2, BZLF1), CMV (IE1, pp65) and adenovirus (Hexon, Penton) antigens and used DCs nucleofected with these plasmids to

generate trivirus-specific T cells, which were expanded *in vivo* in the presence of the cytokines IL4+7 in a G-Rex device for 10 days before being cryopreserved for clinical use. To test the *in vivo* activity of these cells we have recently completed a phase I/II treatment study, achieving a response rate of 80% in patients with single or multiple active infections, without adverse effects.[70] Thus, VSTs recognizing multiple antigens from 3 viruses can produce clinically relevant effects in vivo.

1.5.5 Generation of multivirus-directed VSTs by direct stimulation of PBMCs with clinical grade pepmixes.

Gerdemann *et al.* developed a 10-day GMP-compliant T cell production process to generate T cell lines with simultaneous specificity for 3 viruses (Adv, EBV, and CMV).[68] To activate PBMCs we expose PBMCs directly to a mastermix of pepmixes (peptide libraries of 15mer peptides overlapping by 11 amino acids) spanning immunogenic antigens derived from each virus; -adenovirus (Hexon and Penton), CMV (IE1 and pp65), and EBV (LMP2, EBNA1, BZLF1). After activation cells are transferred to a G-Rex10 device for 9-11 days and cultured in media containing IL4 and IL7 to achieve maximal expansion of antigen-specific T cells without carry-over of alloreactive T cells. From starting cell numbers of 15×10^6 PBMCs/G-Rex we were reproducibly able to produce 1-1.2 x 10^8 antigen-specific VSTs in the stated timeframe. These multivirus VST lines are polyclonal and polyfunctional, with activity against the stimulating viruses detectable in both the $CD4^+$ and the $CD8^+$ T cell fractions.

1.5.5.1 Clinical activity of donor-derived multivirus-directed VSTs

Bollard *et al*. have evaluated the clinical utility of multivirus-specific VSTs in recipients of matched related, matched unrelated, or haploidentical donor transplants.[71-73] To date, 40 clinical-grade multivirus-directed VSTs have been generated from donor PBMCs at Children's National Hospital. These lines are polyclonal, and are comprised of both $CD4^+$ and $CD8^+$ subsets, and effector memory, central memory (CD45RO+ CCR7+ CD62L+) and stem cell memory (CD45RA+ CCR7+ CD95+) subsets. Antiviral specificity of our existing products has depended on the prior viral exposure of the cell donor; 36/38 tested lines had activity against CMV, 36/38 against Adenovirus, and 34/38 against EBV. None of the products reacted against MHC-mismatched lymphoblast target cells by ^{51}Cr release assay, indicating lack of alloreactive potential in these rapidly generated lines.

At Baylor College of Medicine (BCM), Bollard *et al.* administered multivirus-specific donor-derived VSTs to 3 allogeneic HSCT recipients in a dose escalation study all on DL1 ($5 \times 10^6/m^2$). There were no immediate infusion toxicities, and no *de novo* acute GvHD, demonstrating the *in vivo* safety of these mVST. Further, antiviral efficacy has been observed in 1 patient with refractory CMV. In addition, in a recently published paper from BCM, VSTs were manufactured with a similar methodology, but targeting 5 viruses instead of 3, with additional specificity to BK virus and HHV6.[22] In that study 10 patients were treated with 4 on DL1 (5x10e6/m2), 4 on DL2 (1x10e7/m2) and 2 on DL3 (2x10e7/m2) and again saw no immediate infusion toxicities, and no *de novo* acute GvHD, demonstrating the *in vivo* safety of these mVST. Three patients received the cells as viral prophylaxis (days 38-43 post-HSCT) and none developed viral infections at up to 3 months post-treatment. The other 7 patients received the cells as treatment for one or more active infections between days 59-139 post-HSCT. Based on viral load measurements by day 42 post-infusion, the VSTs were successful in controlling active infections with CMV (1 complete (CR) and 1 partial response (PR)), EBV (2 CRs, including a case of frank PTLD); Adv (1 CR); HHV6 (1 CR); and BK (3 CR, 1 PR, 1NR). Of note, 3 BK virus responders had tissue disease with severe hemorrhagic cystitis and all had marked improvement or disappearance of hematuria following infusion. One patient subsequently had an episode of transient but severe

bladder pain in association with inflammation seen on cytoscopy coincident with a 6 log fall in urine BK viral load. Our only non-responder was a patient with BK infection whose line lacked activity for this virus, likely reflecting the serostatus of the donor. In addition, 3 patients subsequently reactivated other viruses than those for which they were initially treated, but all cleared these infections by week 12, without the requirement for additional cell infusions (CMV: 1CR; EBV: 1CR; BK: 1CR; HHV6: 1CR). Finally, 1 patient received multivirus specific VSTs under a single patient protocol as an emergency treatment for widespread and bulky rituximab-resistant EBV-PTLD. Post VST treatment there was an immediate decline in the patient's EBV viral load with complete and sustained resolution of PTLD, coincident with an increase in circulating EBV-specific T cells. However, the profound anti-tumor activity mediated by the rapidly-expanding EBV-directed T cells also produced a transient systemic inflammatory response syndrome, which was controlled with steroids and anti-TNFR antibody, with no long term adverse effects.

Many patients with PID have received multivirus-specific T-cells in previous trials.[22,41,46,74] In a recently published case series, 36 patients with PID received VST therapy at 4 institutions.[71] Of these 36 patients, 13 received T-cells targeting 3-5 viruses, 10 of which were derived from their HSCT donors, and 3 of which were derived from third-party donors and were partially-HLA matched. Of these patients, 10 of 13 were either protected from infection, or cleared all existing infections with the targeted viruses, with minimal graft versus host disease (grade I-II) in 2 patients in the 45 days following T-cell infusion, which was treatment responsive.

Thus, infusion of donor-derived, multivirus specific VSTs generated with clinical grade pepmixes and infused either prophylactically or as treatment for one or more viral infections has been safe and is associated with the appearance of virus-reactive T cells in peripheral blood that have been able to control infection with up to 5 targeted viruses.

1.6 Limitations of donor-derived virus specific VSTs

Despite the successful use of donor-derived multivirus-directed VSTs in these trials, there are some limitations to this approach. One problem is the rapid expansion protocol for generating virus-specific VST products can only be generated from donors who had previously been exposed to a virus has not been successful from seronegative donors or cord blood. New methodologies have allowed generation of VST from cord blood, though production requires 6-8 weeks, and is not available at most centers.[75,76] There is increasing evidence over the last years that the use of younger donors is associated with improved engraftment, decreased relapse rates and improvements in overall and event-free survival in HSCT patients.[77] However younger donors are also more likely to be seronegative for many targeted viruses, which along with the expanding use of cord blood transplants increases the number of patients for which donor-derived VSTs cannot be generated. Another problem is that despite the significant decrease in the amount of time it takes to manufacture virus-specific VSTs with our pepmix-based method (decrease from ~3 months to just 10 days), this time frame is still too long for patients with more fulminant viral disease.

1.7 Most closely matched allogeneic virus-specific VSTs

An alternative approach that bypasses the need to grow VSTs for individual patients is to bank closely HLA-matched allogeneic VSTs that could be available as an "off the shelf" product.[78] A concern with this approach is that the *in vivo* persistence of a mismatched product may be suboptimal after administration, as the recipient may generate an immune response to the non-shared HLA antigens. However, a number of studies have demonstrated the feasibility of this

approach as a salvage therapy and reported clinical responses in the patients with EBV lymphoma arising after HSCT or solid organ transplant.[46,79-82] In the first and largest multicenter study, Haque *et al.* used banked polyclonal EBV-specific T-cell lines to treat EBV-PTLD after HSCT or solid organ transplantation and reported an overall response rate of 52% at 6 months. Similar results have been reported from Memorial Sloan Kettering Cancer Center with four of five PTLD patients achieving CR in response to third party EBV-specific T-cells. Of note none of these studies report an increased risk of GVHD.

More recently the group at BCM (with Dr. Bollard) applied this approach to treat patients with refractory CMV, Adenovirus and EBV infections that are unresponsive to standard antiviral therapy post allogeneic HSCT.[83] Products were generated using adenovector-pp65 transduced monocytes and EBV-LCL and were either retained from our prior donor-specific clinical study, or were newly generated from donors with known antiviral activity, including HLA-homozygous donors identified by the National Marrow Donor Program. A total of 32 lines were produced and characterized, 18 of which were administered to the 50 study patients. The selection of lines for infusion was based on the specificity of the line for the target virus through a shared HLA allele, as well as the overall level of HLA match.

This study was open to allogeneic HSCT recipients with CMV, AdV, or EBV infection that had persisted for at least 7 days despite standard therapy. Patients who had a suitable VST line received an intravenous infusion of up to 2×10^7 VSTs/m^2 and were eligible for additional infusions at intervals of at least 2 weeks, in the event of a partial response (PR). Of the 50 patients who received VST infusions 23 received VSTs for persistent CMV, 18 for persistent adenovirus, and 9 for refractory EBV-PTLD. Five of the 50 treated patients withdrew from the study or died of their underlying disease.

From the bank of 32 VST lines a suitable line was identified for 90% of the screened patients within 24 hours. Of the 50 patients who were treated with these VSTs, 74.0% had a CR or PR (73.9% for CMV, 77.8% for AdV, and 66.7% for EBV), including responses in 6 of 9 patients with refractory EBV-PTLD, and most of these responses (89%) were durable. Of non-responders, the majority (10 of 12) died of the ongoing viral infection. By contrast, 8 patients in the study for whom a line was not available and who continued with “standard therapy” demonstrated a 13% response rate, and 6 (75%) died of viral disease.

A small number of patients with PID have been previous treated with VST in the pre-HSCT time period in an effort to control active viral infections prior to transplantation. Two of these cases have been previous published.[66,71] One patient with CTPS1 deficiency received several doses of EBV-specific T-cells for EBV-associated CNS lymphoma, immediately followed by cord blood transplantation. She had complete clearance of the lymphoma and engrafted without incident. Another patient with ADA-SCID received a 5/10 matched third-party product for CMV and EBV. She unfortunately had progressive viral disease, but did not have any adverse reactions to the VST infusion. One patient with RAG1 SCID was treated by the Bollard group for disseminated adenoviral infection which was refractory to cidofovir and brincidofovir (unpublished). He received two partially HLA matched VST products (3/10 and 4/10 matched), immediately followed by HSCT from a matched unrelated donor. In the three weeks after the second VST infusion, adenovirus rapidly cleared from all body fluid, and CD4$^+$ T-cells specific for adenovirus were detectable in his peripheral blood by IFNγ capture assay. TCRβ sequencing demonstrated that adenovirus-specific T-cells from the second VST infusion expanded in the patient’s blood in the three weeks following infusion. He had no adverse reactions or GVHD, and is currently alive and well.

1.8 Risks of Administering Virus specific VSTs

1.8.1 Risk of Administering Donor-derived VSTs

Cruz *et al*. recently reviewed the infusion-related adverse events (AE) following administration of ex vivo-expanded T cell products (antigen-specific cytotoxic T lymphocytes, allodepleted T cells, and genetically modified T cells) on investigational new drug (IND) studies at BCM.[84] From 1998 to 2008, they infused a total of 381 T cell products to 180 recipients, enrolled on 18 studies, receiving T cells targeting malignancies or post-transplant viral infections. There were no grade 3-4 infusion reactions during initial monitoring or 24-h follow-up. Twenty-four mild (grade 1-2) AE occurred in 21 infusions either during or immediately following infusion (up to 6 hrs), most commonly nausea and vomiting (41.6%), probably because of the dimethyl sulfoxidecryoprotectant, and hypotension (20.8%), attributable to diphenhydramine pre-medication. Twenty-two additional non-severe events were reported within 24 h of infusion, most commonly culture-negative fever, chills and nausea. An increased risk of AEs was associated with age [incidence rate ratio (IRR) 0.98; 95% confidence interval (CI) 0.96-1.00, P = 0.05], while an increased risk of immediate infusion-related events was higher in patients reporting allergies (IRR 2.72, 95% CI 1.00-7.40, P = 0.05); sex, disease type and T cell source (allogeneic or autologous) had no effect on frequency of adverse events.

Focusing specifically on VSTs, over 125 patients have been infused with EBV or multivirus VSTs in the donor-specific setting. The vast majority (123) occurred with no or minimal toxicity consisting mainly of localized swelling at sites of responsive disease. Two patients infused with donor-specific products developed syndromes consistent with systemic inflammatory response syndrome (SIRS).[43] The first patient was infused with EBV VSTs as treatment for bulky disease, including extensive pharyngeal disease. He had a vigorous inflammatory response with an immune infiltrate of genetically marked cells apparent on follow up biopsy. This response produced progressive airway obstruction and mucosal sloughing, ultimately requiring mechanical ventilation. He also had reversible cardiac impairment and fevers during this response. This patient subsequently made a full recovery and remains well more than 10 years later. He was treated in 1996 when cytokine panels were not available so SIRS could not be confirmed, but it seems likely. The second patient was infused with multivirus VSTs as treatment for rituximab-resistant EBV-PTLD, with bulky disease. Approximately two weeks after receiving the VSTs she developed a fever, became tachycardic, requiring fluid boluses and eventually required transfer to ICU with fever, tachycardia and hypotension for inotropic support. She required Dopamine and Norepinephrine to maintain her blood pressure and appeared to be in warm shock. The differential diagnosis of her treating physicians was sepsis or SIRS associated with VST therapy. She also had a diffuse erythematous rash consistent with drug reaction, infection or GVHD. As her blood cultures were negative and she continued to require inotropic support, was given empiric etanercept as well as methylprednisolone (1 mg/kg daily x2) and her symptoms resolved within a few hours. She remains well.

In other reported studies, none of the patients treated by Walter *et al*. with CMV VST developed GVHD.[47] In the cohort of patients treated by Peggs, MacKinnon *et al*., 3/13 patients developed mild (Grade I) GVHD; since immunosuppression had been withdrawn early in this study, it is unclear if this side effect was due to VST infusion.[54] Of current patients on protocols at Children's National Hospital, 3 of 22 patients who received multiviral VST derived from an HSCT donor developed acute GVHD after infusion. In all cases, GVHD was low grade (I-II) and treatment responsive without further viral disease.

1.8.2 Risk of 3rd party VSTs

In a study at Memorial Sloan Kettering Cancer Center, 5 patients received a median of 5 doses of third party virus specific VSTs for EBV after HSCT for EBV LPD, most at $1x10^6$ EBV-VST/kg/infusion, and all infusions were well tolerated.[82] Updated data from the study was reported at the American Society of Hematology meeting 2012, with 10 patients after HSCT enrolled in this study and among them one patient developed mild skin GVHD after infusion with 3rd party EBV-VSTs, but tolerated a subsequent infusion of EBV-VSTs from an alternate 3rd party donor, with no other incidences of GVHD reported.

In a recent BCM study of third party VSTs specific for three viruses (AdV, CMV and EBV) a total of 50 patients were infused with third party banked VSTs. All of the infusions were well tolerated. There were no immediate adverse effects, and despite the HLA disparity between the VSTs and recipients, de novo GVHD occurred in only 2 patients (grade I in each case). In the 8 patients in whom acute GVHD developed within 45 days of the first infusion (grade I in 6 patients, grade II in 1 patient, and grade III in 1 patient), 6 had a history of GVHD prior to receiving the VSTs. An additional patient had a flare of chronic skin GVHD. Two patients experienced transplant-associated microangiopathy, a complication that occurs in up to 10% of HSCT recipients, particularly in those receiving sirolimus, as were both of these patients. Only 1 patient had secondary graft failure, concomitant with leukemic relapse.[83]

Previous studies have also demonstrated that transfer of allogeneic T-cells specific for minor histocompatibility antigens (MHCA), including Y-chromosome (H-Y) antigens in gender mismatched transplants can result in alloreactions, which may include graft versus host disease or graft-versus-leukemia effects.[85-90] To date, secondary graft rejection after HSCT and third-party VST therapy has not been described in published reports, but this also may be theoretical risk. Previous studies have demonstrated that T-cell responses to MHCAs were fairly common in multiparous women.

In order to minimize the risk of infusing a VST product with alloreactive potential in studies of donor-derived VSTs, Bollard *et al*. have screened products for reactivity against MHC-mismatched PHA lymphoblasts as a release criteria.[41,42] However, there is no completely reliable *in vitro* assay for excluding the possibility of alloreactivity,[91] and performing such an assay in the current protocol would be difficult for two reasons. First, many recipients would not have residual pre-transplant lymphocytes available to make PHA blasts and their blood at the time of study eligibility determination would be of donor origin and therefore not a valid predictive target. Second, it would add 10 days to the release time which would adversely affect feasibility and perhaps outcome. We do not therefore propose this assay as a release criterion. However, all VST products will have been previously tested for alloreactivity with MHC mismatched targets as a condition of release for third-party usage. Furthermore, to reduce the risk of alloreactions related to donor sensitization minor histocompatibility antigens, third-party donor selection will utilize additional risk screening criteria in addition to standard blood or marrow donor criteria.

1.8.3. Risks of Administering CTLs to PID patients prior to HSCT

Allosensitization with donor-specific, anti-HLA antibodies has been described to cause graft rejection in recipients of solid organ transplants or HSCT for malignancy.[92] However, patients with severe forms of PID often have T-cell and humoral dysfunction that would impair or prevent allograft rejection. In patients with Severe Combined Immunodeficiency (SCID), this permits HSCT in the absence of preconditioning, resulting in the establishment of mixed chimerism.[6]

This is further permitted by the survival advantage imparted to donor T-cells in comparison to the dysfunctional or absent recipient T-cells. However, in some forms of PID, alloreactive T-cells and autoantibodies are formed due to immune dysregulation. These autoimmune features are the cause of Omenn's syndrome in certain forms of SCID, and and are also common in Wiskott-Aldrich syndrome and many other disorders, including dominant gain-of-function mutations in STAT1 and STAT3, and activated PI3-kinase-delta syndrome.[93-96] However, in spite of the relatively common occurrence of autoimmune phenomena, allosensitization is rare in patients with PID, and successful solid organ transplantation after HSCT have been reported in PID patients in spite of frequent need for blood products in this population.[97] Treatment of allosensitization with plasmapheresis and IVIG has been previously used successfully in transplantation. Parenthetically, most patients with PID are treated with immunoglobulin, albeit at replacement rather than immunomodulatory doses. Thus, the theoretical risk of allosensitization with VST therapy prior to HSCT is likely low. Additionally, by employing a strategy of minimizing HLA matches between the VST donor and the eventual HSCT donor (if identified) at all HLA alleles that are non-essential for antiviral activity, this risk can likely be further reduced.

2 STUDY DESIGN

2.1 Primary Objective

This phase I/II trial is designed to evaluate the feasibility, safety and anti-viral activity of partially HLA-matched VSTs for treatment of persistent EBV, CMV, and/or adenovirus infections in patients who have undergone HSCT, or in patients with PID who have not undergone HSCT.

2.2 Rationale for Study Design

The primary purpose of this pilot study is to assess the safety and feasibility of administering partially HLA-matched VSTs in transplant or PID patients with EBV, CMV, and/or adenovirus infection refractory to standard therapy. Following study enrollment, high-resolution HLA typing of the patient and HSCT donor (if applicable) will be sent to Children's National Hospital along with details regarding the targeted virus(es), and an inquiry of the VST bank will be performed to determine if a partially HLA-matched VST product exists that has antiviral activity against the targeted virus(es) mediated through one or more shared HLA alleles.

We have elected to use a dose of 2×10^7 VSTs/m^2, which has been shown to be safe and have clinical activity in a Phase I study using the same T cell product in both donor-specific and third-party settings. This dose has been utilized in pediatric patients, including infants under 1 year of age, without evidence of increased toxicities in infants in comparison with older children. Of the patients treated under IND 15779 at Children's National to date, 5 were infants under 1 year of age. These infants had no more clinically significant adverse events than older patients (Mean AEs per infusion: 1.3, versus 1.1 in older patients), and median AE grade was the same in infants versus older patients (grade 2). We plan a 30-day monitoring period for immediate toxicities following each infusion. In patients treated with VSTs under IND 15579, all adverse events of grade >=4 (by CTCAE 4.03 classification) occurred in the 30 days following infusion, as did 74% of all AEs reported during the current 45-day safety monitoring period. Accordingly, we are confident that a shorter initial monitoring will not impart higher risk to the subjects.

Because the persistence of adoptively-transferred cells may be shorter using a partially HLA-matched banked VST product, we have included an option of administering additional doses in subjects that have a either a partial response or no response with stable disease and no toxicities after one dose. As many patients in prior studies have required multiple VST doses to

achieve clinical responses, initial analysis of safety and efficacy at 30 days would permit subsequent infusions within a reasonable time frame.

Based on Leen *et al*., antiviral efficacy can be achieved with limited HLA matching between VST and recipient, as long as antiviral activity is mediated through one or more shared HLA alleles.[83] Therefore, we propose to allow infusion of lines that match at 1 or more HLA alleles, with expected antiviral activity against the infecting virus through shared allele(s), although preference will be given to lines matching at the most loci where anti-viral activity through a shared allele has been confirmed.

For CMV infection, standard therapy is well defined as antiviral agents with ganciclovir being the agent of choice and foscarnet or cidofovir being effective second line agents.[11,98] For EBV infection, rituximab is the current treatment of choice for patients with $CD20^+$ lymphoma.[7] For patients with $CD20^-$ tumors there is no clear standard of therapy although most physicians would likely administer chemotherapy. There is also no clear standard therapy for adenovirus, although cidofovir and brincidofovir are often utilized with some evidence of efficacy.[34,36] We therefore have chosen to use cidofovir as standard therapy for adenovirus infection although this requirement would be waived if the subject could not tolerate these agents due to nephrotoxicity.

2.3 Eligibility

2.3.1 Inclusion Criteria

Patients who have received any type of allogeneic transplant or who have a primary immunodeficiency disorder[99,100] will be eligible if they have CMV, adenovirus, and/or EBV infection/disease with failure of treatment (*as defined 2.3.1.2*) after 7 days of standard therapy OR if unable to tolerate standard therapy.

1. Patients must meet one of the following criteria:
   A. Recipient of prior myeloablative or non-myeloablative allogeneic hematopoietic stem cell transplant using either bone marrow or peripheral blood stem cells or single or double cord blood within the previous 18 months, with stable chimerism (defined as >95% donor chimerism [CD33 or whole blood chimerism], OR >90% donor chimerism [CD33 or whole blood] with <5% change between subsequent tests separated by at least 1 week).

   OR

   B. Have a diagnosed primary immunodeficiency disorder (as defined by clinical and laboratory evaluations)[100] and not undergone HSCT.

2. Age ≤ 25 years old.

3. Treatment of the following persistent or relapsed infections despite standard therapy (Table 1).

   <u>a. CMV: Treatment of persistent or relapsed CMV disease or infection after standard therapy.</u> For CMV infection, standard therapy is defined as antiviral therapy with ganciclovir, foscarnet or cidofovir for at least 14 days.

      i. <u>CMV disease:</u> defined as:

1. Demonstration of CMV by biopsy specimen, culture, or molecular testing (PCR, antigenemia) from visceral sites of disease,
2. Detection of CMV by culture or direct fluorescent antibody stain, or PCR in broncheoalveolar lavage fluid in the presence of new or changing pulmonary infiltrates or respiratory disease,
3. Findings consistent with CMV retinitis on ophthalmologic examination.

ii. CMV infection: defined as the presence of CMV positivity as detected by PCR from at least ONE site such as stool or blood or urine or nasopharynx.

Failure of antiviral therapy: defined as a rise or a fall of less than 1 log in viral load in peripheral blood or any site of disease (as measured by PCR compared with baseline levels taken immediately prior to initiation of antiviral therapy) after at least 14 days of antiviral therapy, or inability to tolerate standard antiviral therapy due to toxicities. If antiviral medications have recently been changed, patients must be on the same antiviral medication for at least 7 days prior to enrollment on trial.

b. Adenovirus: Treatment of persistent or relapsed adenovirus infection or disease despite standard therapy. Standard therapy is defined as antiviral therapy with cidofovir or brincidofovir.

i. Adenovirus infection: defined as the presence of adenoviral positivity as detected by PCR or culture from ONE site such as stool or blood or urine or nasopharynx.
ii. Adenovirus disease: defined as the presence of adenoviral positivity as detected by PCR, DFA or culture from two or more sites such as stool or blood or urine or nasopharynx.
iii. Failure of therapy: defined as either an increase, or a fall of less than 1 log in viral load in peripheral blood or any site of disease (as measured by PCR compared with baseline levels taken immediately prior to initiation of antiviral therapy) after at least 14 days of antiviral therapy, or failure of therapy if patient cannot tolerate cidofovir or brincidofovir therapy due to toxicities including poor renal function. If patients have been switched from cidofovir to brincidofovir or vice versa, they must consistently be on the same medication for at least 7 days prior to enrollment on trial.

c. EBV: Treatment of persistent or relapsed EBV infection despite standard therapy. For EBV infection, standard therapy is defined as rituximab given at 375 mg/m^2 in patients for 1-4 doses with a $CD20^+$ tumor. Only B-cell lymphoproliferative disease would be eligible for treatment under this trial, as T/NK severe chronic active EBV (SCAEBV) has not been amenable to third party VSTs in prior studies.

i. EBV infection: defined as
   1. Biopsy proven B-cell lymphoma with EBV genomes detected in tumor cells by immunocytochemistry or in situ PCR
   2. Clinical or imaging findings consistent with EBV B-cell lymphoma and/or elevated EBV viral load in peripheral blood.
ii. Failure of therapy is defined as

1. Increase, or less than 50% decrease in overall disease burden of EBV-PTLD at sites of disease by imaging measurements (in comparison with last imaging prior to rituximab therapy) after at least 14 days following the 1st dose of rituximab.
2. Increase, or a fall of less than 1 log, in EBV viral load in peripheral blood or any site of disease (as measured by PCR compared with baseline levels taken immediately prior to initiation of antiviral therapy) after at least 14 days following the 1st dose of rituximab.

d. Inability to tolerate standard antiviral therapy is defined as:

i. History of allergic reaction to antiviral medication
ii. Pre-existing liver or kidney disease (grade III – IV) that would preclude safe use of antiviral therapy.
iii. Adverse reaction to antivral mediation (i.e. hepatitis, renal insufficiency, cytopenias, other organ disease not attributable to viral infection).
iv. Very young infants (<6 months of age) may proceed if otherwise eligible without failing antiviral therapy as there is no standard in very young infants.

4. Patients with simultaneous infections with CMV, EBV and/or Adenovirus infections are eligible if one or more infection(s) is persistent or relapsed despite standard therapy as defined above. Patients with multiple infections with one or more reactivation and one or more controlled infection are eligible to enroll. For patients with multiple persistent viral infections, the patients would be enrolled onto the primary stratum (CMV or adenovirus). For patients with both CMV and adenovirus infections, the patient would be recruited to whichever strata has fewer subjects.

5. Clinical status at enrollment that allows tapering of steroids to equal or less than 0.5 mg/kg/day prednisone (or equivalent) prior to infusion of the VST doses.

6. Negative pregnancy test in female patients if applicable (childbearing potential who have received a reduced intensity conditioning regimen).

7. Written informed consent and/or signed assent line from patient, parent or guardian.

Table 1: Criteria for defining Viral Persistence

	CMV	EBV (either criteria)		Adenovirus
		**Viral load**	**Imaging**	
Definition of Viral Persistence	Decrease in viral load of <1 log from baseline PCR in blood or other body fluid	Decrease in viral load of <1 log from baseline PCR in blood or other body fluid	<50% decrease in overall disease burden of EBV-PTLD at sites of disease by imaging measurements (in comparison with last imaging prior to rituximab therapy)	Decrease in viral load of <1 log from baseline PCR in blood or other body fluid

Standard Therapies	Ganciclovir, Foscarnet, or Cidofovir	Rituximab	Cidofovir or Brincidofovir
Minimum time on therapy	14 days		

2.3.2 Exclusion Criteria

1. Patients receiving ATG, Campath, Basiliximab, Brentuximab, Tociluzimab or other immunosuppressive monoclonal antibodies impacting T-cells survival within 28 days of screening for enrollment or <5 medication half-lives elapsed (whichever is shorter).

Drug	Half-life	Minimum eligibility time
ATG, equine	6 days	28 days
ATG, rabbit	3 days; longer range of efficacy	28 days
Alemtuzumab	12 days	28 days
Brentuximab	4-6 days	25 days
Basixilimab	7 days	28 days
Infliximab	7-9 days	28 days
Etanercept	102 hrs	20 days
Tocilizumab	11-13 days	28 days

2. Patients who have received donor lymphocyte infusion (DLI) or other experimental cellular therapies within 28 days.

3. Current therapy with ruxolitinib or other JAK inhibitors within the previous 3 days.

4. Patients who have received immune checkpoint inhibitors with <3 medication half-lives elapsed.

Drug	Half-life	Minimum eligibility time
Pembroluzimab	25 days	75 days
Nivolumab	27 days	78 days
Ipilimumab	15 days	45 days
Atezolizumab	21 days	63 days
Avelumab	6 days	18 days
Durvalumab	17 days	51 days
Cemiplimab	19 days	57 days

5. Patients with other uncontrolled infections, defined as bacterial or fungal infections with clinical signs of worsening despite standard therapy. For bacterial infections, patients must be receiving definitive therapy and have no signs of progressing infection for 72 hours prior to enrollment. For fungal infections, patients must be receiving definitive systemic anti-fungal therapy and have no signs of progressing infection for 1 week prior to enrollment.

Progressing infection is defined as hemodynamic instability, worsening physical signs, or radiographic findings attributable to infection. Persisting fever without other signs or symptoms will not be interpreted as progressing infection.

6. Patients with active and uncontrolled relapse of malignancy (if applicable).

7. Patients with severe combined immunodeficiency who are less than 100 days post α/β TCR depleted HSCT.

2.3.3 Informed Consent

The informed consent process will begin at recognition of subject eligibility and consent will be obtained per institutional practices before study therapy is initiated. Consent will be followed by : a) VST bank inquiry and b) treatment.

1. VST bank inquiry: The search for a suitable matched VST line is initiated if the patient is eligible and enrolls in the study. If a suitable partially PHA-matched VST product is not available, the following data will be collected: demographic data, HLA type, infection type and outcome data.
2. Treatment: If a suitable matched VST product is available, the principal investigator or designee discusses the available line with the treating physician. The patient can then receive the identified VST product if eligibility criteria are still met.

2.3.4 Donor Eligibility

2.3.4.1 The VST products that will be used in this third-party study derive from two sources:

A) Products that were previously manufactured for IRB approved protocols Pro00004205 or Pro00003940 at Children's National Hospital. These donors were initially chosen as transplant donors because they were the best match with the original transplant recipient and they met eligibility criteria. There are either additional vials available after the product was used in the donor-specific setting, or was not infused either because the original recipient did not need the VSTs or the original recipient was ineligible. These donors gave consent for products not used for the original recipient to be used in the third party setting.

B) Additional products manufactured for third party use from individuals recruited under this protocol. We will approach healthy donors at Children's National Hospital and Children's Hospital of Los Angeles to ascertain if they are willing to enroll and donate blood for the current study. Some of these individuals may have served as bone marrow donors for unrelated studies, whereas others may be recruited exclusively for VST donation.

For all third party products, donors must meet standard eligibility criteria for donation of blood or marrow. They have been screened with the standard blood bank donor questionnaire, medical history and testing for infectious disease markers by a physician who is experienced in screening transplant donors. All new and previous donors must also meet our supplemental suitability criteria for third-party donors of immune effector therapy (SOP CTL019). These additional criteria are designed to limit eligibility to healthy donors without any prior risk factors for allosensitization or immune-mediated disease. Only donors who have cleared this process and were deemed to be eligible

and suitable will be asked to donate blood for VST generation. For lines that were previously manufactured for Pro0004205, only those where the donor is eligible and suitable may be included in the third party bank.

The results of the physician assessment and ID testing were reviewed by a CETI laboratory director who gave the final eligibility determination according to the SOP for Donor Evaluation.

The processes discussed in the protocol and related manufacturing SOPs are in compliance with 21CFR1271.

2.4 Treatment Plan

2.4.1 VST Line Selection and Availability

2.4.1.1 VST Product Banking and Phenotyping

We will use multivirus-specific VSTs which have been generated for use on our donor-specific studies under INDs 15779 and 15714, as well as VSTs generated from donors recruited under this protocol. All products are generated using a rapid protocol whereby clinical grade pepmixes spanning immunogenic antigens from each of the target viruses are used to directly stimulate PBMCs, followed by expansion in the presence of growth promoting cytokines and the G-Rex culture device optimized for T cell expansion. Donor-specific VST products generated according to this protocol were previously found to be safe and effective in a Phase I clinical trial as discussed.[22]

To initiate the multivirus-specific VSTs, $15x10^6$ PBMCs were pulsed with a master mix of pepmixes spanning Adv (Hexon and Penton), CMV (IE1 and pp65), EBV (LMP2, EBNA1). After 30-60 minutes, the cells/pepmix combination was transferred directly to the G-Rex culture device. The VST media was supplemented with the cytokines IL7 and IL4 (10ng/ml and 400U/ml, respectively) in order to inhibit apoptosis and promote expansion of multivirus-specific T cells.

The VST products were checked for identity, phenotype, sterility, non-alloreactivity, and were cryopreserved prior to administration according to our SOP. To test the functional antigen specificity of the VST we will use individual pepmixes spanning each of the viral antigens used in the initial stimulation as a stimulus in IFNγ ELIspot. Epitope mapping will be performed using known HLA epitope peptides, multimer staining, or peptide mini-pools to identify novel immunogenic epitope peptides within our target antigens.

2.4.1.2 MHC Restriction Mapping and Criteria for Selection of VST products

Following recipient HLA typing, the selection of the ideal VST line will be determined by identifying banked products with established anti-viral activity through one or more shared HLA alleles. In general, preference will be assigned to the infusion of a product with confirmed virus-specific activity against the infecting virus through a shared HLA allele rather than the overall level of HLA match. For example for a patient with an adenovirus infection a line that matches at 2 loci but that has recognition of adenovirus mediated through those antigens would be preferable to a line matched at 3 loci but with no demonstrated activity against adenovirus.

The strategy for classifying antiviral MHC restriction of VSTs will be as follows:

A. IFN-γ Elispot: VSTs are tested using antigen pepmixes encompassing entire viral antigens (CMV pp65, IE1, EBV EBNA1, LMP2, Adv Hexon, Penton), as well as individual 9-15 amino acid peptides that have previously described MHC restrictions. These individual peptides will be chosen based on donor HLA typing. ELISPot results of ≥30 spots/well (utilizing 1x105 cells/well) will be considered specific for the tested peptide.

B. For donor HLA alleles for which viral multimers are available (HLA-A01, A02, A03, A24, B07, B11, B27, and B35), MHC multimer staining will be performed to determine the proportion of epitope-specific VSTs.

C. For VSTs with still unknown antiviral MHC restrictions, peptide array testing will be performed using IFN-γ ELISpot. Arrays are performed by testing a full library of 15-mer peptides encompassing the targeted viral antigen. All peptides eliciting a response of >30 spots/well will be further analyzed using online HLA prediction algorithms (SYFPEITHI, Immune epitope database). Confirmation of HLA restriction can also be further confirmed by utilizing ^{51}Cr release cytotoxicity testing, by using partially HLA-matched lymphoblastoid cells or PHA blasts that have been pulsed with the peptide of interest, and determining if the VST product is capable of specific lysis of the partially-HLA matched targets.

The initial VST matching algorithm will be as follows:

A. VST will be identified from the bank which have antiviral activity against the targeted virus(es) mediated through one or more HLA alleles that is shared with the recipient and HSCT donor (if applicable).

B. If multiple products exist that meet criteria A, the following rules will apply:

B.1. The product that is best matched with the recipient and HSCT donor at HLA-A/B/C/DRB1/DQB1 will be chosen.

B.2. For subjects with PID who have not undergone HSCT but have a donor identified for future HSCT, and there is any HLA mismatch between the recipient and future donor, the study team will aim to avoid products expressing the same mismatched donor HLA alleles (in order to minimize the chance of allosensitization). At all other alleles, the product that is best matched at HLA A/B/C/DRB1/DQB1 will be chosen.

This algorithm will evolve as more is learned about the hierarchy of immunodominant viral epitopes over the course of this study.

2.4.1.3 No Matched VST Product Available

If no matched VST product is available, the patient will be registered so that the feasibility of the approach can be assessed and the eventual outcome will also be collected.

2.4.1.4 VST Product Available but Patient Status Changes

Patients with a clinical course that changes between screening and infusion and renders patient ineligible at time of infusion will not be given the VST and will be followed for eventual outcome.

2.4.2 Administration and Monitoring

2.4.2.1 Partially HLA-matched VSTs will be thawed and given by intravenous injection over 1-2 minutes. Patients will receive 2×10^7 partially HLA-matched VSTs/m^2 as a single infusion.

2.4.2.2 Premedications: Patients without prior history of reaction to blood products generally do not require premedication. If patients receive premedication, Benadryl 0.25-0.5 mg/kg (max 25 mg) PO/IV and/or Tylenol 5-10 mg/kg (max 650 mg) PO may be given.

2.4.2.3 Patients will be monitored according to institutional standards for administration of blood products and at a minimum will be monitored according to below:

- If given to an outpatient, patients should remain in the clinic for at least one hour.
- Patients should remain on continuous pulse oximetry for at least 30 minutes.
- Vital signs should be monitored at the end of infusion then at 30 and 60 minutes.

2.4.2.4 Supportive Care: Patients will receive supportive care for acute or chronic toxicity, including blood components or antibiotics, and other intervention as appropriate.

2.4.2.5 If a patient has a partial response (as defined by a 1-log decrease in viral load without viral clearance), stable disease (as defined by changes in viral load insufficient to qualify as partial response, but with a less than 1 log increase in viral load from the time of initial infusion, with no evidence of dissemination to other sites of disease), or receives medication (such as steroids), which may affect the persistence or function of the infused VST, and has no dose-limiting toxicities from the intitial dose(s) of VSTs, they are eligible to receive up to 3 additional doses at the same initial dose at day 30 post-infusion and at minimum 2 week intervals thereafter. If the additional doses are post steroid administration, the steroid dose must be decreased to a dose of 0.5 mg/kg/day of Prednisone (or equivalent) or lower before the patient may receive additional doses. Additional doses may be from the same donor or a different donor. Decision to switch to a different donor can be made by the principal investigator based on factors that include sequential treatment of different viral infections, concerns for immune escape of the targeted virus, and/or availability of a better matched or otherwise superior VST product.

2.4.2.6 Regular screenings for cytokine release syndrome (CRS) using baseline and weekly inflammatory markers and cytokine analysis is recommended, particularly for patients in the Intensive Care Unit and those with high viral loads (log 5 or higher). Standard recommendations for CRS workup and treatment are enclosed in Appendix II.

2.4.3 Antiviral medication guidelines for study subjects

2.4.3.1 Following VST administration, antiviral medication use is encouraged to remain steady if clinically feasible. Guidelines are suggested for study subjects in **Tables 2-3**.

2.4.3.1 Use of antivirals following VST therapy will ultimately depend on the clinical judgement of the subject's transplant physician with guidance from the investigators.

Table 2: Suggested Antiviral guidelines for Study Patients

Viral load / clinical symptoms	Antiviral status	
	**For patients unable to continue antiviral medications due to prior toxicities**	**For patients who are able to continue antiviral medications at the time of VST infusion**
Undetectable or <1000 copies/ml	No change	Consider discontinuation of antiviral therapy.
< 1 log decrease	No change	Continue antiviral therapy
< 1 log decrease or < 1 log increase with unchanged clinical symptoms	No change	Continue antiviral therapy
≥ 1 log increase with no change in clinical symptoms	If clinically well, consider holding off on antiviral therapy. If unstable, begin antiviral therapy	Continue antiviral therapy
≥ 1 log increase with new clinical symptoms attributable to viral progression	Restart antiviral therapy if feasible based on organ function.	Continue antivirals. Consider change in antivirals if genotypic evidence or clinical suspicion of resistance

Table 3: Guidelines for Suggested Antiviral Agents for Study Patients

Virus	Resistance	Organ function	Recommended agent
CMV	None	Optimal	Ganciclovir
	None	Marrow toxicity / cytopenias	Foscarnet
	UL97	Optimal	Foscarnet
	UL54	Optimal	Cidofovir
EBV	N/A	N/A	Rituximab
Adenovirus	N/A	Optimal	Cidofovir, 5mg/kg/week
	N/A	Renal dysfunction	Brincidofovir

2.5 Risks and Toxicities

2.5.1 Graft Versus Host Disease (GVHD)

The risk that adoptively transferred partially HLA-matched VSTs will cause Grade II or higher GVHD is low according to data from previous studies with 3rd party VSTs specific for adenovirus, EBV and CMV (Section 1.10). If any subject develops GVHD they will receive standard GVHD treatment according to established criteria.[101]

2.5.2 Acute GVHD Scoring

Acute GVHD will be monitored for 30 days following infusion. Weekly GVHD organ stage scores, overall clinical grade, biopsy information for GVHD and relevant differential diagnosis will be recorded. The weekly score will encompass all information since the last assessment. Organ involvement, biopsy information, staging, differential diagnosis, and GVHD therapy will be documented in the medical record using the Blood and Marrow Transplant Clinical Trials Network (BMT CTN) GVHD scoring stamp or equivalent. A sample assessment for acute GVHD is shown below.

Clinical Acute GVHD Assessment

Today's date _________ Patient ID ____________________ Karnosfky/Lansky _________

	Codes 0	1	2	3	4		Differential diagnosis: GVHD	Drug Rxn	Cond Reg	Infect	TPN	VOD	Other
Skin	☐	☐	☐	☐	☐	% body rash: _____	☐	☐	☐	☐			_________
Liver	☐	☐	☐	☐	☐	Current bili: ______	☐	☐	☐	☐	☐	☐	_________
Lower GI	☐	☐	☐	☐	☐	Vol: ____________	☐	☐	☐	☐	☐		_________
Upper GI	☐	☐					☐	☐	☐	☐	☐		_________

Systemic agents: ☐ CSA ☐ Tacrolimus ☐ Infliximab ☐ Dacluzimab
☐ Pentostatin ☐ Sirolimus ☐ Etanercept ☐ MMF
☐ Prednisone ☐ Methylprednisolone ☐ Ontak ☐ Other _________

Current steroid dose: Prednisone ______________mg/kg/day; Methylprednisolone ____________ mg/kg/day

Has the steroid dose been increased to ≥ 2.5 mg/kg/day of prednisone (or 2 mg/kg/day methylpred)? ☐YES ☐ NO
If yes, date dose increased _________; reason for dose increase: ☐GVHD flare ☐ Other (specify) __________

Topical agents: Skin topical steroids**:** ☐ YES ☐ NO; Non-absorbed oral steroids (e.g. Budenoside, Entocort) ☐ YES ☐ NO

Chronic GVHD: Does the patient have evidence of chronic GVHD? ☐ YES ☐ NO

**Codes**	**Skin**	**Liver (bilirubin)**	**Lower GI (stool output/day)**	**Upper GI**
**0**	No rash	≤ 2 mg/dl	Adult: < 500 ml/day<br>Child: < 10 ml/kg/day	No protracted nausea and vomiting
**1**	Maculopapular rash<br>< 25% BSA	2.1-3 mg/dl	Adult: 500–1000 ml/day<br>Child: 10 -19.9 ml/kg/day	Persistent severe nausea, vomiting with a positive upper GI biopsy
**2**	Maculopapular rash<br>25 – 50% BSA	3.1-6 mg/dl	Adult: 1001-1500 ml/day<br>Child: 20 – 30 ml/kg/day	
**3**	Generalized erythroderma (>50% BSA)	6.1-15 mg/dl	Adult: >1500 ml/day<br>Child: > 30 ml/kg/day	
**4**	Generalized erythroderma (>50% BSA) plus bullous formation and desquamation > 5% BSA	>15 mg/dl	Severe abdominal pain with or without ileus, or grossly bloody stool or melena (regardless of stool volume)	

Signature ___________________________________ Date _______________

Grading Index of Acute GVHD

	Skin	Liver	Gut	Upper GI
0	None and	None and	None and	None
I	Stage 1-2 and	None and	None	None
II	Stage 3 and/or	Stage 1 and/or	Stage 1 and/or	Stage 1
III	None-Stage 3 with	Stage 2-3 or	Stage 2-4	N/A
IV	Stage 4 or	Stage 4	N/A	N/A

2.5.3 Chronic GVHD

Patients developing sign/symptoms of chronic GVHD (CGVHD) will have symptoms recorded on the CGVHD case report form at the scheduled follow-up visits. Please reference Appendix I for the Chronic GVHD Definitions and Scoring Table.

Table 4: Definite and Possible Manifestations of Chronic GVHD

Organ System	Definite manifestations of chronic GVHD	Possible manifestations of chronic GVHD
Skin	Scleroderma (superficial or fasciitis), lichen planus, vitiligo, scarring alopecia, hyperkeratosis pilaris, contractures from skin immobility, nail bed dysplasia	Eczematoid rash, dry skin, maculopapular rash, hyperpigmentation, hair loss
Mucous membranes	Lichen planus, non-infectious ulcers, corneal erosions/non-infectious conjunctivitis	Xerostomia, keratoconjunctivitis sicca
GI tract	Esophageal strictures, steatorrhea	Anorexia, malabsorption, weight loss, diarrhea, abdominal pain
Liver	None	Elevation of alkaline phosphatase, transaminitis, cholangitis, hyperbilirubinemia
GU	Vaginal stricture, lichen planus	Non-infectious vaginitis, vaginal atrophy
Musculoskeletal/ Serosa	Non-septic arthritis, myositis, myasthenia, polyserositis, contractures from joint immobilization	Arthralgia
Hematologic	None	Thrombocytopenia, eosinophilia, autoimmune cytopenias
Lung	Bronchiolitis obliterans	Bronchiolitis obliterans with organizing pneumonia, interstitial pneumonitis

2.5.4 Systemic Inflammatory Response Syndrome (SIRS)

SIRS is a rare systemic inflammatory response associated with fever, tachycardia and hypotension that has been seen in 2 of 176 patients who received virus-specific CTLs post hematopoietic transplant. Both patients had extensive bulky disease and developed the complication during a therapeutic response. This syndrome has also been reported in patients receiving T cells modified with chimeric antigen receptors during therapeutic responses. In

several previously reported cases, elevated cytokine levels were found in the blood. In the reported patients, the symptoms resolved after treatment with steroids and/or anti-cytokine antibodies including anti-TNF and anti-IL-6. In patients who develop symptoms consistent with SIRS in the absence of other causes, a standardized workup should be followed (example provided in Appendix II: CNMC BMT SOP 014: AE Management F CTL Infusions).

2.5.5 Allosensitization and Resistance to Stem Cell Engraftment

Recipient production of donor-specific anti-HLA antibodies is a known risk factor for allograft rejection in solid organ transplantation and HSCT, and would be a theoretical risk in patients with PID who receive VST therapy prior to HSCT. However, this has not been described in patients with PID. Given: 1) the moderate to severe T and B-cell dysfunction in many forms of PID, and 2) the requirement for pre-HSCT conditioning (chemotherapy) to ensure multilineage engraftment, which should eliminate any alloreactive cells, this risk is deemed to be very low. Regardless, we plan to further minimize the chances of allosensitization to a subsequent stem cell product by utilizing a selection strategy for comparing third party donors to HSCT donors (if known), as detailed in Section 2.4.1.2.

In previous studies of third-party VST therapy, there have not been any noted increase in primary or secondary engraftment failure. However, as the VSTs are partially HLA-matched, this is a theoretical risk. Given the potential risk, patients with decreasing myeloid donor chimerism after HSCT will be ineligible for VST infusion.

Patients with SCID who undergo T-cell depleted HSCT are also at risk for engraftment failure, and given the profound lymphopenia during the first 100 days following T-cell depleted HSCT, this period may theoretically favor expansion of highly rare populations of alloreactive T-cells with subsequent impact on an allograft. For this reason, patients with SCID who have undergone a/b T-cell depleted HSCT will not be eligible for VST infusion until on or after day +100.

The details of subsequent HSCT for each patient will be collected, including degree of HSC donor/recipient HLA matching, the administered HSC dose, and the time to myeloid and lymphocyte engraftment.

2.5.6 Other Toxicities

VST Infusion may also cause toxicities including gastrointestinal bleeding, thrombotic microangiopathy (TMA), pulmonary hemorrhage, and anaphylaxis. Should unanticipated toxicities arise (e.g. severe local reactions or hepatorenal damage) they, too, will be graded by the NCI Common Terminology Criteria for Adverse Events (CTCAE), Version 4.03.

2.5.7 Management of Toxicity

VSTs are susceptible to killing by steroids given at a dose of 1-2 mg/kg. This is standard therapy for GVHD and could also be given if a recipient develops other complications considered possibly related to VST administration. Anti TNF-alpha receptor antibody and anti IL-6 also may have some efficacy in individuals that develop SIRS after T cell infusion and may be considered in patients with consistent clinical signs/symptoms.[102] Other supportive care would be per standard medical practice.

3 STUDY ENDPOINTS

3.1 Primary Endpoints

The primary objectives for this Phase I/II study is to evaluate the feasibility, safety, and antiviral efficacy of partially HLA-matched VSTs for treatment of persistent EBV, CMV, and/or adenovirus infections in patients who have either undergone HSCT, or in patients with PID prior to HSCT.

3.1.1 Feasibility

Feasibility will be defined as the ability of the investigators to identify suitable partially HLA-matched VST products from the VST bank at Children's National Hospital for referred study subjects. The percentage of referred patients with potential partially-matched VST products identified will be recorded, as will timing between patient referral and treatment.

3.1.2 Safety

Safety of administration of VSTs will be evaluated for 30 days for GVHD and for other toxicities. The safety endpoint, dose-limiting toxicity (**DLT**), will be defined as acute GvHD grades III-IV or grades 3-5 infusion-related adverse events or grades 4-5 non-hematological adverse events related to the T cell product within 30 days of each VST dose and that are not due to the pre-existing infection or the original malignancy or pre-existing co-morbidities as defined by the NCI Common Terminology Criteria for Adverse Events (CTCAE), Version 4.03. Toxicities to consider include but are not limited to GI toxicity, renal toxicity, hemorrhagic toxicity, cardiovascular toxicity (hypotension, cardiac arrhythmia and left ventricular systolic dysfunction), neurological toxicity (somnolence and seizure), coagulation toxicity, vascular toxicity and pulmonary toxicity. Patients who have not experienced DLT and who withdraw from the study prior to 30 days for reasons unequivocally unrelated to treatment toxicity are not evaluable for the DLT endpoint and will be replaced. Safety endpoints will be monitored by the Data Safety Monitoring Committee (DSMC).

3.1.3 Antiviral Efficacy

Peripheral blood and, where relevant, stool and urine will be monitored for CMV, EBV, and/or adenovirus viral load. For patients with multiple viral infections, the response against the primary viral target will determine the classification. For the infection under treatment response in viral load will be assessed at 30 days after the first VST infusion, and will be defined as follows (Table 5):

Complete response: Return to normal range as defined by specific assay used for the targeted viral infection(s) in blood.
Partial response: Decrease in viral load of at least 1-log from baseline. For EBV-PTLD ≥50% decrease in radiographic disease burden from baseline would also be considered a partial response.
Stable disease: Changes insufficient to qualify as partial response, but with <1 log increase in viral load with no evidence of dissemination to other sites of disease.
Progression: Increase in viral load of at least 1 log from baseline or dissemination to other sites of disease for at least one targeted infection.

Patients are evaluable for response if they receive at least one VST infusion and are followed until at least one post-baseline assessment of viral load. Patient who are not evaluable for response will be replaced. Viral response data will be reviewed by an independent committee of 3 members to ensure agreement and unbiased assignment of clinical response classifications.

3.2 Secondary Endpoints

3.2.1 Reconstitution of Antiviral Immunity

Patient serum and peripheral blood mononuclear cells will be monitored for virus-specific activity during the 3 months following VST infusion by phenotypic and functional studies including ELIspot with appropriate viral specific peptide mixtures and available HLA-restricted epitope peptides, intracellular cytokine staining, serum cytokine profiling and/or other assays as they become available for immune profiling purposes.

3.2.2 Persistence of infused VSTs

Persistence of infused T cells will be monitored at 1 month and 3 months following VST infusion using deep sequencing and additional tests as indicated to track the TCR v-beta repertoire in the patient peripheral blood prior to and post-infusion.

Table 5: Definitions of Antiviral Responses

	**CMV**	**EBV (either criteria)**		**Adenovirus**
		Viral reactivation / viremia	***Lymphoproliferative disease***	
**Complete response**	Resolution of viremia or viral detection in body fluid analyzed at baseline	Resolution of viremia or viral detection in body fluid analyzed at baseline	Lymphoproliferative disease: Resolution of radiographic disease	Resolution of viremia or viral detection in body fluid analyzed at baseline
**Partial response**	≥1 log reduction in viral load from baseline	≥1 log reduction in viral load from baseline	≥50% decrease in radiologic disease burden from baseline	≥1 log reduction in viral load from baseline
**Stable disease**	< 1 log reduction, but <1 log increase in viral load	< 1 log reduction, but <1 log increase in viral load	<50% improvement but <50% increase in radiologic disease	< 1 log reduction, but <1 log increase in viral load
**Progression**	≥ 1 log increase in viral load or dissemination to new sites of disease	≥ 1 log increase in viral load or dissemination to new sites of disease	≥50% increase in radiologic disease or dissemination to new disease sites	≥ 1 log increase in viral load or dissemination to new sites of disease

3.2.3 Effects on Clinical Signs of Viral Infection

If a patient has organ involvement, clinical response will be monitored. For patients with EBV lymphoma and measurable disease, response will be assessed by RECIST criteria.

3.2.4 Survival

Overall survival at 6 and 12 months post VST infusion will be computed.

3.2.5 Chronic GVHD

Chronic GVHD will be assessed 6 and 12 months post VST infusion.

3.2.6 Viral Reactivations

All CMV, EBV and adenovirus infections/reactivations occurring within 12 months of VST infusion will be collected. Information about infection or reactivation with other viruses may be collected as well.

3.2.7 Effect on Primary HSC Engraftment and Secondary Graft failure

Timing to primary engraftment of subsequent cell products will be collected, as defined as times to myeloid engraftment and lymphoid engraftment (defined by ANC>500/mm^3 and ALC>500/mm^3 for three consecutive measurements on different days, respectively). Primary engraftment failure would be defined as failure to achieve myeloid engraftment within 3 months of HSCT, requiring re-transplantation.

Secondary graft failure is defined as initial neutrophil engraftment followed by subsequent decline in the ANC to <500/mm^3 for three consecutive measurements on different days, unresponsive to growth factor therapy that persists for at least 14 days in the absence of a known cause such as relapse. Secondary graft failure will be assessed at 30 days post VST infusion.

4 PATIENT ENROLLMENT AND EVALUATION

4.1 Enrollment

The informed consent process will begin at recognition of subject eligibility and consent will be obtained per institutional practices before study therapy is initiated. This protocol will be discussed with eligible patients and, when appropriate, their guardians. The informed consent process will be conducted in a private room to respect subject privacy. Following the briefing of the research study, the coordinator or designee will provide the subject with ample time to read the consent, and will answer any of their questions regarding the document. No study procedures will occur before the subject gives informed consent.

Consent will be followed by a) screening for a VST product, and b) treatment.

1. The screening portion consists of registering demographic data and patient eligibility data. The search for a suitable matched virus VST line is initiated if the patient is eligible. If a VST line is not available, clinical data will still be collected, including demographic data, HLA type, infection type and follow up data regarding viral clearance and survival.
2. If a suitable matched VST line is available and the patient is deemed eligible, the principal investigator or designee discusses the available product with the treating physician. The patient can then receive the identified VST line if eligibility criteria are still met.
3. The selected product will be shipped to the study site from Children's National Hospital in a Liquid Nitrogen Dry Shipper via overnight shipping. The study site will then thaw the cryovial containing the product, transfer to a sterile syringe, and administer to the patient. The cryovial will then be cultured locally (aerobic, anaerobic, and fungal cultures, held for 14 days) to ensure product sterility at the time of administration. Specific instructions will be provided to all study sites regarding the procedure for thawing and administration of the product.
4. Patients will be assessed for the need for subsequent infusions depending on their antiviral response and presence or absence of toxicities by day 30. If the patient is deemed eligible for further infusions, the selected product will be shipped to the study site for infusion as noted above.

Prior to the subject's participation in any trial procedures, the investigator or designee will ensure that the potential participant understands the research study and their role in the study. The written informed consent will be signed and dated by the subject and by the person who conducted the informed consent discussion. A signed copy of the consent form will be placed in the subject's trial chart and a photocopy will be given to the subject. Informed consent will be obtained in accordance with the Code of Federal Regulations (CFR) 21 CFR 50.25, 32 CFR 219, 45 CFR 46 and 15-2, ICH Harmonized Tripartite Guidance for Good Clinical Practice, and the Belmont Report. Also included in the consent process, Health Insurance Portability Accountability Act (HIPAA) authorization will be obtained before any study procedure is undertaken.

If a subject decides to take part in the research study, their protected health information will not be given out except as allowed by law. Study personnel will work hard to keep this information private. The results of the data from the study may be published. However, the subject will not be identified by name. The subject may change their mind and withdraw from the study any time they want.

All cell culture manipulations will be carried out in the CETI GMP facility using current standard operating procedures (SOPs). After Quality Assurance testing is complete a Certificate of Analysis will be issued.

4.2 Study Monitoring

4.2.1 Follow-up Schedule

The Follow-up Schedule for scheduled study visits is outlined in Table 4.2.2

Follow-up Assessments: The timing of follow-up visits is based on the date of VST infusion. If a patient has multiple VST doses the schedule resets again at the beginning so follow up relates to the last VST dose. Additional follow up assessments may be done based on clinical and laboratory responses.

4.2.2 Assessments

All assessments are considered standard-of-care for patients infected with CMV, EBV, or Adenovirus unless identified below by "*".

Pre-Study

1. History and physical exam including height and weight
2. Viral loads by PCR for EBV, adenovirus and CMV
3. Complete acute GVHD staging and grading information including assessments of rash, diarrhea, nausea/vomiting, weight and liver function tests
4. CBC with differential, platelet count
5. Basic chemistry panel (sodium, potassium, chloride, CO2, BUN, Creatinine, and Glucose)
6. Liver function tests (bilirubin, alkaline phosphatase, AST, and ALT)
7. Urine pregnancy test if female patient of childbearing potential and has received a reduced intensity transplant regimen
8. Samples for research laboratory studies

Pre-Infusion

The following tests to confirm patient eligibility should be done within 24 hours of the VST infusion:

1. CBC with differential, platelet count
2. Basic chemistry panel (sodium, potassium, chloride, CO2, BUN, Creatinine, and Glucose)
3. Liver function tests (bilirubin, alkaline phosphatase, AST, and ALT)

Post-Infusion

1. **THIRD PARTY MULTIVIRUS-SPECIFIC T-CELL INFUSION FORM** must be completed after the infusion.
2. Viral load for the treated virus or viruses will be collected weekly at 1, 2, 3, 4 and 6 weeks, and 3 months post-infusion.Thereafter, viral load results will be followed until 12 months post infusion as clinically indicated. Additional time points may be analyzed as clinically indicated. If the treatment team is also measuring viral loads for other viruses we will follow these results.
3. Complete acute GVHD staging and grading information including assessments of rash, diarrhea, nausea/vomiting, weight and liver function tests weekly until Day 30.
4. Chronic GVHD evaluation (if present) at 3, 6, and 12 months.
5. Liver function tests (bilirubin, alkaline phosphatase, AST, ALT) will be collected weekly at 1, 2, 3, 4, 5 and 6 weeks and at 3 months.
6. CBC with differential and platelet count will be collected weekly at 1, 2, 3, 4, 5 and 6 weeks.
7. Infusion-related toxicities within 24 hours and toxicity evaluation weekly until Day 30, and acute GVHD until Day 30.
8. Steroid dose weekly until Day 30, and at 3 and 6 months.
9. Samples for research laboratory studies at 1, 2, 3, 4, and, 6 weeks and 3 months: 5-20 mls (or a maximum of 2ml/kg) will be collected at each time point in sodium heparin (green top) tubes, and sent to Children's National Hospital for immune reconstitution studies. Shipping will be via overnight priority (Monday-Thursday), and should be sent at ambient temperature. Samples must be labeled with at least two identifiers, as well as the time and date of collection.

   The receiving address is:

   Bollard Laboratory

   Children's National Hospital

   111 Michigan Ave NW, Fifth Floor, Room 5220

   Washington, DC 20010

After the initial follow up for safety is complete at 30 days for toxicity and for GVHD, patients will continue to be followed per routine clinical care post transplant. We will perform laboratory follow-up studies for 3 months post-infusion or longer depending on clinical and laboratory viral responses and will assess these patients clinically at 3, 6 and 12 months to evaluate for any long term effects attributable to the VST infusion.

Research Sample Processing: Research laboratory processing at Children's National will involve separation of plasma via centrifugation and storage at -80°C, and separation of peripheral blood mononuclear cells (PBMC) via Ficoll/Hypaque centrifugation. Fresh PBMC will be utilized as possible for assays as listed below, and the remainder will be frozen in DMSO-containing freeze medium and stored in our liquid nitrogen storage facility for later analysis. A

small fraction of PBMC (between 1-5x10^5) will be placed in 1ml RNAzol and frozen at -80^oC for later TCRβ transcript analysis.

Immune Reconstitution Assays: Assessment of virus-specific immunity will be measured using multiple assays, which could include IFN-γ ELIspot using PBMC stimulated with viral pepmixes from the targeted virus(es), PBMC cytokine profiling via Luminex multiplex assay, intracellular cytokine staining of pepmix-stimulated PBMC, IFN-γ capture flow cytometry of pepmix stimulated PBMC using the MACSQuant platform, multimer staining of PBMC using relevant viral epitope multimers, RNA transcript analysis of sorted T-cell samples via RT-PCR, TCRβ sequencing, and/or other assays as they become available.

Table 6: REQUIRED ASSESSMENTS

	Pre-Study	Pre-Infusion (Day 0)	Day 7	Day 14	Day 21	Day 28	Day 35	Day 45	Day 90	Day 180	Day 365
			±3 days	±3 days	±3 days	±3 days	±3 days	±3 days	±14 days	±28 days	±28 days
History and physical exam	X										
CMV, EBV, and/or adenovirus virus load (if previously positive)	X		X	X	X	X		X	X		
Acute GVHD evaluation	X		X	X	X	X					
Chronic GVHD evaluation									X	X	X
Liver function tests (alkaline phosphatase, bilirubin, AST, ALT)	X	X	X	X	X	X	X	X	X		
Urine pregnancy test[1]	X										
Infusion related toxicity evaluation		X[2]	X	X	X	X					
Steroid dose	X	X	X	X	X	X	X	X	X	X	
Blood and serum for ancillary laboratory studies^	X[3]		X[3]	X[3]	X[3]	X[3]		X[3]	X[3]		
Occurence of bacterial, viral, or fungal infections (aside from targeted viruses)	X		X	X	X	X		X	X		
CBC with Differential and Platelet Count	X	X	X	X	X	X	X	X			
Basic Chemistry	X	X									
Multivirus-specific T-Cell Infusion		X[4]									

[1] Pregnancy test if of child bearing potential and has received a reduced intensity transplant
[2] This evaluation should be done within 24 hours of infusion and may be done by phone if patient is outpatient.
[3] Research procedures beyond that required for usual care of a patient infected with CMV, EBV, or Adenovirus - 5-20mls (or max of 2 mls/kg) will be collected at each time point. Depending on clinical and laboratory responses samples may be collected at additional time points before or after 90 days.
[4] **THIRD PARTY MULTIVIRUS-SPECIFIC T-CELL INFUSION FORM** must be completed

4.3 Off Treatment & Off Study Criteria

4.3.1 Off Treatment Criteria

4.3.1.1 Any patient who develops irreversible, life threatening, Grade 3-4 acute GvHD or grades 3-5 infusion-related adverse events or grades 4-5 non-hematological adverse events 5 days from last VST dose and considered to be primarily related to VST injection will be taken off treatment. In such patients, the toxicities will be followed until resolution and the patient will not be eligible to receive subsequent treatment with VSTs. However, response data will continue to be collected as applicable.

4.3.1.2 Any patient who receives any other hematopoietic cell product will be taken off of treatment, adverse event data collection will cease and the patient will not be eligible to receive subsequent treatment with VSTs. However, response data will continue to be collected as applicable.

4.3.1.3 Any patient who receives therapy for relapse of their primary malignancy will be taken off of treatment, adverse event data collection will cease and the patient will not be eligible to receive subsequent treatment with VSTs. However, response data will continue to be collected as applicable.

4.3.2 Off Study Criteria

4.3.2.1 Completion of study specified procedures.
4.3.2.2 Refusal of further study follow-up by patient or legal guardian
4.3.2.3 Lost to follow up
4.3.2.4 Death

5 STATISTICAL CONSIDERATIONS

5.1 Study Design Synopsis

This study is a phase I/II pilot study to evaluate the feasibility, safety and efficacy of most closely HLA matched multivirus specific VST lines (CHM-VSTs) in HSCT patients and PID patients with infections with CMV, adenovirus, and/or EBV that is persistent despite standard therapy.

Patients will be enrolled into one of three strata:

- Stratum 1 (Primary): Patients with CMV infection
- Stratum 2 (Primary) : Patients with adenovirus infection
- Stratum 3 (Descriptive): Patients with EBV infection

Treatment efficacy (response) will be assessed independently within each stratum. Safety assessment will combine patients across all strata. Target accrual to each of the primary strata 1 and 2 is a minimum of 20 and maximum of 30 response-evaluable patients. Target accrual to descriptive stratum 3 is 10 response evaluable patients. The study will remain open to accrual until it is certain that a minimum of 20 response-evaluable patients have been enrolled in each of the primary strata, unless accrual is halted due to safety concerns (see below). Enrollment to a stratum will be halted once the maximum target accrual of response-evaluable patients has been reached.

Patients with concurrent CMV and adenovirus infections will be enrolled on the primary stratum with the smaller number of patients at that time. Patients with CMV or Adenovirus infection concurrent with EBV infection will be enrolled on the appropriate primary stratum. The primary analysis within each stratum will include only patients assigned to that stratum, although secondary analysis may include patients assigned to multiple strata.

The total sample size of the study will be a minimum of 40 and maximum of 60 response-evaluable patients. Additional patients may be required to replace patients who are not evaluable for response or DLT assessment. A larger number of potential subjects will be screened to assess the feasibility of the approach.

We expect to enroll 15-20 patients per year and therefore expect the study to conclude within the estimated accrual period of 36-48 months.

5.2 Analysis of treatment feasibility

The analysis of treatment feasibility will be descriptive. The percentage of referred patients with potential partially-matched VST products identified will be recorded, as will timing between patient referral and treatment.

5.3 Analysis treatment efficacy

Efficacy, as measured by the proportion of patients achieving at least CR or PR (per section 3.1.3), will be assess independently in each of the three strata. There will be no interim monitoring for futility. As described above, the two primary strata will enroll a minimum of 20 and maximum of 30 evaluable patients. Under the null hypothesis that VST is not active, it is reasonable to assume that that the true spontaneous response rate will be no more than 20%. Thus in each of the two primary strata, based on an exact one-sided one-sample test of proportions of the null hypothesis that the response rate is $\leq 20\%$, with Type I error no greater than 5% and with a sample size of 20, there will be at least 90% power to detect an improvement in response rate to 52%. With a sample size of 30 the detectable improvement is to 47%. Hence this study has sufficient power to detect plausible and clinical important improvements in response rate due to VST in each of the primary strata. There will be no adjustment for multiple comparisons[103]

Analysis of the exploratory stratum will be descriptive, consisting of a simple estimate of the proportion of patients who respond. In primary analysis patients with multiple infections will be included only in the stratum to which they are assigned, but may be included in other strata for secondary analysis.

5.4 Monitoring of patients safety

Dose-limiting toxicities (DLT, section 3.1.2) will be monitored continually.

To ensure adequate safety monitoring in the initial subjects, no more than 5 subjects will be recruited to this trial before the first 3 patients complete the 30 day safety monitoring period.

The proportion of DLT events across all patients enrolled in any stratum will be monitored using a stopping guideline based on a Bayesian rule with binomial likelihood and Beta(0.851,5.63) prior. The risk of DLT of 25% is considered to be moderate and acceptable in this population.

The boundary is defined as a posterior probability of 95% that the true event rate exceeds 25%. Beta parameters were selected to achieve the desired Type I error and minimum average sample size, resulting in the following sequential boundaries that will be used to DLTs:

# Pts	1-4	5	6-8	9-11	12-14	15-17	18-20	21-23
# DLT	--	5	6	7	8	9	10	11
# Pts	24-26	27-29	30-32	33-36	37-39	40-42	43-46	47-49
# DLT	12	13	14	15	16	17	18	19
# Pts	50-52	53-56	57-59	60				
# DLT	20	21	22	23				

If at any time the cumulative number of patients with DLT is equal to or exceeds the boundary listed in the above table, the study will be suspended to accrual pending review of toxicities by the study committee, in consultation with the DSMC and FDA, to determine whether the study should continue as planned, be amended, or be terminated. This stopping rule yields the probability of crossing the boundary at most 6% when the true DLT rate is 25%, with on average 58 patients treated. The probability of crossing the boundary is > 90% when the true DLT rate is 44%, with on average 28 patients treated. Patients who are not evaluable for DLT per section 3.1.2 will be replaced unless it is certain that accrual of remaining patients will not result in crossing of the boundary.

If a patient experiences Grade 5 toxicity that is at least possibly related to the product, we will hold all infusions on the study until both the incident in question and the infused product have undergone a thorough internal review and the outcome discussed with the FDA.

For patients with PID who have not undergone HSCT, a stopping rule would also be set for elevated occurrence of primary graft failure for patients who undergo HSCT following VST infusion. Though this is known to occur in a minority of patients with PID who undergo hematopoietic stem cell transplant, the rate is generally below 20%.[6] Accordingly, if we detect a two-fold or higher incidence of primary engraftment failure (>/= 40%) at any time, recruitment of PID patients who have not undergone HSCT would end. In any case where primary engraftment failure occurs in a patient with PID, the patient in question will be tested for donor-specific alloantibodies.

5.5 Data Analysis

Safety and toxicity outcomes including adverse events, GvHD, clinical signs of viral infections, secondary graft failure and laboratory measurements will be summarized using descriptive statistics (frequency table, means, standard deviations, medians and ranges). Toxicity information including the type, severity, time of onset, time of resolution, and the probable association with the study regimen will be tabulated and summarized.

Response rate including complete response, partial response will be summarized as frequency table. The response rate with 95% CI will be reported. Reconstitution of antiviral immunity using IFNγ capture flow cytometry and ELIspot assays will also be summarized using descriptive statistics at each time point. Pairwise comparisons will be performed to compare changes of these immunological parameters from VST infusion to each time point of post-infusion measurements using paired t-tests or Wilcoxon signed-ranks tests. The normality assumption will be assessed and transformations to achieve approximate normality will be carried out if necessary. Overall survival post VST infusion will be analyzed by the Kaplan-Meier method.

Viral load levels will be correlated with immunological parameters using correlation coefficients. Longitudinal analysis is employed to model repeatedly-measured immunologic parameters. This will allow us to model patterns of immune response per patient while allowing for varying intercepts and slopes for a patient. Viral load levels will be correlated with immunological parameters using correlation coefficients. These modeling strategies will be considered exploratory in nature due to the limited patient numbers in this initial trial.

6 REPORTING REQUIREMENTS

6.1 Registration

Register all patients with PBMTC Operations Center coordinator: ACES@chla.usc.edu

Study registration procedures are described in the ACES Study Manual of Procedures (MOP).

6.2 Drug Toxicity and/or Adverse Reactions

6.2.1 Adverse events will be collected as per PBMTC standard protocols. Data on adverse experiences/toxicities regardless of seriousness must be collected for documentation purposes only for 30 days after the last VST dose with the exception of chronic GVHD which will be followed for 1 year.

6.2.2 Unexpected serious adverse events will be collected and reported as per PBMTC standard protocols until 1 year after the last VST dose.

6.3 Safety Analysis of Adverse Events Data

All patients who received T-cell infusions will be included in the safety analysis. Safety and toxicity outcomes will be summarized by dose levels in each of the two patient groups. Adverse event data and corresponding toxicity grades 30 days after T-cell infusions during long-term follow-up will be summarized in the form of tables. Incidence tables will be generated to summarize incidence of patients reporting at least one episode of each specific adverse event, incidence of adverse events causing withdrawal and incidence of serious adverse events. The total number of episodes for each event reported (Frequency Table), the severity and attribution to study therapy of each episode reported (Severity Table and Attribution Table) will also be displayed.

Listings of adverse events by patients will include the time to onset, the duration of each event, the severity of each event, and the relationship of the event to study therapy, whether it was a serious event, and whether it caused withdrawal. Safety data will be summarized for the overall patient group and by dose levels.

6.4 **Adverse Event**

An adverse event (AE) is any untoward medical occurrence regardless of causality assessment. An adverse event can be an unfavorable and unintended sign (including an abnormal laboratory finding), symptom, syndrome or disease associated with or occurring during the use of an investigational product whether or not considered related to the investigational product.

6.5 Serious Adverse Event

An adverse event is defined as a serious adverse event (SAE) when the AE

1) Results in death
2) Is considered life-threatening
3) Results in hospitalization or cause the prolongation of hospitalization
4) Results in persistent or significant incapacity or substantial disruption of the ability to conduct normal life functions
5) Leads to a congenital anomaly
6) Represents a significant medical condition which, without urgent medical intervention, would lead to one of the above outcomes.

Life-threatening means that the AE represented an immediate threat of death without medical intervention.

It is anticipated that most if not all patients on this study will be hospitalized during the course of their allogeneic HCT. Therefore, regarding the hospitalization criterion for seriousness, only AEs that clearly result in prolongation of hospitalization should be considered serious for this study

6.6 Unexpected Adverse Event

Unexpected Adverse Events are those events the nature of which, severity, or frequency are not consistent with the known or foreseeable risk of adverse events associated with the research procedures described in the informed consent document.

6.7 Other Adverse Events

Other adverse events will be identified by the PI during the evaluation of safety data. Significant adverse events of particular clinical importance, other than SAEs and those AEs leading to discontinuation of the subject from the study, will be classified as other adverse events. For each, a narrative may be written and included in the clinical study report.

Unanticipated problems include unexpected adverse events and also unexpected problems, events, or new information which are not adverse events but which indicate that research participants or others are at greater risk of harm than previously believed prior to recognition of the unanticipated problem.

6.8 Relationship to Treatment

Attribution of the event to the investigational product may be characterized as follows:

- Definitely related, clearly associated with study drug/treatment
- Probably related, likely associated with study drug/treatment

- Possibly related, may be associated with study drug or other treatment
- Unlikely to be related, or
- Definitely not related to the study drug/treatment

6.9 Severity Assessment

All AEs will be assessed for severity by the investigator. Inherent in this assessment is the medical and clinical consideration of all information surrounding the event including any medical intervention required. Each event will be assigned one of the following categories: mild, moderate, severe, or life-threatening. The criteria below may be used for any symptom not included in the grading scale. Any grade 4 (life-threatening) AE must be reported as an SAE.

Mild	Grade 1	Does not interfere with routine activities<br>Minimal level of discomfort
Moderate	Grade 2	Interferes with routine activities<br>Moderate level of discomfort
Severe	Grade 3	Unable to perform routine activities<br>Significant level of discomfort
Potentially life-threatening	Grade 4	Hospitalization or ER visit for potentially<br>life-threatening event

FDA guidelines for toxicity will be followed; however, if a subject is evaluated in an emergency room for nonlife threatening illness or symptoms (ie, visits emergency department on weekend for mild problems because the physician's office is closed), the information from that visit will be reviewed and severity of the adverse event will be assessed according to the subject's clinical signs and symptoms.

As defined by the ICH guideline for GCP, the term "severe" is often used to describe intensity (severity) of a specific event (as in mild, moderate, or severe myocardial infarction); the event itself however, may be of relatively minor medical significance (such as severe headache). This is not the same as "serious", which is based on subject/event outcome or action criteria usually associated with events that pose a threat to a subject's life or functioning. Seriousness (not severity) serves as a guide for defining regulatory reporting obligations.

7 STUDY INTERPRETATION

7.1 Aim to collect information

Since this is a phase I/II study, the primary aim will be to collect information about the safety, feasibility, and antiviral efficacy of this therapy. As secondary endpoints, we will evaluate the recovery of virus-specific immunity in patients after VST infusion and its correlation with

protection from viral reactivation/disease. This will allow us to collect data on antiviral efficacy for a future Phase II study.

7.2 **Records to be Kept**

The CHLA Study Coordinating Center will maintain a database documenting the dates and doses of therapy as well as clinical chemistries and hematologic parameters. The clinical status and occurrence of any adverse events and subsequent interventions are to be kept on all patients.

- Imaging reports
- Surgical summaries
- Autopsy summaries, where appropriate
- Informed consent documents

All required clinical evaluation records will be the responsibility of principal Investigator who will also be responsible for analysis of the clinical outcome and toxicity.
The laboratory evaluation of immunological efficacy will be the responsibility of Principal Investigator.

7.3 Reporting Requirements

The Cancer Therapy Evaluation program (CTEP) Active Version (Version 4.03) of the NCI Common Terminology Criteria for Adverse Events (CTCAE) will be utilized for AE reporting. The CTEP Active Version (Version 4.03) of the CTCAE is identified and located on the CTEP website at http://ctep.cancer.gov/protocolDevelopment/electronic_applications/ctc.htm. All appropriate treatment areas should have access to a copy of the CTEP Active Version of CTCAE (Version 4.03).

- ☐ Toxicity Grading: The criteria listed in the CTEP Active Version (Version 4.03) of the NCI Common Toxicity Criteria Scale will be used in grading toxicity.
- ☐ GVHD will be graded by the method of Przepiorka et al
- ☐ Should unanticipated toxicities arise (e.g. grade 3-4 local reactions or hepatorenal damage) they, too, will be graded by the CTEP Active Version (Version 4.03) of the NCI Common Terminology Criteria for Adverse Events (CTCAE).
- ☐ Adverse events will be collected as per PBMTC standard protocols. Data on all adverse experiences/toxicities regardless of seriousness must be collected for documentation purposes only for 30 days after the last VST dose.
- ☐ Serious adverse events will be collected and reported as per PBMTC standard protocols.
- ☐ If grade 4-5 toxicity is encountered which is definitely related to VST, the Institutional Review Board and the FDA will be notified.

7.4 **Adverse Event Reporting**

Reporting of patient serious adverse events (SAEs) will be according to standard practices and requirements. Unexpected, grades 4-5 AEs must be reported within 24 hours of knowledge of the event. Unexpected, grade 3 AEs must be reported within three business days of knowledge of the event. Other SAEs will be tracked periodically as defined in the ACES Manual of Procedures, staged according to NCI Common Terminology Criteria for Adverse Events (CTCAE), Version 4.03. The Data Monitoring Safety Committee (DMSC) will receive summary reports of all adverse experiences on at least an annual basis.

7.5 **Reporting to the IRB**

Local Site Principal Investigators will submit reports of unexpected SAEs and other unanticipated problems to local IRBs in an expedited fashion as required per institutional IRB policy. These events will be reviewed by the institutional IRBs.

7.5.1 Reporting Time Frames

Reports must be submitted to the Study Coordinating Center in the following timeframes:

Unexpected SAEs resulting in death must be reported within 24 hours of the Local Site Principal Investigator's awareness of the event.

All other SAEs and other unanticipated problems meeting the expedited reporting criteria must be reported within seven days of the Local Site Principal Investigator's awareness of the event.

All SAEs and Unanticipated Problems will be forwarded from the Study Coordinating Center to the Sponsor-Investigator for review within 72 hours of receipt. These will be reviewed and discussed with the clinical sites within seven days of the report for any SAE or Unanticipated Problem.

Upon receipt of the Study PI's assessment the sponsor will notify the appropriate regulatory groups based on the applicable regulations.

7.6 **Pregnancy Reporting**

Each pregnancy must be reported immediately (within 72 hours of identification) by email to the CHLA Study Coordinating Center. All pregnancies will be reported to the CHLA IRB. Subjects who become pregnant after Day 0 will be followed to term, and the following information will be gathered for outcome, date of delivery, health status of the mother and child including the child's gender, height and weight. Complications and or abnormalities should be reported including any premature terminations. A pregnancy is reported as an AE or SAE only when there is suspicion that the treatment may have interfered with the effectiveness of contraception or there was a serious complication in the pregnancy including a spontaneous abortion or an elective termination for medical rationale.

7.7 **IND Annual Report to the FDA**

The site PI will be responsible for the preparation of a detailed annual synopsis of clinical activity, including adverse events, for submission to the IND sponsor-investigator. Each annual report will summarize study activity for 1 year beginning approximately 3 months before the IND

FDA anniversary date. The IND sponsor-investigator's representative will notify the PI of the due date with sufficient time for the PI to assemble the required information.

7.8 **Final Report**

A report will be submitted to the FDA in accordance with ICH E3 Guideline "Structure and Content of Clinical Study Reports".

8 CLINICAL TRIAL OVERSIGHT AND MONITORING

This protocol will be conducted in accordance with the standards of practice of the PBMTC and each study site.

This protocol will be monitored in accordance with current Data Safety Monitoring Committee (DMSC) charter for investigator-initiated studies performed in the PBMTC network.

The conduct of this clinical trial will be evaluated in accordance with PBMTC policies.

8.1 Safety monitoring

Safety monitoring will be conducted throughout the study; therefore safety concerns will be identified by continuous review of the data by the PI, clinic staff, and Data Safety Monitoring Committee (DSMC).

8.2 **Data Safety Monitoring Committee (DSMC)**

This study will be centrally reviewed and followed by the Data Safety and Monitoring Committee (DSMC) of the Pediatric Blood and Marrow Transplant Consortium (PBMTC). The DSMC is a standing committee, composed of a chair, patient advocate, biostatistician, nurse representative and two bone marrow transplant physicians with procedures and processes as defined in the PBMTC DSMC Charter. The DSMC will review the study protocol prior to study activation and IRB review, and will continue to review the study on a regular basis according to the committee rules.

The DSMC will meet at regular intervals to review all adverse events and deaths and determine whether any patient safety problems necessitate protocol modifications or discontinuation of the trial. The DSMC will also meet on an *ad hoc* basis if unexpected safety events occur that may necessitate study suspension or closure. The DSMC will discontinue the review of outcomes when this protocol is closed to accrual.

Before each regularly scheduled DSMC meeting, the CHLA Study Coordinating Center will submit a report including tabular summaries of all SAEs and deaths on study to date. The report will also include a brief summary of each previously unreported SAE and death, including an assessment of whether the event was unexpected or related to the study.
If the DSMC recommends protocol or informed consent changes during the study, the recommendations will be reviewed by the Protocol Co-Chairs and incorporated into the protocol as deemed appropriate. The protocol with incorporated changes will be distributed to the participating study sites after approval by the Central IRB (CHLA). It is the responsibility of each study site PI to forward the distributed communications from the DSMC to their local IRB and provide updated approval information to the Central Site (CHLA)

8.3 Study Monitoring

The Local Site Investigators will permit study-related monitoring visits by representatives of the sponsor or designees, and regulatory inspections(s) (e.g., FDA) to ensure proper conduct of the study and compliance with all FDA safety reporting requirements. Access will be provided to the facilities where the study took place, to source documents, to CRFs, and to all other study documents.

Clinical site monitoring is conducted to ensure that the rights of human subjects are protected, that the study is implemented in accordance with the protocol and/or other operating procedures, and that the quality and integrity of study data and data collection methods are maintained. The monitor will evaluate study processes and documentation based on FDA regulations and the International Conference on Harmonisation (ICH), E6: Good Clinical Practice guidelines (GCP) and 21CFR 312.

APPENDIX I: CHRONIC GVHD DEFINITIONS & SCORING TABLE

CHRONIC GVHD DEFINITIONS and SCORING TABLE

Check all that apply	Score 0 – None	Score 1 - Mild	Score 2 - Moderate	Score 3 - Severe
**Skin:**<br>*Clinical features:*<br>☐ Maculopapular rash<br>☐ Lichen planus-like features<br>☐ Papulosquamous lesions or ichthyosis<br>☐ Hyperpigmentation<br>☐ Hypopigmentation<br>☐ Keratosis pilaris<br>☐ Erythema<br>☐ Erythroderma<br>☐ Sclerotic features<br>☐ Poikiloderma<br>☐ Pruritus<br>☐ Hair Involvement<br>☐ Nail Involvement<br>% BSA involved ______%	☐ No symptoms	☐ < 18% BSA with disease signs but NO sclerotic features	☐ 19-50% BSA,<br>☐ Involvement with superficial sclerotic features "not hidebound" (able to pinch)	☐ > 50% BSA<br>☐ Deep sclerotic features "hidebound" (unable to pinch)<br>☐ Impaired mobility, ulceration or severe pruritis
**Mouth:**	☐ No symptoms	☐ Mild symptoms with disease signs but not limiting oral intake significantly	☐ Moderate symptoms with disease signs WITH partial limitation of oral intake	☐ Severe symptoms with disease signs WITH major limitation of oral intake
**Eyes:**<br>Mean tear test (mm):<br>> 10<br>6-10<br>≤ 5<br>Not done	☐ No symptoms	☐ Mild dry eyes symptoms not affecting ADL (requiring eye drops ≤ 3 x per day)<br>☐ Asymptomatic signs of keratoconjunctivitis sicca	☐ Moderate dry eyes symptoms partially affecting ADL (requiring eyedrops > 3 x per day or punctual plugs) WITHOUT vision impairment	☐ Severe dry eyes symptoms significantly affecting ADL (special eyewear to relieve pain)<br>☐ Unable to work because of ocular symptoms<br>☐ Loss of vision caused by keratoconjuctivitis sicca
**Lungs‡**<br><br>FEV1 ______%<br><br>DLCO ______	☐ No symptoms<br>☐ FEV1> 80%<br>☐ LFS = 2	☐ Mild symptoms (shortness of breath after climbing one flight of steps)<br>☐ FEV1 60-78%<br>☐ LFS 3-5	☐ Moderate symptoms (shortness of breath after walking on flat ground)<br>☐ FEV1 40-51%<br>☐ LFS 6-9	☐ Severe symptoms (shortness of breath at rest; requiring O2)<br>☐ FEV1 ≤ 39%<br>☐ LFS 10-12
**GI Tract:**	☐ No symptoms	☐ Symptoms such as dysphagia, anorexia, nausea, vomiting, abdominal pain or diarrhea without significant weight loss (< 5%)	☐ Symptoms associated with mild to moderate weight loss (5 – 15%)	☐ Symptoms associated with significant weight loss > 15%<br>☐ Requires nutritional supplement for most caloric needs<br>☐ Esophageal dilation
**Liver:**	☐ Normal LFT	☐ Elevated Bilirubin, AP*, AST or ALT < 2 x ULN	☐ Bilirubin > 3 mg/dl or Bilirubin enzymes 2-5 x ULN	☐ Bilirubin or enzymes > 5 x ULN
**Genital Tract:**	☐ No symptoms	☐ Symptomatic with mild signs on exam AND no effect on coitus and minimal discomfort with gynecological exam	☐ Symptomatic with moderate signs on exam AND with mild dyspareunia or discomfort with gynecological exam	☐ Symptomatic WITH advanced signs (stricture, labial agglutination or severe ulceration) AND severe pain with coitus or inability to insert vaginal speculum
**Joints and Fascia:**	☐ No symptoms	☐ Mild tightness of arms or legs, normal or mild	☐ Tightness of arms or legs<br>☐ Joint contractures,	☐ Contractures WITH significant decrease of ROM

15

APPENDIX II: ADVERSE EVENT MANAGEMENT FOLLOWING VST INFUSIONS

1. PRINCIPLE

This SOP encompasses the suggested management of reactions to Virus-Specific T-lymphocyte (VST) infusions under the ACES protocol (PBMTC SUP1701). Like other cellular therapy products, allogeneic VST can result in adverse reactions during or after infusion. The adverse reaction needs to be recognized, the patient must receive appropriate treatment, and the adverse reaction should be properly reported. This protocol is meant to guide study clinicians in the standard workup of potential Adverse Events (AE's), and does not replace clinical judgement.

2. PURPOSE

This SOP describes the procedure for identification, clinical management, and reporting of adverse reactions to T-cell infusions.

3. POLICIES

3.1 Scope

This SOP covers infusion of VSTs in the inpatient and outpatient setting. It applies to adverse reactions occurring early (within 24 hours of T-cell infusion) or late (more than 24 hours after T-cell infusion).

3.2 Personnel

Procedures described in this SOP will be carried out by the local study teams, including site PIs and co-investigators who have been trained in the study procedures and reviewed all relevant study material.

4. SPECIMEN

Specimens required for clinical care will be collected and processed according to standard Laboratory policies and procedures.

5. MATERIALS

Reagents, supplies and equipment needed for clinical care will be acquired and used according to Hospital policies and procedures.

6. SAFETY

Standard hospital safety policies apply to all clinical care.

All adverse events identification and reporting will be consistent with both PBMTC and local policies and procedures regarding safety and patient confidentiality.

7. RECORDS/FORMS/DOCUMENTS

All adverse events will be documented in the patient's medical record according to Hospital policies and procedures, and reported to the PBMTC study team within the required time frame, depending on the nature and seriousness of the AE.

8. QUALITY CONTROL

In addition to the T-cell-infusion-specific reporting procedures described below, all adverse reactions to T-cell infusion will be reported as described in the ACES Study Manual. The

reactions should be reviewed by the local study PIs to determine if any additional monitoring or modifications to standard operating procedures are necessary.

9. PROCEDURE

9.1. Early Reactions

9.1.1 Early reactions are usually related to the cryopreserved components

9.1.2 Common, mild complications from infusion of T-cell products include:

- mild increase in blood pressure not requiring intervention
- mild headache
- mild flushing
- slight slowing of the heart rate
- bad taste in the mouth.

9.1.3 Severe immediate reactions are rare after T-cell infusions, but may include:

- acute pulmonary edema and dyspnea
- persistent nausea and vomiting
- increase in blood pressure that does not respond to medication with standard doses of furosemide and/or nifedipine
- fever
- anaphylactic shock
- bradycardia or cardiac arrest

9.1.4 Management of Early Reactions

Most common, mild early reactions do not required intervention. In the event of a severe reaction, the patient should be immediately transferred to the BMTU or the ICU for intensive monitoring and intervention, which usually include BP support, steroids, and management of anaphylaxis.

9.2. Late Reactions

9.2.1 Late reactions to T-cell infusion may be related to a T-cell engraftment syndrome or cytokine release syndrome (CRS), tumor lysis syndrome, and rarely to contamination of the product.

9.2.2 Symptoms and signs of late complications from infusion of T-cell products may include:

- **Systemic**
    - Fever
    - Malaise, fatigue
    - Myalgias and arthralgias
    - skin rash mimicking acute GVHD
    - general feeling of unwellness

- disseminated intravascular coagulation (DIC) +/- bleeding
- macrophage activation syndrome/hemophagocytoic lymphohistiocytosis (HLH)
- anorexia, nausea, vomiting, diarrhea

- **Cardiorespiratory**
  - tachycardia
  - blood pressure changes (either hyper- or hypotension)
  - acute pulmonary edema, dyspnea, hypoxia
  - pulmonary infiltrates
  - capillary leak syndrome
  - cardiac dysfunction
  - stress cardiomyopathy (Takotsubo cardiomyopathy)
  - adult respiratory distress syndrome (ARDS)

- **Hepatic and Renal**
  - weight gain
  - renal impairment
  - azotemia
  - hyperuricemia
  - hepatic impairment – transaminitis, hyperbilirubinemia

- **Neurological**
  - headache
  - mental status changes
  - confusion
  - delirium
  - hallucinations
  - altered gait
  - seizures
  - encephalopathy
  - mild encephalopathy with reversible splenial lesion syndrome (MERS)

9.2.3 Management of Late Reactions

9.2.3.1. The development of a severe late reaction necessitates immediate notification of the local study Principal Investigator (PI) AND (if an outpatient) transfer to the hospital for an urgent medical assessment. It is important to evaluate the patient and initiate therapy as quickly as possible since rapid deterioration is possible.

9.2.3.2 **Biomarkers**: Circulating cytokine levels can serve as biomarkers to diagnose and potentially quantify syndrome severity, and should be obtained promptly if CRS is suspected.

In patients who are critically ill and/or who have substantial viral loads (≥ log 6) at the time of enrollment, baseline measurement of serum cytokines and inflammatory markers prior to infusion is advisable.

-IL-6 signaling is major component of severe CRS
-If possible, cytokine levels should be sent to Viracor prior to starting any anticytokine directed therapy
-CRP serves as a reliable surrogate for IL-6 bioactivity and levels should be sent pre and post therapy to monitor response
-Ferritin may also be used in conjunction with CRP monitoring

9.2.3.2. The immediate assessment and treatment may also include:

- Intensive monitoring (BP, cardiovascular monitoring, pulse oximetry or ABG)
- CXR or CT of chest
- Steroids – single dose. See 9.2.3.3
- Microbiological studies (especially blood cultures and virus studies) and initiation of broad-spectrum antibiotics +/- antivirals should be considered.
-Blood pressure/Cardiovascular support- early institution of inotropic medications. Limit fluid resuscitation to the minimum volume needed to support blood pressure, no more than 20 mL/kg, if possible.
- Fluid management/Renal support- Aggressive diuresis. Institute early involvement of the renal service for fluid management especially if evidence of capillary leak syndrome. Consider infusion of 25% albumin if serum albumin is < 3 g/dL. See Fluid Management SOP, SCTCP-37.
- Respiratory support – supplemental oxygen therapy should be initiated for all hypoxemic patients. Patients also may require intubation and mechanical ventilation.
-Management of tumor lysis syndrome if there is laboratory evidence it is occurring.

9.2.3.3. Treatment of the cytokine release syndrome or other T-cell associated adverse reactions must be discussed with the local study PI prior to starting steroids. If the situation is emergent, the first dose of steroids can be given before consultation with the PI.

Notable suggested changes to standard management include the following:

- When managing CRS, anti-IL-6 directed therapy should be initiated before anti-TNFa directed therapy.
- Following investigational VST infusion, steroids should not be considered first line therapy and anti-IL-6 directed therapy MAY be given without first starting steroids. This should only be done in consultation with the study PI.
- Caution is required when using Tocilizumab in the setting of hepatic impairment.

9.2.3.4. If treatment with a biological agent is necessary, the order of priority will usually be:

- Tocilizumab
- Infliximab(anti-TNFa)
- Etanercept (soluble TNFa receptor inhibitor)
- Anti-thymocyte globulin (ATG)
- Alemtuzumab (Campath)

Drug	Dosing	Duration
Infliximab	10 mg/kg IV	One dose, may give second 3-4 days later
Etanercept	0.4 mg/kg (maximum 25 mg) IV (first dose) followed by SC	One dose, may give second 3-4 days later
Tocilizumab	<30 kg: 8-mg/kg IV<br>≥30 kg: 4 mg/kg (maximum 800mg) IV	One dose, with repeat dosing if no improvement observed within 24-48 hours.
ATG, equine	30 mg/kg IV	Daily for 3 days
ATG, rabbit	3 mg/kg IV	Daily for 3 days
Alemtuzumab	10 mg (note: not per kg) IV	Daily for 3 days

9.3. The local study PI should be notified immediately of any reactions described in 9.1 and 9.2.

10. INTERPRETATION

Interpretation of clinical situations discussed in this SOP will be done by the medical and nursing clinical teams.

11. RESULTS REPORTING

See ACES Study Manual for reporting requirements.

12. LIMITATIONS

Individual patient care may sometimes have to be different from that set out in the guidelines because of a relevant patient factor, which should be discussed with the local study PI and team.

This procedure does not cover every possible scenario, and consultation with the study PI or Critical Care Medicine is recommended in unusual or emergency situations

References

1. Saglio F, Hanley PJ, Bollard CM. The time is now: moving toward virus-specific T cells after allogeneic hematopoietic stem cell transplantation as the standard of care. *Cytotherapy.* 2014;16(2):149-159.
2. Dahi PB, Perales MA, Devlin SM, et al. Incidence, nature and mortality of cytomegalovirus infection after double-unit cord blood transplant. *Leuk Lymphoma.* 2015;56(6):1799-1805.
3. Walker CM, van Burik JA, De For TE, Weisdorf DJ. Cytomegalovirus infection after allogeneic transplantation: comparison of cord blood with peripheral blood and marrow graft sources. *Biol Blood Marrow Transplant.* 2007;13(9):1106-1115.
4. Boeckh M, Leisenring W, Riddell SR, et al. Late cytomegalovirus disease and mortality in recipients of allogeneic hematopoietic stem cell transplants: importance of viral load and T-cell immunity. *Blood.* 2003;101(2):407-414.
5. Odek C, Kendirli T, Dogu F, et al. Patients with primary immunodeficiencies in pediatric intensive care unit: outcomes and mortality-related risk factors. *J Clin Immunol.* 2014;34(3):309-315.
6. Buckley RH. Transplantation of hematopoietic stem cells in human severe combined immunodeficiency: longterm outcomes. *Immunol Res.* 2011;49(1-3):25-43.
7. Gottschalk S, Rooney CM, Heslop HE. Post-transplant lymphoproliferative disorders. *Annu Rev Med.* 2005;56:29-44.
8. Myers GD, Krance RA, Weiss H, et al. Adenovirus infection rates in pediatric recipients of alternate donor allogeneic bone marrow transplants receiving either antithymocyte globulin (ATG) or alemtuzumab (Campath). *Bone Marrow Transplant.* 2005;36(11):1001-1008.
9. Lujan-Zilbermann J, Benaim E, Tong X, Srivastava DK, Patrick CC, DeVincenzo JP. Respiratory virus infections in pediatric hematopoietic stem cell transplantation. *Clin Infect Dis.* 2001;33(7):962-968.
10. Crooks BN, Taylor CE, Turner AJ, et al. Respiratory viral infections in primary immune deficiencies: significance and relevance to clinical outcome in a single BMT unit. *Bone Marrow Transplant.* 2000;26(10):1097-1102.
11. Boeckh M, Ljungman P. How we treat cytomegalovirus in hematopoietic cell transplant recipients. *Blood.* 2009;113(23):5711-5719.
12. Cohen JI. Primary Immunodeficiencies Associated with EBV Disease. *Curr Top Microbiol Immunol.* 2015;390(Pt 1):241-265.
13. Hutspardol S, Essa M, Richardson S, et al. Significant Transplantation-Related Mortality from Respiratory Virus Infections within the First One Hundred Days in Children after Hematopoietic Stem Cell Transplantation. *Biol Blood Marrow Transplant.* 2015;21(10):1802-1807.
14. Sellar RS, Peggs KS. Management of multidrug-resistant viruses in the immunocompromised host. *Br J Haematol.* 2012;156(5):559-572.
15. Rezvani AR, Maloney DG. Rituximab resistance. *Best Pract Res Clin Haematol.* 2011;24(2):203-216.

16. Maloney DG, Smith B, Rose A. Rituximab: mechanism of action and resistance. *Seminars in oncology*. 2002;29(1 Suppl 2):2-9.
17. Pai SY, Logan BR, Griffith LM, et al. Transplantation outcomes for severe combined immunodeficiency, 2000-2009. *N Engl J Med.* 2014;371(5):434-446.
18. Haddad E, Leroy S, Buckley RH. B-cell reconstitution for SCID: should a conditioning regimen be used in SCID treatment? *J Allergy Clin Immunol.* 2013;131(4):994-1000.
19. Gennery AR, Slatter MA, Grandin L, et al. Transplantation of hematopoietic stem cells and long-term survival for primary immunodeficiencies in Europe: entering a new century, do we do better? *J Allergy Clin Immunol.* 2010;126(3):602-610 e601-611.
20. Hanley PJ, Bollard CM. Controlling cytomegalovirus: helping the immune system take the lead. *Viruses.* 2014;6(6):2242-2258.
21. Bollard CM. Improving T-cell therapy for epstein-barr virus lymphoproliferative disorders. *J Clin Oncol.* 2013;31(1):5-7.
22. Papadopoulou A, Gerdemann U, Katari UL, et al. Activity of broad-spectrum T cells as treatment for AdV, EBV, CMV, BKV, and HHV6 infections after HSCT. *Science translational medicine.* 2014;6(242):242ra283.
23. Drew WL, Paya CV, Emery V. Cytomegalovirus (CMV) resistance to antivirals. *Am J Transplant.* 2001;1(4):307-312.
24. Boeckh M, Nichols WG, Papanicolaou G, Rubin R, Wingard JR, Zaia J. Cytomegalovirus in hematopoietic stem cell transplant recipients: Current status, known challenges, and future strategies. *Biol Blood Marrow Transplant.* 2003;9(9):543-558.
25. Sylwester AW, Mitchell BL, Edgar JB, et al. Broadly targeted human cytomegalovirus-specific CD4+ and CD8+ T cells dominate the memory compartments of exposed subjects. *J Exp Med.* 2005;202(5):673-685.
26. Biron KK. Antiviral drugs for cytomegalovirus diseases. *Antiviral Res.* 2006;71(2-3):154-163.
27. Young LS, Rickinson AB. Epstein-Barr virus: 40 years on. *Nature reviews Cancer.* 2004;4(10):757-768.
28. Cohen JM, Cooper N, Chakrabarti S, et al. EBV-related disease following haematopoietic stem cell transplantation with reduced intensity conditioning. *Leuk Lymphoma.* 2007;48(2):256-269.
29. Bollard CM, Rooney CM, Heslop HE. T-cell therapy in the treatment of post-transplant lymphoproliferative disease. *Nat Rev Clin Oncol.* 2012;9(9):510-519.
30. Kuehnle I, Huls MH, Liu Z, et al. CD20 monoclonal antibody (rituximab) for therapy of Epstein-Barr virus lymphoma after hemopoietic stem-cell transplantation. *Blood.* 2000;95(4):1502-1505.
31. Smith MR. Rituximab (monoclonal anti-CD20 antibody): mechanisms of action and resistance. *Oncogene.* 2003;22(47):7359-7368.
32. de Lima CR, Mirandolli TB, Carneiro LC, et al. Prolonged respiratory viral shedding in transplant patients. *Transpl Infect Dis.* 2014;16(1):165-169.
33. Myers GD, Bollard CM, Wu MF, et al. Reconstitution of adenovirus-specific cell-mediated immunity in pediatric patients after hematopoietic stem cell transplantation. *Bone Marrow Transplant.* 2007;39(11):677-686.
34. Lugthart G, Oomen MA, Jol-van der Zijde CM, et al. The effect of cidofovir on adenovirus plasma DNA levels in stem cell transplantation recipients without T cell reconstitution. *Biol Blood Marrow Transplant.* 2015;21(2):293-299.

35. Neofytos D, Ojha A, Mookerjee B, et al. Treatment of adenovirus disease in stem cell transplant recipients with cidofovir. *Biol Blood Marrow Transplant.* 2007;13(1):74-81.
36. Camargo JF, Morris MI, Abbo LM, et al. The use of brincidofovir for the treatment of mixed dsDNA viral infection. *Journal of clinical virology : the official publication of the Pan American Society for Clinical Virology.* 2016;83:1-4.
37. Kinchington PR, Araullo-Cruz T, Vergnes JP, Yates K, Gordon YJ. Sequence changes in the human adenovirus type 5 DNA polymerase associated with resistance to the broad spectrum antiviral cidofovir. *Antiviral Res.* 2002;56(1):73-84.
38. Feuchtinger T, Lucke J, Hamprecht K, et al. Detection of adenovirus-specific T cells in children with adenovirus infection after allogeneic stem cell transplantation. *Br J Haematol.* 2005;128(4):503-509.
39. Cwynarski K, Ainsworth J, Cobbold M, et al. Direct visualization of cytomegalovirus-specific T-cell reconstitution after allogeneic stem cell transplantation. *Blood.* 2001;97(5):1232-1240.
40. Heslop HE, Slobod KS, Pule MA, et al. Long-term outcome of EBV-specific T-cell infusions to prevent or treat EBV-related lymphoproliferative disease in transplant recipients. *Blood.* 2010;115(5):925-935.
41. Leen AM, Christin A, Myers GD, et al. Cytotoxic T lymphocyte therapy with donor T cells prevents and treats adenovirus and Epstein-Barr virus infections after haploidentical and matched unrelated stem cell transplantation. *Blood.* 2009;114(19):4283-4292.
42. Leen AM, Myers GD, Sili U, et al. Monoculture-derived T lymphocytes specific for multiple viruses expand and produce clinically relevant effects in immunocompromised individuals. *Nat Med.* 2006;12(10):1160-1166.
43. Rooney CM, Smith CA, Ng CY, et al. Infusion of cytotoxic T cells for the prevention and treatment of Epstein-Barr virus-induced lymphoma in allogeneic transplant recipients. *Blood.* 1998;92(5):1549-1555.
44. Bao L, Cowan MJ, Dunham K, et al. Adoptive immunotherapy with CMV-specific cytotoxic T lymphocytes for stem cell transplant patients with refractory CMV infections. *J Immunother.* 2012;35(3):293-298.
45. Koehne G, Hasan A, Doubrovina E, et al. Immunotherapy with Donor T Cells Sensitized with Overlapping Pentadecapeptides for Treatment of Persistent Cytomegalovirus Infection or Viremia. *Biol Blood Marrow Transplant.* 2015;21(9):1663-1678.
46. Doubrovina E, Oflaz-Sozmen B, Prockop SE, et al. Adoptive immunotherapy with unselected or EBV-specific T cells for biopsy-proven EBV+ lymphomas after allogeneic hematopoietic cell transplantation. *Blood.* 2012;119(11):2644-2656.
47. Walter EA, Greenberg PD, Gilbert MJ, et al. Reconstitution of cellular immunity against cytomegalovirus in recipients of allogeneic bone marrow by transfer of T-cell clones from the donor. *N Engl J Med.* 1995;333(16):1038-1044.
48. Peggs KS, Verfuerth S, Pizzey A, et al. Adoptive cellular therapy for early cytomegalovirus infection after allogeneic stem-cell transplantation with virus-specific T-cell lines. *The Lancet.* 2003;362(9393):1375-1377.
49. Micklethwaite K, Hansen A, Foster A, et al. Ex vivo expansion and prophylactic infusion of CMV-pp65 peptide-specific cytotoxic T-lymphocytes following allogeneic hematopoietic stem cell transplantation. *Biol Blood Marrow Transplant.* 2007;13(6):707-714.

50. Einsele HR, E.; Rufer, N.; Sinzger, C.; Riegler, S.; Loffler, J.; Grigoleit, U.; Moris, A.; Rammensee, H.; Kanz, L.; Kleihauer, A.; Frank, F.; Jahn, G.; Hebart, H. Infusion of cytomegalovirus (CMV)-specific T cells for the treatment of CMV infection not responding to antiviral chemotherapy. *Blood.* 2002;99(11):3916-3922.
51. Qasim W, Gilmour K, Zhan H, et al. Interferon-gamma capture T cell therapy for persistent Adenoviraemia following allogeneic haematopoietic stem cell transplantation. *Br J Haematol.* 2013;161(3):449-452.
52. Neudorfer J, Schmidt B, Huster KM, et al. Reversible HLA multimers (Streptamers) for the isolation of human cytotoxic T lymphocytes functionally active against tumor- and virus-derived antigens. *J Immunol Methods.* 2007;320(1-2):119-131.
53. Feuchtinger T, Opherk K, Bethge WA, et al. Adoptive transfer of pp65-specific T cells for the treatment of chemorefractory cytomegalovirus disease or reactivation after haploidentical and matched unrelated stem cell transplantation. *Blood.* 2010;116(20):4360-4367.
54. Peggs KS, Thomson K, Samuel E, et al. Directly selected cytomegalovirus-reactive donor T cells confer rapid and safe systemic reconstitution of virus-specific immunity following stem cell transplantation. *Clin Infect Dis.* 2011;52(1):49-57.
55. Creidy R, Moshous D, Touzot F, et al. Specific T cells for the treatment of cytomegalovirus and/or adenovirus in the context of hematopoietic stem cell transplantation. *J Allergy Clin Immunol.* 2016;138(3):920-924 e923.
56. Cobbold M, Khan N, Pourgheysari B, et al. Adoptive transfer of cytomegalovirus-specific CTL to stem cell transplant patients after selection by HLA-peptide tetramers. *J Exp Med.* 2005;202(3):379-386.
57. Schmitt A, Tonn T, Busch DH, et al. Adoptive transfer and selective reconstitution of streptamer-selected cytomegalovirus-specific CD8+ T cells leads to virus clearance in patients after allogeneic peripheral blood stem cell transplantation. *Transfusion.* 2011;51(3):591-599.
58. Rooney CM, Smith CA, Ng CY, et al. Use of gene-modified virus-specific T lymphocytes to control Epstein-Barr-virus-related lymphoproliferation. *Lancet.* 1995;345(8941):9-13.
59. Heslop HE, Ng CY, Li C, et al. Long-term restoration of immunity against Epstein-Barr virus infection by adoptive transfer of gene-modified virus-specific T lymphocytes. *Nat Med.* 1996;2(5):551-555.
60. Comoli P, Labirio M, Basso S, et al. Infusion of autologous Epstein-Barr virus (EBV)-specific cytotoxic T cells for prevention of EBV-related lymphoproliferative disorder in solid organ transplant recipients with evidence of active virus replication. *Blood.* 2002;99(7):2592-2598.
61. Gustafsson A, Levitsky V, Zou JZ, et al. Epstein-Barr virus (EBV) load in bone marrow transplant recipients at risk to develop posttransplant lymphoproliferative disease: prophylactic infusion of EBV-specific cytotoxic T cells. *Blood.* 2000;95(3):807-814.
62. Comoli P, Basso S, Zecca M, et al. Preemptive therapy of EBV-related lymphoproliferative disease after pediatric haploidentical stem cell transplantation. *Am J Transplant.* 2007;7(6):1648-1655.
63. Moosmann A, Bigalke I, Tischer J, et al. Effective and long-term control of EBV PTLD after transfer of peptide-selected T cells. *Blood.* 2010;115(14):2960-2970.

64. Icheva V, Kayser S, Wolff D, et al. Adoptive transfer of epstein-barr virus (EBV) nuclear antigen 1-specific t cells as treatment for EBV reactivation and lymphoproliferative disorders after allogeneic stem-cell transplantation. *J Clin Oncol.* 2013;31(1):39-48.
65. Uhlin M, Okas M, Gertow J, Uzunel M, Brismar TB, Mattsson J. A novel haplo-identical adoptive CTL therapy as a treatment for EBV-associated lymphoma after stem cell transplantation. *Cancer Immunol Immunother.* 2010;59(3):473-477.
66. Wynn RF, Arkwright PD, Haque T, et al. Treatment of Epstein-Barr-virus-associated primary CNS B cell lymphoma with allogeneic T-cell immunotherapy and stem-cell transplantation. *The lancet oncology.* 2005;6(5):344-346.
67. Feuchtinger T, Matthes-Martin S, Richard C, et al. Safe adoptive transfer of virus-specific T-cell immunity for the treatment of systemic adenovirus infection after allogeneic stem cell transplantation. *Br J Haematol.* 2006;134(1):64-76.
68. Gerdemann U, Vera JF, Rooney CM, Leen AM. Generation of multivirus-specific T cells to prevent/treat viral infections after allogeneic hematopoietic stem cell transplant. *J Vis Exp.* 2011(51).
69. Gerdemann U, Christin AS, Vera JF, et al. Nucleofection of DCs to generate Multivirus-specific T cells for prevention or treatment of viral infections in the immunocompromised host. *Mol Ther.* 2009;17(9):1616-1625.
70. Gerdemann U, Keirnan JM, Katari UL, et al. Rapidly generated multivirus-specific cytotoxic T lymphocytes for the prophylaxis and treatment of viral infections. *Mol Ther.* 2012;20(8):1622-1632.
71. Naik S, Nicholas SK, Martinez CA, et al. Adoptive immunotherapy for primary immunodeficiency disorders with virus-specific T lymphocytes. *J Allergy Clin Immunol.* 2016.
72. Hanley PJ, Keller MD, Martin Manso M, et al. A Phase 1 Perspective: Multivirus-Specific T-cells from both Cord Blood and Bone Marrow Transplant Donors. *Cytotherapy.* 2016;18(6):S8.
73. Bollard CM, Heslop HE. T cells for viral infections after allogeneic hematopoietic stem cell transplant. *Blood.* 2016;127(26):3331-3340.
74. Vickers MA, Wilkie GM, Robinson N, et al. Establishment and operation of a Good Manufacturing Practice-compliant allogeneic Epstein-Barr virus (EBV)-specific cytotoxic cell bank for the treatment of EBV-associated lymphoproliferative disease. *Br J Haematol.* 2014;167(3):402-410.
75. Hanley PJ, Melenhorst JJ, Nikiforow S, et al. CMV-specific T cells generated from naive T cells recognize atypical epitopes and may be protective in vivo. *Science translational medicine.* 2015;7(285):285ra263.
76. Hanley PJ, Cruz CR, Savoldo B, et al. Functionally active virus-specific T cells that target CMV, adenovirus, and EBV can be expanded from naive T-cell populations in cord blood and will target a range of viral epitopes. *Blood.* 2009;114(9):1958-1967.
77. Fabre C, Koscielny S, Mohty M, et al. Younger donor's age and upfront tandem are two independent prognostic factors for survival in multiple myeloma patients treated by tandem autologous-allogeneic stem cell transplantation: a retrospective study from the Societe Francaise de Greffe de Moelle et de Therapie Cellulaire (SFGM-TC). *Haematologica.* 2012;97(4):482-490.

78. O'Reilly RJ, Prockop S, Hasan AN, Koehne G, Doubrovina E. Virus-specific T-cell banks for 'off the shelf' adoptive therapy of refractory infections. *Bone Marrow Transplant.* 2016.
79. Haque T, Wilkie GM, Jones MM, et al. Allogeneic cytotoxic T-cell therapy for EBV-positive posttransplantation lymphoproliferative disease: results of a phase 2 multicenter clinical trial. *Blood.* 2007;110(4):1123-1131.
80. Haque T, Taylor C, Wilkie GM, et al. Complete regression of posttransplant lymphoproliferative disease using partially HLA-matched Epstein Barr virus-specific cytotoxic T cells. *Transplantation.* 2001;72(8):1399-1402.
81. Haque T, Amlot PL, Helling N, et al. Reconstitution of EBV-specific T cell immunity in solid organ transplant recipients. *J Immunol.* 1998;160(12):6204-6209.
82. Barker JN, Doubrovina E, Sauter C, et al. Successful treatment of EBV-associated posttransplantation lymphoma after cord blood transplantation using third-party EBV-specific cytotoxic T lymphocytes. *Blood.* 2010;116(23):5045-5049.
83. Leen AM, Bollard CM, Mendizabal AM, et al. Multicenter study of banked third-party virus-specific T cells to treat severe viral infections after hematopoietic stem cell transplantation. *Blood.* 2013;121(26):5113-5123.
84. Cruz CR, Hanley PJ, Liu H, et al. Adverse events following infusion of T cells for adoptive immunotherapy: a 10-year experience. *Cytotherapy.* 2010;12(6):743-749.
85. Wang W, Huang H, Halagan M, et al. Chromosome Y-encoded antigens associate with acute graft-versus-host disease in sex-mismatched stem cell transplant. *Blood Adv.* 2018;2(19):2419-2429.
86. Rufer N, Wolpert E, Helg C, et al. HA-1 and the SMCY-derived peptide FIDSYICQV (H-Y) are immunodominant minor histocompatibility antigens after bone marrow transplantation. *Transplantation.* 1998;66(7):910-916.
87. Rosinski KV, Fujii N, Mito JK, et al. DDX3Y encodes a class I MHC-restricted H-Y antigen that is expressed in leukemic stem cells. *Blood.* 2008;111(9):4817-4826.
88. Ofran Y, Kim HT, Brusic V, et al. Diverse patterns of T-cell response against multiple newly identified human Y chromosome-encoded minor histocompatibility epitopes. *Clin Cancer Res.* 2010;16(5):1642-1651.
89. Takami A, Sugimori C, Feng X, et al. Expansion and activation of minor histocompatibility antigen HY-specific T cells associated with graft-versus-leukemia response. *Bone Marrow Transplant.* 2004;34(8):703-709.
90. James E, Chai JG, Dewchand H, Macchiarulo E, Dazzi F, Simpson E. Multiparity induces priming to male-specific minor histocompatibility antigen, HY, in mice and humans. *Blood.* 2003;102(1):388-393.
91. Melenhorst JJ, Leen AM, Bollard CM, et al. Allogeneic virus-specific T cells with HLA alloreactivity do not produce GVHD in human subjects. *Blood.* 2010;116(22):4700-4702.
92. Ciurea SO, Thall PF, Wang X, et al. Donor-specific anti-HLA Abs and graft failure in matched unrelated donor hematopoietic stem cell transplantation. *Blood.* 2011;118(22):5957-5964.
93. Ochs HD, Filipovich AH, Veys P, Cowan MJ, Kapoor N. Wiskott-Aldrich syndrome: diagnosis, clinical and laboratory manifestations, and treatment. *Biol Blood Marrow Transplant.* 2009;15(1 Suppl):84-90.
94. Notarangelo LD, Kim MS, Walter JE, Lee YN. Human RAG mutations: biochemistry and clinical implications. *Nat Rev Immunol.* 2016;16(4):234-246.

95. Milner JD, Vogel TP, Forbes L, et al. Early-onset lymphoproliferation and autoimmunity caused by germline STAT3 gain-of-function mutations. *Blood.* 2015;125(4):591-599.
96. Coulter TI, Chandra A, Bacon CM, et al. Clinical spectrum and features of activated phosphoinositide 3-kinase delta syndrome: A large patient cohort study. *J Allergy Clin Immunol.* 2016.
97. Bunin N, Guzikowski V, Rand ER, et al. Solid organ transplants following hematopoietic stem cell transplant in children. *Pediatric transplantation.* 2010;14(8):1030-1035.
98. Uhlin M, Gertow J, Uzunel M, et al. Rapid salvage treatment with virus-specific T cells for therapy-resistant disease. *Clin Infect Dis.* 2012;55(8):1064-1073.
99. Bonilla FA, Khan DA, Ballas ZK, et al. Practice parameter for the diagnosis and management of primary immunodeficiency. *J Allergy Clin Immunol.* 2015.
100. Bousfiha AA, Jeddane L, Ailal F, et al. A phenotypic approach for IUIS PID classification and diagnosis: guidelines for clinicians at the bedside. *J Clin Immunol.* 2013;33(6):1078-1087.
101. Martin PJ, Rizzo JD, Wingard JR, et al. First- and second-line systemic treatment of acute graft-versus-host disease: recommendations of the American Society of Blood and Marrow Transplantation. *Biol Blood Marrow Transplant.* 2012;18(8):1150-1163.
102. Papadopoulou A, Krance RA, Allen CE, et al. Systemic inflammatory response syndrome after administration of unmodified T lymphocytes. *Mol Ther.* 2014;22(6):1134-1138.
103. Rothman KJ. No adjustments are needed for multiple comparisons. *Epidemiology.* 1990;1(1):43-46.
