# Supplementary material for: Antiviral cellular therapy for enhancing T-cell reconstitution before or after hematopoietic stem cell transplantation (ACES): a two-arm, open label phase II interventional trial of pediatric patients with risk factor assessment
Source: Nat Commun. 2024 Apr 18;15:3258. doi: 10.1038/s41467-024-47057-2 (PMC11026387; doi:10.1038/s41467-024-47057-2)
Supplement: Supplementary file 3 — Description of Additional Supplementary Files [file 41467_2024_47057_MOESM3_ESM.pdf]

Description of Additional Supplementary Files

Supplementary Data 1

CMV-associated public clonotypes in patients post-VST infusion.
