# Supplementary material for: Antiviral cellular therapy for enhancing T-cell reconstitution before or after hematopoietic stem cell transplantation (ACES): a two-arm, open label phase II interventional trial of pediatric patients with risk factor assessment
Source: Nat Commun. 2024 Apr 18;15:3258. doi: 10.1038/s41467-024-47057-2 (PMC11026387; doi:10.1038/s41467-024-47057-2)
Supplement: Supplementary file 5 — Reporting Summary [file 41467_2024_47057_MOESM5_ESM.pdf]

nature portfolio

Corresponding author(s): Michael Pulsipher

Last updated by author(s): 02/05/2024

Reporting Summary

Nature Portfolio wishes to improve the reproducibility of the work that we publish. This form provides structure for consistency and transparency in reporting. For further information on Nature Portfolio policies, see our Editorial Policies and the Editorial Policy Checklist.

Statistics

For all statistical analyses, confirm that the following items are present in the figure legend, table legend, main text, or Methods section.

n/a	Confirmed	
☐	☒	The exact sample size (*n*) for each experimental group/condition, given as a discrete number and unit of measurement
☐	☒	A statement on whether measurements were taken from distinct samples or whether the same sample was measured repeatedly
☐	☒	The statistical test(s) used AND whether they are one- or two-sided<br>*Only common tests should be described solely by name; describe more complex techniques in the Methods section.*
☐	☒	A description of all covariates tested
☐	☒	A description of any assumptions or corrections, such as tests of normality and adjustment for multiple comparisons
☐	☒	A full description of the statistical parameters including central tendency (e.g. means) or other basic estimates (e.g. regression coefficient) AND variation (e.g. standard deviation) or associated estimates of uncertainty (e.g. confidence intervals)
☐	☒	For null hypothesis testing, the test statistic (e.g. *F*, *t*, *r*) with confidence intervals, effect sizes, degrees of freedom and *P* value noted<br>*Give P values as exact values whenever suitable.*
☒	☐	For Bayesian analysis, information on the choice of priors and Markov chain Monte Carlo settings
☐	☒	For hierarchical and complex designs, identification of the appropriate level for tests and full reporting of outcomes
☒	☐	Estimates of effect sizes (e.g. Cohen's *d*, Pearson's *r*), indicating how they were calculated

Our web collection on statistics for biologists contains articles on many of the points above.

Software and code

Policy information about availability of computer code

Data collection	Clinical data was collected in Microsoft Excel (ver 16.72, Redmond, WA) and Graphpad prism (ver. 9.3.1, San Diego, CA).<br>Cytokine assays: All plasma samples were aquired on a MAGPIX system (Luminex, Austin, TX).<br>Flow cytometry: Samples were acquired on a CytoFLEX cytometer (Beckman Coulter, Brea, CA), and sorted samples were run on a CytoFlex SRT Cell Sorter (Beckman Coulter, Brea, CA).<br>ELISpot results were obtained using a KS Elispot Reader (Carl Zeiss, Inc., Thornwood, NY), Software Version 5.9.16.
Data analysis	Flow cytometry data was analyzed with FlowJo X (FlowJo LLC, Ashland, OR). TCR tracking and Shannon Diversity indices were calculated in Python. VDJ junction mapping and clonotype assembly and annotation using the assembled contigs from migec was done with mixcr (v3.0.13) using the analyze amplicon routine. The forest plot as well as other graphical presentations were contructed with Stata version 17, while statistical analyses including the limxed models were performed using R version 4.2.1.

For manuscripts utilizing custom algorithms or software that are central to the research but not yet described in published literature, software must be made available to editors and reviewers. We strongly encourage code deposition in a community repository (e.g. GitHub). See the Nature Portfolio guidelines for submitting code & software for further information.

Data

Policy information about availability of data

All manuscripts must include a data availability statement. This statement should provide the following information, where applicable:

- Accession codes, unique identifiers, or web links for publicly available datasets
- A description of any restrictions on data availability
- For clinical datasets or third party data, please ensure that the statement adheres to our policy

The datasets generated during and/or analyzed during the current study have been enclosed in supplemental data. The flow cytometry and TCR sequencing datasets are available on Zenodo under the following link: https://zenodo.org/records/10562383.
All remaining data is enclosed within the article, supplementary information, and source data files.
De-identified patient data is enclosed in Supplementary Table 1 . The study protocol and statistical analysis plan are included in the supplementary data files.

Research involving human participants, their data, or biological material

Policy information about studies with human participants or human data. See also policy information about sex, gender (identity/presentation), and sexual orientation and race, ethnicity and racism.

Reporting on sex and gender	This study involved recruitment and treatment of pediatric patients with refractory viral infections after bone marrow transplantation or due to inborn errors of immunity with partially HLA-matched virus-specific T cells. Patients of all sexes/genders were eligible for recruitment (25 male, 26 female patients).
Reporting on race, ethnicity, or other socially relevant groupings	Patients of all races/ethnicities were eligible for enrollment on this study. We do not report on race/ethnicity or other socially relevant groupings in the manuscript, though this data is available and may be included if deemed necessary.
Population characteristics	Pediatric and young adult patients (median 8 years of age, range 1 month - 23 years) with refractory viral infections either after bone marrow transplantation (n=47) or pre-transplant with inborn errors of immunity (n=4) were treated on this study. Underlying disorders in those who underwent transplant included malignancy (n=22), inborn errors of immunity (n=20), and other non-malignant conditions (n=9).
Recruitment	The subjects were recruited on the ACES study (NCT03475212) at 22 participating sites within the Pediatric Cellular Therapy and Transplantation Consortium. Eligibility criteria for this study were: patients with primary immunodeficiency or recipients of allogeneic hematopoietic stem cell transplantation with refractory infection with cytomegalovirus, Epstein-Barr virus, and/or adenovirus, and no other uncontrolled infections, high grade graft versus host disease (>grade II), or relapse of malignancy. As a pediatric study, subject were limited to those 0-25 years of age. Patients of all genders and ethnicities were eligible for enrollment, with actual subject enrollment limited only by the patient demographics of the 22 centers participating in this study. The study protocol is attached in Supplemental Data.
Ethics oversight	This study was approved at a central institutional review board (Children's Hospital of Los Angeles, 4650 Sunset Blvd, Los Angeles CA, USA) as well as the institutional review boards of participating sites:<br>Children's National Hospital<br>St Jude's Research Children's Hospital<br>Children's Hospital of Atlanta<br>University of Southern California, Los Angeles<br>University of California San Francisco<br>Children's Hospital of Colorado<br>Helen DeVos Children's Hospital<br>Phoenix Children's Hospital<br>Virginia Commonwealth University<br>Fred Hutchinson / Seattle Children's Hospital<br>Columbia University Medical Center<br>Children's Mercy Medical Center<br>Levine Children's Hospital<br>Oregon Health and Science University<br>Lucile Packard Children's Hospital, Stanford University<br>C.S. Mott Children's Hospital, University of Michigan<br>University of Minnesota MHealth Fairview Masonic Children's Hospital<br>University of Texas, Southwestern Medical Center Dallas<br>Washington University<br>Duke University<br>Dana Farber Institute and Boston Children's Hospital<br>City of Hope<br>Lurie Children's Hospital<br>Medical University of S. Carolina<br>Methodist Hospital<br>Riley Children's - IU<br>Roswell Park

Tufts Children's Hospital
Yale University
Cleveland Clinic Foundation
Children's Hospital of Philadelphia

Study data was prospectively reviewed by the Drug safety monitoring board of the Pediatric Transplantation and Cellular Therapy Consortium (PTCTC).

Note that full information on the approval of the study protocol must also be provided in the manuscript.

Field-specific reporting

Please select the one below that is the best fit for your research. If you are not sure, read the appropriate sections before making your selection.

- [x] Life sciences
- [ ] Behavioural & social sciences
- [ ] Ecological, evolutionary & environmental sciences

For a reference copy of the document with all sections, see nature.com/documents/nr-reporting-summary-flat.pdf

Life sciences study design

All studies must disclose on these points even when the disclosure is negative.

Sample size	Fifty-one recipients, and 40 healthy donors were utilized for this study. Goal recruitment of 50-60 patients was determined as follows: Efficacy, as measured by the proportion of patients achieving complete or partial response (see protocol definitions), will be assessed independently in each of the three strata CMV, EBV, and Adenovirus) The two primary strata (CMV and Adenovirus) will enroll a minimum of 20 and maximum of 30 evaluable patients. Under the null hypothesis that VST are not active, we assume that that the true spontaneous antiviral response rate will be no more than 20%. Thus in each of the two primary strata, based on an exact one-sided one-sample test of proportions of the null hypothesis that the response rate is ≤20%, with Type I error no greater than 5% and with a sample size of 20, there will be at least 90% power to detect an improvement in response rate to 52%. With a sample size of 30 the detectable improvement is to 47%. Hence this study has sufficient power to detect clinical important improvements in response rate due to VST in each of the primary strata.
Data exclusions	There was no data exclusion
Replication	All immunoassays were performed in replicate (triplicate where cells allowed) in order to ensure result accuracy. All replicate results were consistent given the result variability of the individual assays (ELISpot, Flow cytometry, and multiplex cytokine analysis). TCR sequencing was performed without replicates, with a maximal number of cells feasible based on sample availability.
Randomization	As an open label study, randomization was not performed.
Blinding	The study intervention was not blinded due to the open label nature of this study. Blinded analysis of antiviral responses was performed by protocol committee members using coded clinical data to reduce bias, with a minimum of 2 reviewers evaluating each dataset.

Reporting for specific materials, systems and methods

We require information from authors about some types of materials, experimental systems and methods used in many studies. Here, indicate whether each material, system or method listed is relevant to your study. If you are not sure if a list item applies to your research, read the appropriate section before selecting a response.

Materials & experimental systems

n/a	Involved in the study	
[ ]	[x]	Antibodies
[x]	[ ]	Eukaryotic cell lines
[x]	[ ]	Palaeontology and archaeology
[x]	[ ]	Animals and other organisms
[ ]	[x]	Clinical data
[x]	[ ]	Dual use research of concern
[x]	[ ]	Plants

Methods

n/a	Involved in the study	
[x]	[ ]	ChIP-seq
[ ]	[x]	Flow cytometry
[x]	[ ]	MRI-based neuroimaging

Antibodies

Antibodies used

All antibody panels are listed in supplemental data.

Intracellular cytokine panel:
Brilliant Violet 421™ anti-human CD8a Antibody/ BioLegend, #301036, RPA-T8, BV421, 5ul/1E6 cells
LIVE/DEAD Fixable Aqua Dead Cell Stain Kit, for 405 nm excitation, Invitrogen, L34966, Aqua, 1ul/ml
Brilliant Violet 605 anti-human CD4 Antibody, BioLegend, #317438, OKT4, BV605, 5ul/1E6 cells

Brilliant Violet 650 anti-human CD56 (NCAM) Antibody BioLegend, #318344, HCD56, BV650, 5ul/1E6 cells
Brilliant Violet 785 anti-human CD3 Antibody BioLegend, #317330, OKT3, BV785, 5ul/1E6 cells
PE anti-human TNF-α Antibody, BioLegend, #502909, MAb11, PE, 5ul/1E6 cells
PE/Dazzle 594 anti-human CD45RO Antibody, BioLegend, #304248, UCHL1, PE/Dazzle 594, 5ul/1E6 cells
PerCP/Cyanine5.5 anti-human TCR α/β Antibody, BioLegend, #306724, IP26, PerCP/Cyanine5.5, 5ul/1E6 cells
PE/Cyanine7 anti-human CD107a (LAMP-1) Antibody, BioLegend, #328618, H4A3, PE/Cyanine7 5ul/1E6 cells
APC anti-human IFN-γ Antibody, BioLegend, #502512, 4S.B3, APC, 5ul/1E6 cells
Alexa Fluor 700 anti-human CD197 (CCR7) Antibody, BioLegend, #353244, G043H7, AF700, 5ul/1E6 cells
APC/Fire 750 anti-human TCR γ/δ Antibody, BioLegend, #331228, B1, APC/Fire 750, 5ul/1E6 cells

Anti-HLA antibodies:
A2, Milteyni, 130-118-969, REA517 FITC 2 ul / 200k cells
A3, Milteyni 130-115-739, REA950 FITC 2 ul / 200k cells
A28:A2, Milteyni A68, A69 130-099-601, REA142 FITC 10 ul / 200k cells
A9, Milteyni, 130-099-524, REA127" FITC 2 ul / 200k cells
A30:A31, One Lambda N/A Steptavidin FITC: 405201 Biotin (+ Streptavidin FITC and PE conjugates) 2ul/200k cells
B7, Thermo(FITC) B703# MA1-82180, BB7.1 FITC 10 ul / 200k cells
B12, Milteyni, 130-099-862, REA138 FITC 30 tests in 300ul
Bw4, Milteyni, 130-103-846, REA274 FITC 10ul/200k cells
Bw6 Milteyni, 130-123-264 REA143 FITC 2 ul / 200k cells
B7, B27 Miltenyi, 130-120-234 REA176 FITC 2 ul / 200k cells

Release flow cytometry panel:
CD45 APC Miltenyi, 130-110-633 REA747 1ul/2E5 cells
CD3 PerCP Vio 700 Miltenyi, 130-113-141 REA613 1ul/2E5 cells
CD19 FITC Miltenyi, 130-113-645 REA675 1ul/2E5 cells
CD14 VioBlue Miltenyi, 130-110-524 REA599 2ul/2E5 cells
CD4 PE Vio770 Miltenyi, 130-113-227 REA623 1ul/2E5 cells
CD8 APC Vio770 Miltenyi, 130-110-681 REA734 1ul/2E5 cells
CD16 PE Miltenyi, 130-113-393 REA423 1ul/2E5 cells
CD 56 PE Miltenyi, 130-113-312 REA196 1ul/2E5 cells
CD83 PE Miltenyi, 130-110-503 REA714 1ul/2E5 cells
TCRab FITC Miltenyi, 130-113-538 RES652 1ul/2E5 cells
TCRgd PE Miltenyi, 130-113-512 REA591 1ul/2E5 cells
CD45RO PE Miltenyi, 130-113-559 REA611 2ul/2E5 cells
CCR7 FITC Miltenyi, 130-120-468 REA546 2ul/2E5 cells
CD95 APC BD Bio, 558814 DX2 2ul/2E5 cells
CD62 VioBlue Miltenyi, 130-113-622 145/15 2ul/2E5 cells
HLA DR FITC Miltenyi, 30-111-788 REA805 1ul/2E5 cells

Validation

Anti-cytokine antibodies underwent titration to establish optimal staining index for human leukocytes as listed above. All other antibodies were utilized per manufacturer recommendations. All antibody dilutions are listed above and in the supplemental data.

Clinical data

Policy information about clinical studies
All manuscripts should comply with the ICMJEguidelines for publication of clinical research and a completedCONSORT checklist must be included with all submissions.

Clinical trial registration

NCT03475212

Study protocol

The full clinical protocol of the ACES trial may be obtained from the authors at reasonable request.

Data collection

Data from the subjects was collected by investigators at the each participating clinical sites between June 2019 to December 2021. between September-December of 2019. Correlative studies and data analysis was analyzed at Children's National Hospital and Children's Hospital of Los Angeles.

Outcomes

The primary endpoints of this study were safety of T cell infusion, efficacy against targeted viruses based on blood viral PCR at 1 month post infusion, and feasibility of third-party T cell infusion (ability to identify a suitable banked product). Secondary endpoints were immune reconstitution against targeted viruses, persistence of infused cells, and overall survival at 1 year post infusion. These outcomes were assessed by investigators at each study site via clinical lab testing as well as correlative assays in blood performed centrally at Children's National Hospital.

Flow Cytometry

Plots

Confirm that:

- [x] The axis labels state the marker and fluorochrome used (e.g. CD4-FITC).
- [x] The axis scales are clearly visible. Include numbers along axes only for bottom left plot of group (a 'group' is an analysis of identical markers).
- [x] All plots are contour plots with outliers or pseudocolor plots.
- [x] A numerical value for number of cells or percentage (with statistics) is provided.

Methodology

Sample preparation	All cells were obtained from whole blood via Ficoll centrifugation.
Instrument	All samples were run on a Cytoflex Cytometry (Beckman Coulter) or CytoFlex SRT Cell Sorter
Software	Data was analyzed with FlowJo X (FlowJo LLC, Ashland, OR).
Cell population abundance	For this study, flow cytometry was used to identify antigen-specific T cells targeting CMV, EBV, and adenovirus. Antigen-specific cells were identified using intracellular cytokine staining and HLA multimers. Peptide stimulation was used to induce cytokine expression, alongside control conditions (actin peptides and staphylococcal enterotoxin B). As these experiments focused on rare antigen specific populations, T cell specificity was determined by comparison of cytokine-expressing populations following experimental peptide library stimulation with the control conditions.
Gating strategy	Gating was established using our laboratory's established protocol and gating template. Full gating strategy is included in Supplemental Figure 9.

- [x] Tick this box to confirm that a figure exemplifying the gating strategy is provided in the Supplementary Information.
