# Supplementary material for: Antiviral cellular therapy for enhancing T-cell reconstitution before or after hematopoietic stem cell transplantation (ACES): a two-arm, open label phase II interventional trial of pediatric patients with risk factor assessment
Source: Nat Commun. 2024 Apr 18;15:3258. doi: 10.1038/s41467-024-47057-2 (PMC11026387; doi:10.1038/s41467-024-47057-2)
Supplement: Supplementary file 2 — Peer Review File [file 41467_2024_47057_MOESM2_ESM.pdf]

nature portfolio

Peer Review File

Antiviral Cellular Therapy for Enhancing T-cell Reconstitution Before or After Hematopoietic Stem Cell Transplantation (ACES): a two-arm, open label phase II interventional trial of pediatric patients with risk factor assessment

Open Access This file is licensed under a Creative Commons Attribution 4.0 International License, which permits use, sharing, adaptation, distribution and reproduction in any medium or format, as long as you give appropriate credit to the original author(s) and the source, provide a link to the Creative Commons license, and indicate if changes were made. In the cases where the authors are anonymous, such as is the case for the reports of anonymous peer reviewers, author attribution should be to 'Anonymous Referee' followed by a clear attribution to the source work. The images or other third party material in this file are included in the article's Creative Commons license, unless indicated otherwise in a credit line to the material. If material is not included in the article's Creative Commons license and your intended use is not permitted by statutory regulation or exceeds the permitted use, you will need to obtain permission directly from the copyright holder. To view a copy of this license, visit http://creativecommons.org/licenses/by/4.0/.

Editorial Note: This manuscript has been previously reviewed at another journal that is not operating a transparent peer review scheme. This document only contains reviewer comments and rebuttal letters for versions considered at *Nature Communications*.

REVIEWERS' COMMENTS

Reviewer #1 (Remarks to the Author):

The authors addressed my concerns and I believe the ms is suitable for publication

Reviewer #2 (Remarks to the Author):

In this revised manuscript, the ACES Study team has added additional clarity, data and contextual analysis to their original findings. The observations made here have significant value to the field of pediatric transplantation and propel our ability to manage some of the most complex infectious complications in this population. The authors have addressed all of the reviewer's comments to the best of their ability, appropriately acknowledging the limitations of the data generated as part of a phase II clinical trial. This is an impressive report and the authors should be complemented for their contribution to the care of this high risk population. One note is that I could not find the full author list in the manuscript.
